# Adherence to participant flow diagrams in trials on postoperative pain management after total hip and knee arthroplasty: a methodological review

**DOI:** 10.1186/s13063-021-05233-5

**Published:** 2021-04-14

**Authors:** Thea Nørgaard Rønsbo, Jens Laigaard, Casper Pedersen, Ole Mathiesen, Anders Peder Højer Karlsen

**Affiliations:** 1 Department of Anaesthesiology, Centre for Anaesthesiological Research, Zealand University Hospital, Lykkebækvej 1, 4600 Køge, Denmark; 2 Department of Clinical Medicine, University of Copenhagen, Copenhagen, Denmark

## Abstract

**Background:**

The Consolidated Standards of Reporting Trials (CONSORT) statement aims to improve transparent reporting of randomised clinical trials. It comprises a participant flow diagram with the reporting of essential numbers for enrolment, allocation and analyses. We aimed to quantify the use of participant flow diagrams in randomised clinical trials on postoperative pain management after total hip and knee arthroplasty.

**Methods:**

We searched PubMed, Embase and CENTRAL up till January 2020. The primary outcome was the proportion of trials with adequate reporting of participant flow diagrams, defined as reporting of number of participants screened for eligibility, randomised and included in the primary analysis. Secondary outcomes were recruitment (randomised:screened) and retention (analysed:randomised) rates, reporting of a statistical strategy, reasons for exclusion from the primary analysis and handling of missing outcome data. Trends over time were assessed with statistical process control.

**Results:**

Of the 570 included trials, we found adequate reporting in 240 (42%). Reporting with participant flow diagram increased significantly over time. Median recruitment was 73% (IQR 44–91%), and retention was 97% (IQR 93–100%). These rates did not change over time. Trials with adequate reporting of participant flow were more likely to report a statistical strategy (41% vs 8%), reasons for post-randomisation exclusions (100% vs 55%) and handling of missing outcome data (14% vs 6%).

**Conclusions:**

Adherence to participant flow diagrams for RCTs has increased significantly over time. Still, there is room for improvement of adequate reporting of flow diagrams, to increase transparency of trials details.

**Supplementary Information:**

The online version contains supplementary material available at 10.1186/s13063-021-05233-5.

## Background

Transparent reporting in randomised clinical trials (RCTs) is essential to trial validity and the subsequent implementation of results in clinical settings. Insufficient trial reporting has led to establishment of the first Consolidated Standards of Reporting Trials (CONSORT) statement in 1996 [1, 2].

The most recent version CONSORT 2010 provides a set of evidence-based standards for transparent and sufficient reporting of randomised trials [3–5]. The statement comprises a checklist and a participant flow diagram. A sufficient participant flow diagram includes the number of participants screened, randomised and analysed for the primary outcome and the number of, and reasons for, post-randomisation exclusions in each trial arm.

RCTs should be pragmatic and optimally reflect clinical settings [6–8]. The CONSORT participant flow diagram enables the reader to assess discrepancies between eligible and included individuals and reasons for post-randomisation exclusions. In pain management research, adequate flow diagrams are particularly important, because inadequate pain relief or adverse events can cause dropouts. Without an adequate flow diagram, the intervention may be misinterpreted as more beneficial than the true effect. Postoperative pain treatment after total hip and knee arthroplasty (THA and TKA) are among the most frequent elective procedures with high risk of postoperative pain. However, heterogenic trial designs and insufficient reporting has hampered aggregation and interpretation of intervention effects in meta-analyses, which is why methodological improvements are needed [9–11].

With this review, we aimed to investigate the use of participant flow diagrams and adequacy in the reporting of participant flow in RCTs on postoperative pain management after THA and TKA.

## Methods

This methodological review was structured in accordance with the Preferred Reporting Items for Systematic Reviews and Meta-Analysis (PRISMA) statement, though leaving out meta-analyses and bias evaluation, as these were deemed irrelevant for the aim of this review [12]. The protocol for this and a parallel review [13] was registered prior to study commencement at International Prospective Register of Systematic Reviews (PROSPERO) (identifier CRD42020151317).

### Literature search and eligibility criteria

A systematic search was conducted in PubMed, Embase and CENTRAL. The search strategy is in Additional file 1. The last search was conducted January 7, 2020.

Eligible studies were RCTs investigating perioperative analgesic interventions for postoperative pain management in adults aged 18 years or above undergoing total hip or knee arthroplasty, written in English and regardless of year of publication. Trials comparing multiple interventions as well as trials comparing one or several interventions with a control group were included. Quasi-randomised trials, conference abstracts and observational studies were excluded. We excluded RCTs that included hemi-arthroplasties and arthroplasties due to fractures.

### Data extraction

Records up to June 2019 were screened for a previous review (PROSPERO Identifier CRD42019125691). For the updated search, two authors (CP and AK) assessed RCTs for eligibility independently. Four authors (AK, CP, JL and TR) independently extracted data for the first 20 RCTs to secure uniformity. Data for the remaining RCTs were extracted by two independent authors (CP, JL or TR). Discrepancies were solved involving a senior author (AK or OM). Data concerning participant flow were extracted into Excel. As this review investigates reporting standards in RCTs, the authors have not been contacted for missing data.

### Outcomes

The primary outcome was to quantify adequacy in reporting of participant flow diagrams in the included RCTs. Reporting was defined as adequate if trials reported the number of participants screened for eligibility, randomised and included in the primary analysis for each group.

Secondary outcomes were as follows: (1) recruitment (randomised:screened) and retention (analysed:randomised) rates, (2) proportion of trials reporting a statistical strategy for the primary analysis (intention-to-treat, per protocol or modified intention-to-treat analyses), (3) reasons for exclusions from the primary analysis and (4) handling of missing outcome data.

Further, we assessed trends over time and performed subgroup analyses for continental, interventional and procedural differences.

### Statistical analyses

Data were described by numbers and percentages or median and interquartile range. Differences between trials with and without patient flow diagrams for choice of primary outcome, statistical strategy and handling of missing data were assessed with chi-square test with *p* <  0.05 as significance level. All other comparisons were assessed qualitatively.

Trends over time for adequate reporting of participant flow were evaluated with statistical process control. Control charts were used to test for non-random variation [14], indicated by unusually long runs at one side of the mean or unusually few crossings over the mean. Cutoff values for runs and crossings are calculated based on the number of observations in the chart and correspond to a significance level of 0.05 [15, 16].

In a post hoc multivariate regression, we analysed the effect of (i) publication year, (ii) number of participants (per trial arm), (iii) journal impact factor, (iv) multicentricity and (v) prospective online trial registration on (1) the probability of having an adequately reported flow diagram, (2) recruitment rate and (3) retention rate. We used logistic regression for adequacy in flow diagrams and linear regression for recruitment and retention rates. We used complete-case analyses and considered *p* values < 0.05 as significant. R i386 version 3.6. with add-on Qicharts2 was used for process control and statistical analyses [17, 18].

## Results

### Characteristics of the RCTs

**Fig. 1:**
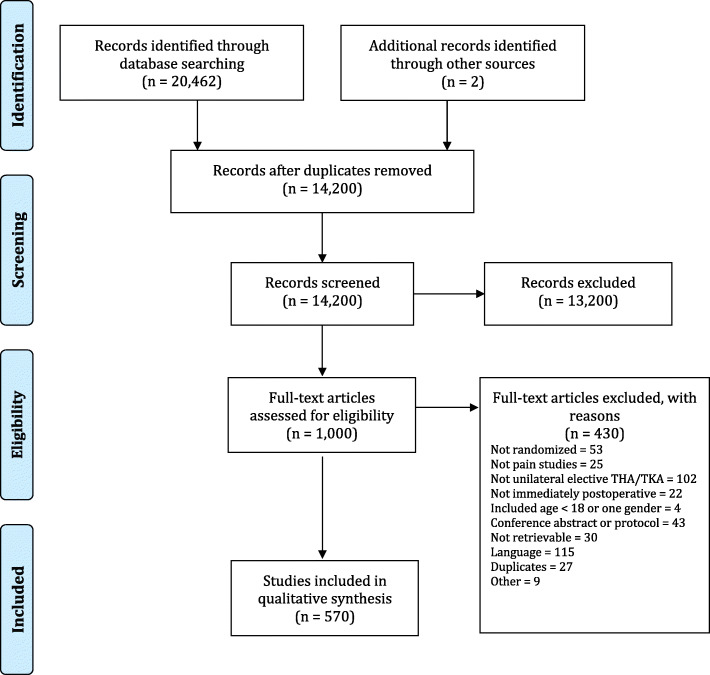
PRISMA flow diagram. PRISMA flow chart showing the selection process; 570 trials were included. The same screening was used for this and the parallel review under same PROSPERO CRD-identifier

We identified 20,646 articles, of which 1000 were eligible for full text screening and 570 were included (Fig. 1, Additional file 2). Additional file 3 shows the details for excluded trials.

Characteristics of the included trials are summarised in Table 1. The majority of trials were published in Europe (40%), Asia (31%) and North America (24%). Of the included trials, 65% concerned postoperative pain management following TKA, and 59% were published between 2011 and 2020. Less than 100 participants were randomised in 75% of trials.

**Table 1:** Trial characteristics and reporting of flow diagrams

	*Proportion of trials with adequate flow diagram (%)*
All trials	240 of 570 *(42%)*
*Publication year*	
1981–1990	0 of 13 *(0%)*
1991–2000	0 of 71 *(0%)*
2001–2010	29 of 148 *(20%)*
2011–2020	211 of 338 *(62%)*
*Continent*	
Europe	86 of 229 *(38%)*
Asia	81 of 174 *(47%)*
North America	64 of 137 *(47%)*
Australia	5 of 14 *(36%)*
Africa	2 of 8 *(25%)*
South America	2 of 8 *(25%)*
*Type of surgery*	
TKA	165 of 373 *(44%)*
THA	64 of 153 *(42%)*
TKA and THA	11 of 44 *(25%)*
*Trial size*	
0–50	44 of 176 *(25%)*
51–100	109 of 252 *(43%)*
101–150	40 of 72 *(56%)*
151–200	25 of 38 *(66%)*
201–250	11 of 16 *(69%)*
> 250	11 of 17 *(65%)*
*Tested intervention*	
Regional blocks	54 of 128 *(42%)*
Local infiltration analgesia	49 of 88 *(56%)*
Neuraxial methods	8 of 80 *(10%)*
Systemic analgesia	62 of 118 *(53%)*
Multiple/mixed interventions	67 of 156 *(43%)*

### Reporting of participant flow

**Fig. 2:**
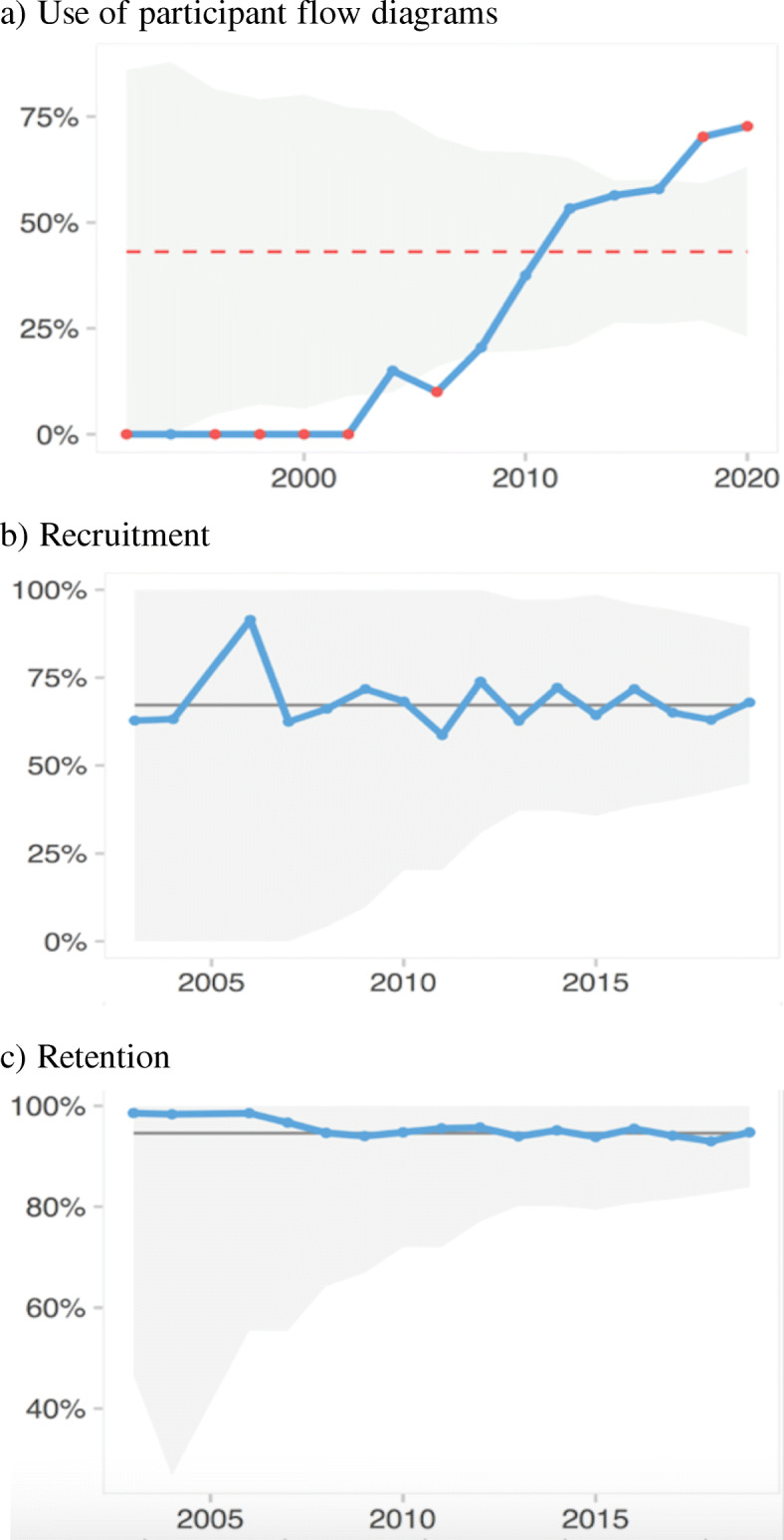
Trends over time for use of flow diagram and recruitment and retention rates. Control charts: for significant trends, the mean is shown as a red stippled line. The grey area marks the control limits. Red points outside the control limits indicate an unstable process and thereby a trend. **a** The use of adequate patient flow diagrams: control chart showing a significant increase in the use of adequate flow diagrams. Flow diagrams were deemed adequate when they reported the number of participants screened for eligibility, randomised and included in primary analysis for each trial arm. **b** Recruitment over time: control chart showing no significant trends for recruitment rate over time. We defined recruitment as the rate between number of randomised participants and participants screened for eligibility. **c** Retention over time: control chart showing no significant trends for retention rate over time. We defined retention as the rate between number of participants included in the primary analysis and of randomised participants

A participant flow diagram was reported in 258 trials, and 240 (42%) trials reported adequately on the number of participants screened, randomised and analysed. Adequate reporting of participant flow diagrams increased significantly over time (Fig. 2).

In total, the 240 RCTs using adequate participant flow diagrams screened 53,123 individuals, randomised 27,432 participants and analysed 25,740. The median recruitment rate was 73% (IQR 44–91%). The median retention rate was 97% (IQR 93–100%). None of these rates changed significantly over time (Table 2, Fig. 2). Subgroup analyses are listed in Additional file 4.

**Table 2:** Recruitment and retention in trials with adequate flow diagrams

	All trials Median (IQR)	THA trials Median (IQR)	TKA trials Median (IQR)
Recruitment (randomised:screened)	73% (44–91%)	68% (44–94%)	74% (43–90%)
Retention (analysed:randomised)	97% (93–100%)	98% (95–100%)	97% (92–100%)
Trials with < 95% retention	33%	25%	35%

Data for recruitment and retention were non-parametrically distributed

Recruitment was high for both THA and TKA trials

### Reported analyses and reasons for exclusion

Trials with adequate flow diagrams were significantly better at reporting the strategies for statistical analyses and for handling of missing outcome data (Table 3).

Reported reasons for exclusion of randomised participants are listed in Table 3. Trials with a participant flow diagram were more likely to report reasons for exclusion.

**Table 3:** Data analysis and missing data

	Trials with flow diagrams (240)	Trials without flow diagrams (330)	*p* value
**Strategy for primary analysis**	*n (%)*	*n (%)*	
Intention-to-treat	81 *(34%)*	23 *(7%)*	< 0.0001
Modified intention-to-treat	3 *(1%)*	4 *(1%)*	1
Per-protocol	15 *(6%)*	1 *(0%)*	< 0.0001
Not mentioned	141 *(59%)*	302 *(92%)*	< 0.0001
**Handling of missing outcome data**			
Not mentioned	207 *(86%)*	309 *(94%)*	0.001
Excluded from analysis	15 *(6%)*	14 *(4%)*	0.27
Imputed	14 *(6%)*	3 *(1%)*	0.0007
Last observation carried forward	4 *(2%)*	4 *(1%)*	0.32
**Reasons for post-randomisation exclusions**			
Non-adherence to the protocol^a^	90 *(35%)*	106 *(32%)*	N/A
None excluded	81 (*32%)*	43 *(13%)*	N/A
Withdrawal of consent incl. dropouts/attrition	79 *(31%)*	38 *(12%)*	N/A
Did not receive intervention	51 *(20%)*	26 *(8%)*	N/A
Intractable pain/non-protocolled analgesics^b^	34 *(13%)*	44 *(13%)*	N/A
Wrongful inclusion	25 *(10%)*	12 *(4%)*	N/A
No outcome data	24 *(9%)*	19 *(6%)*	N/A
Group size too small to analyse	1 *(0%)*	0 *(0%)*	N/A
Not mentioned	0 *(0%)*	147 *(45%)*	N/A

RCTs may report multiple reasons for exclusion, why the sum is more than 100%

^a^Stopped taking medication/protocol violation, etc.

^b^Required rescue block, other opioids, etc.

*p* values calculated by chi-square test

### Post hoc multivariate logistic regression

We found that increasing year of publication, higher number of participants and higher journal impact factor were associated with reporting of adequate flow diagrams. Higher impact factor and prospective online trial registration were associated with a lower recruitment rate. A higher number of participants were associated with a lower retention rate (Table 4).

**Table 4:** Post-hoc analyses: multivariate regression

**Adequate flow diagrams (*n*** **= 488)**	Adjusted odds ratio (95% CI)	
Year published	1.20 (1.14 to 1.27)	*p* < 0.001
Number of participants	1.01 (1.00 to 1.02)	*p* = 0.005
Journal impact factor	1.70 (1.44 to 2.02)	*p* < 0.001
Multicentre study	0.45 (0.18 to 0.11)	*p* = 0.083
Prospective online trial registration	2.44 (1.26 to 4.72)	*p* = 0.008
**Recruitment rate in percent (*n*** **= 256)**	Coefficient (95% CI)	
Year published	0.03 (− 0.06 to 0.12)	*p* = 0.517
Number of participants	0.03 (− 0.06 to 0.12)	*p* = 0.544
Journal impact factor	− 4.29 (− 6.16 to − 2.43)	*p* < 0.001
Multicentre study	− 3.66 (− 14.79 to 7.43)	*p* = 0.518
Prospective online trial registration	− 8.01 (− 15.09 to − 0.93)	*p* = 0.027
**Retention rate in percent (*n*** **= 363)**	Coefficient (95% CI)	
Year published	− 0.01 (− 0.18 to 0.16)	*p* = 0.924
Number of participants	− 0.03 (− 0.05 to 0.00)	*p* = 0.022
Journal impact factor	0.25 (− 0.25 to 0.75)	*p* = 0.332
Multicentre study	− 0.67 (− 3.32 to 1.98)	*p* = 0.620
Prospective online trial registration	− 0.21 (− 2.21 to 1.78)	*p* = 0.834

Eighty-two values were missing for ‘Journal impact factor’ in a non-random fashion (including 68 trials published before 1999); thus, we refrained from imputing, and analyses were performed only on complete cases. The number of participants is reported per trial arm.

## Discussion

Adequate participant flow diagrams stating the number of participants screened, randomised and analysed were reported in 240 of the 570 included trials (42%). Adequate reporting increased significantly over time and was higher in large trials and high impact journals. Rates for recruitment and retention were stable over time. Reporting of strategy for handling of missing data was low in both trials with and without participant flow diagrams, although significantly better in trials with adequately reported participant flow. Trials with adequate flow diagrams were also significantly better to report the statistical analysis for the primary outcome, used more appropriate methods and sufficiently described reasons for exclusion of participants.

Studies of poor methodological quality are more likely to overestimate benefits [19]. Adherence to CONSORT improves the adequacy of reporting and is associated with higher methodological quality [6, 20]. A recent review of periodontological trials published between 2011 and 2016 found that 60–79% of published trials adhered to the CONSORT checklist and reported a participant flow diagram, which is higher than for our review [21]. Similarly, a review from 2009 investigating the reporting of recruitment and retention in top journals found that 79% of trials had flow diagrams. However, 40% of these would have been deemed insufficient in our review as they failed to report participants assessed for eligibility. We found an association between having an adequate flow diagram and publication in higher impact journals. Further, the 2009 review reported a high rate of eligible persons not wanting to participate and drop-outs for unknown reasons though retention of randomised participants was generally high [22]. In our review, 31% of trials with adequate flow diagrams reported at least one case of withdrawal of consent. Our post hoc analysis demonstrated significant lower retention in larger trials, but the point estimate was close to zero and the results were hardly of clinical relevance. These findings indicate a potential benefit in making trial participation more participant-friendly. A systematic review from 2020 investigating burdens of participating in RCTs found that participation was associated with both psychological, physical and financial distress. The authors concluded that these burdens could be anticipated by conducting minimally disruptive clinical research, which in terms could improve trial recruitment and retention [23]. A third of trials included in our review with flow diagrams had at least one wrongful inclusion or participant that did not receive the intervention. Recruitment, randomisation and treatment errors are common, even for trials published in top journals, and should be reported to increase transparency [24].

We found a median recruitment rate of 73%, which may be considered relatively high. We were unable to assess whether this was partly due to some trials having pre-excluded participants that were obviously non-includable. Trials in other research areas have been criticised for having clinically unrepresentative populations hampering the clinical relevance and implementation of trials results [7, 25]. Adequate flow diagrams facilitate detection of left-out patient subgroups, which makes it easier to evaluate trial generalisability (external validity) [22, 26, 27]. Broadening eligibility criteria in clinical pragmatic trial designs and avoiding excessive exclusion criteria can make the population more representative [28, 29].

Retention rates were overall sufficiently described in most trials. It is however remarkable that one third of the trials had more than 5% of participants left out of the primary analyses. These numbers weaken reliability of trial results, unless such patients are missing at random which most often is not the case. Therefore, trials should ideally state how missing data were handled [30]. It is disappointing that the majority of the trials did not inform on handling of missing data, although these numbers were significantly improved for trials with participant flow diagrams.

The primary analysis of data in RCTs should preserve patients for analyses according to the assigned group. This is designated the ‘intention-to-treat’ principle. This minimises the influence of withdrawals and patients lost to follow-up and likely prevents type 1 errors [31]. Furthermore, leaving out reporting of the statistical analyses impairs the reader’s quality assessment of trial findings. The intention-to-treat analysis is the optimal way to preserve randomisation and enhance internal validity [32] (though a cross-sectional study found that the modified intention-to-treat provided an equal methodological quality [33]). It is therefore somewhat discouraging, that nearly six of 10 trials did not report on handling of missing data in our review. Again, we found that for trials presenting patient flow diagrams, this number was significantly improved compared to trials without. Such improvement possibly does not relate to the patient flow diagram per se but can be seen as an overall improved methodological quality of trials presenting patient flow diagrams.

## Strengths and limitations

We did not assess unpublished trials which could potentially skew our analyses, and we left out non-English publications which is a limitation to the continental subgroup analysis. The risk of typos using comprehensive datasets was counteracted by parallel extraction. For pragmatic reasons, some categories of reasons for exclusion of participants were broad and may cover more subgroups. Moreover, the categorisation was prone to a degree of arbitrariness. Limited by the data at hand, we reported the authors’ declaration of statistical strategy without evaluating whether the strategy was maintained. We did not investigate alternatives to CONSORT. Some trials used patient flow diagrams, without specifically stating the use of a CONSORT diagram, which is why we chose to include all trials with patient flow diagrams. We did not go into details on participant characteristics, generalisability and pre-randomisation exclusion criteria as these data were investigated in a parallel review [29]. Obviously, we were unable to assess recruitment and retention in trials without flow diagrams, which may be different from trials with adequate flow diagrams. Where other reviews on CONSORT adherence have included trials from leading journals or a specific annual range [22, 24, 34], we included all trials investigating postoperative pain management following THA or TKA regardless of journal or publication year to make the review generalisable to the total research base. Nevertheless, we did a post hoc regression analysis to address associations with important variables.

## Perspectives

Though improvements have been made, the general adherence to reporting guidelines is suboptimal [35]. Continuous endorsement of the CONSORT Statement by editors and mandatory guideline checklists for journal submission could be ways to improve this [5, 36]. An expansion of the CONSORT flow diagram could facilitate higher external and internal validity, by adding more details concerning the process of inclusion and clarify reasons for exclusion following randomisation, while keeping the diagram manageable [22, 26].

## Conclusion

Adherence to participant flow diagrams was 42% among published RCTs investigating postoperative pain management after THA and TKA with a significant increase over time. Recruitment and retention rates are high. Still, there is room for improvement in quantitative and qualitatively reporting of flow diagrams, to increase transparency of study details and facilitate better and easier quality assessment of trial results.

## Supplementary Information

**Additional file 1.** Search strategy.

**Additional file 2.** Included trials.

**Additional file 3.** Reasons for exclusion of trials.

**Additional file 4.** Subgroup analysis of recruitment and retention for trials with adequate participant flow diagrams (continental, interventional and procedural).

## Data Availability

The datasets are available from the corresponding author on reasonable request.
