# Supplementary material for: Adherence to participant flow diagrams in trials on postoperative pain management after total hip and knee arthroplasty: a methodological review
Source: Trials. 2021 Apr 14;22:280. doi: 10.1186/s13063-021-05233-5 (PMC8048275; doi:10.1186/s13063-021-05233-5)
Supplement: Supplementary file 1 — Additional file 1. Search strategy. [file 13063_2021_5233_MOESM1_ESM.pdf]

Appendix 1: Search strategy (January 7th, 2020)

Pubmed Search (advanced)

#1.

("Pain"[Mesh] OR "Acute Pain"[Mesh] OR "Pain Management"[Mesh] OR "Pain, Procedural"[Mesh] OR "Analgesia"[Mesh] OR "Analgesia, Patient-Controlled"[Mesh] OR "Analgesia, Epidural"[Mesh] OR "Anesthesia and Analgesia"[Mesh] OR "Anesthesia"[Mesh] OR "Anesthesia, Spinal"[Mesh] OR "Anesthesia, Local"[Mesh] OR "Anesthesia, Intravenous"[Mesh] OR "Anesthesia, Epidural"[Mesh] OR "Analgesics"[Mesh] OR "Anesthetics"[Mesh] OR Analgesia[Title/Abstract] OR Analgesia Patient-Controlled[Title/Abstract] OR Analgesia Epidural[Title/Abstract] OR Anesthesia and Analgesia[Title/Abstract] OR Anesthesia[Title/Abstract] OR Anesthesia Spinal[Title/Abstract] OR Anesthesia Local[Title/Abstract] OR Anesthesia Intravenous[Title/Abstract] OR Anesthesia Epidural[Title/Abstract] OR Analgesics[Title/Abstract] OR Anesthetics[Title/Abstract] OR Acute Pains[Title/Abstract] OR Pain Acute[Title/Abstract] OR Pains Acute[Title/Abstract] OR Management Pain[Title/Abstract] OR Managements Pain[Title/Abstract] OR Pain Managements[Title/Abstract] OR Analgesias[Title/Abstract] OR Analgesia Patient Controlled[Title/Abstract] OR Patient-Controlled Analgesia[Title/Abstract] OR Patient Controlled Analgesia[Title/Abstract] OR Epidural Analgesia[Title/Abstract] OR Assessment Pain[Title/Abstract] OR Assessments Pain[Title/Abstract] OR Pain Assessments[Title/Abstract] OR Pain Assessment[Title/Abstract] OR Anesthesias Spinal[Title/Abstract] OR Spinal Anesthesia[Title/Abstract] OR Spinal Anesthesias[Title/Abstract] OR Local Anesthesia[Title/Abstract] OR Anesthesia Infiltration[Title/Abstract] OR Infiltration Anesthesia[Title/Abstract] OR Anesthesias Intravenous[Title/Abstract] OR Intravenous Anesthesia[Title/Abstract] OR Intravenous Anesthesias[Title/Abstract] OR Anesthesia Peridural[Title/Abstract] OR Anesthesias Peridural[Title/Abstract] OR Peridural Anesthesia[Title/Abstract] OR Peridural Anesthesias[Title/Abstract] OR Anesthesia Extradural[Title/Abstract] OR Anesthesias Extradural[Title/Abstract] OR Extradural Anesthesia[Title/Abstract] OR Extradural Anesthesias[Title/Abstract] OR Epidural Anesthesia[Title/Abstract] OR Anesthesias Epidural[Title/Abstract] OR Epidural Anesthesias[Title/Abstract] OR Anesthesia Regional[Title/Abstract] OR Regional Anesthesia[Title/Abstract] OR Analgesic Drugs[Title/Abstract] OR Drugs Analgesic[Title/Abstract] OR Analgesic Agents[Title/Abstract] OR Agents Analgesic[Title/Abstract] OR Antinociceptive Agents[Title/Abstract] OR Anesthetic Drugs[Title/Abstract] OR Drugs Anesthetic[Title/Abstract] OR Anesthetic Agents[Title/Abstract] OR Agents Anesthetic[Title/Abstract] OR Pain Management[Title/Abstract] OR Acute Pain[Title/Abstract] OR Pain[Title/Abstract] OR Pain Postoperative[Title/Abstract] OR Postoperative Pain[Title/Abstract] OR Postoperative Pains[Title/Abstract] OR "Pain, Postoperative"[Mesh])

#2

("Arthroplasty, Replacement, Hip"[Mesh] OR "Arthroplasty, Replacement, Knee"[Mesh] OR Arthroplasty Replacement Hip[Title/Abstract] OR Arthroplasty Replacement Knee[Title/Abstract] OR Total Hip Replacement[Title/Abstract] OR Total Hip Replacements[Title/Abstract] OR Hip Replacements Total[Title/Abstract] OR Replacement Total Hip[Title/Abstract] OR Hip Replacement Total[Title/Abstract] OR Hip Replacement Arthroplasties[Title/Abstract] OR Arthroplasties Hip OR Total Hip Arthroplasty OR Replacement[Title/Abstract] OR Replacement Arthroplasty Hip[Title/Abstract] OR Replacement Arthroplasties Hip[Title/Abstract] OR Hip Replacement Arthroplasty[Title/Abstract] OR hip arthroplasty[Title/Abstract] OR hip replacement[Title/Abstract] OR THA[Title/Abstract] OR hip surgery[Title/Abstract] OR Prosthesis Implantations

Hip[Title/Abstract] OR Prosthesis Implantation Hip[Title/Abstract] OR Implantations Hip Prosthesis[Title/Abstract] OR Implantation Hip Prosthesis[Title/Abstract] OR Hip Prosthesis Implantations[Title/Abstract] OR Hip Prosthesis Implantation[Title/Abstract] OR Arthroplasty Hip Replacement[Title/Abstract] OR Arthroplasties Replacement Hip[Title/Abstract] OR Replacement Arthroplasty Knee[Title/Abstract] OR Arthroplasties Knee Replacement[Title/Abstract] OR Arthroplasty Knee[Title/Abstract] OR Knee Arthroplasty[Title/Abstract] OR Knee Replacement Total[Title/Abstract] OR Total Knee Replacement[Title/Abstract] OR Replacement Total Knee[Title/Abstract] OR Total Knee Arthroplasty[Title/Abstract] OR Arthroplasty Total Knee[Title/Abstract] OR Knee Arthroplasty Total[Title/Abstract] OR Replacement Arthroplasties Knee[Title/Abstract] OR Knee Replacement Arthroplasty[Title/Abstract] OR Knee Replacement Arthroplasties[Title/Abstract] OR knee replacement[Title/Abstract] OR TKA[Title/Abstract] OR knee surgery[Title/Abstract] OR Arthroplasty Knee Replacement[Title/Abstract] OR Arthroplasties Replacement Knee[Title/Abstract])

#3

”randomized controlled trial”[ptyp] or ”controlled clinical trial”[PTyp]

#4

”randomized”[Title/Abstract] or ”randomised”[Title/Abstract] or ”randomly”[Title/Abstract] or ”random”[Title/Abstract] or ”controlled”[Title/Abstract] or ”trial”[Title/Abstract] or ”placebo”[Title/Abstract] or ”groups”[Title/Abstract] or ”clinical trial”[Title/Abstract]

#5

3 or 4

#6

1 and 2 and 5

8199 HITS

Embase Search (advanced)

#1.

Pain OR Acute Pain OR Pain Management OR Pain Procedural OR Analgesia OR Analgesia Patient-Controlled OR Analgesia Epidural OR Anesthesia and Analgesia OR Anesthesia OR Anesthesia Spinal OR Anesthesia Local OR Anesthesia Intravenous OR Anesthesia Epidural OR Analgesics OR Anesthetics OR Pain Acute OR Pains Acute OR Management Pain OR Managements Pain OR Pain Managements OR Analgesias OR Patient-Controlled Analgesia OR Patient Controlled Analgesia OR Epidural Analgesia OR Assessment Pain OR Assessments Pain OR Pain Assessments OR Pain Assessment OR Anesthesias Spinal OR Spinal Anesthesia OR Spinal Anesthesias OR Local Anesthesia OR Anesthesia Infiltration OR Infiltration Anesthesia OR Anesthesias Intravenous OR Intravenous Anesthesia OR Intravenous Anesthesias OR Anesthesia Peridural OR Anesthesias Peridural OR Peridural Anesthesia OR Peridural Anesthesias OR Anesthesia Extradural OR Anesthesias Extradural OR Extradural Anesthesia OR Extradural Anesthesias OR Epidural Anesthesia OR Anesthesias Epidural OR Epidural Anesthesias OR Anesthesia OR Regional Anesthesia OR Analgesic Drugs OR Drugs Analgesic OR Analgesic Agents OR Agents Analgesic OR Antinociceptive Agents OR Anesthetic Drugs OR Drugs Anesthetic OR Anesthetic Agents OR Agents Anesthetic OR Pain Postoperative OR Postoperative Pain OR Postoperative Pains (title, abstract, keyword)

#2

Arthroplasty Replacement Hip OR Arthroplasty Replacement Knee OR Total Hip Replacement OR Total Hip Replacements OR Hip Replacements Total OR Replacement Total Hip OR Hip Replacement Total OR Hip Replacement Arthroplasties OR Arthroplasties Hip Replacement OR Replacement Arthroplasty Hip OR Replacement Arthroplasties Hip OR Hip Replacement Arthroplasty OR Prosthesis Implantations Hip OR Prosthesis Implantation Hip OR Implantations Hip Prosthesis OR Implantation Hip Prosthesis OR Hip Prosthesis Implantations OR Hip Prosthesis Implantation OR Arthroplasty Hip Replacement OR Arthroplasties Replacement Hip OR Replacement Arthroplasty Knee OR Arthroplasties Knee Replacement OR Arthroplasty Knee OR Knee Arthroplasty OR Knee Replacement Total OR Total Knee Replacement OR Replacement Total Knee OR Total Knee Arthroplasty OR Arthroplasty Total Knee OR Knee Arthroplasty Total OR Replacement Arthroplasties Knee OR Knee Replacement Arthroplasty OR Knee Replacement Arthroplasties OR Arthroplasty Knee Replacement OR Arthroplasties Replacement Knee OR knee replacement OR TKA OR knee surgery OR hip arthroplasty OR hip replacement OR THA OR hip surgery OR Total Hip Arthroplasty (title, abstract, keyword)

#1 and #2

#3

Crossover-procedure or double-blind procedure or randomized controlled trial or single-blind procedure or random or factorial or crossover or cross over or placebo or assign or alloccat or volunteer (Text word (tw.))

#4

randomized or randomised or randomly or random or controlled or trial or placebo or groups or clinical trial (title, abstract, keyword)

#5

#3 or #4

#6

#1 and #2 and #5

3612 HITS

CENTRAL

#1.

Pain OR Acute Pain OR Pain Management OR Pain Procedural OR Analgesia OR Analgesia Patient-Controlled OR Analgesia Epidural OR Anesthesia and Analgesia OR Anesthesia OR Anesthesia Spinal OR Anesthesia Local OR Anesthesia Intravenous OR Anesthesia Epidural OR Analgesics OR Anesthetics OR Pain Acute OR Pains Acute OR Management Pain OR Managements Pain OR Pain Managements OR Analgesias OR Patient-Controlled Analgesia OR Patient Controlled Analgesia OR Epidural Analgesia OR Assessment Pain OR Assessments Pain OR Pain Assessments OR Pain Assessment OR Anesthesias Spinal OR Spinal Anesthesia OR Spinal Anesthesias OR Local Anesthesia OR Anesthesia Infiltration OR Infiltration Anesthesia OR Anesthesias Intravenous OR Intravenous Anesthesia OR Intravenous Anesthesias OR Anesthesia Peridural OR Anesthesias Peridural OR Peridural Anesthesia OR Peridural Anesthesias OR Anesthesia Extradural OR Anesthesias Extradural OR Extradural Anesthesia OR

Extradural Anesthesias OR Epidural Anesthesia OR Anesthesias Epidural OR Epidural Anesthesias OR Anesthesia OR Regional Anesthesia OR Analgesic Drugs OR Drugs Analgesic OR Analgesic Agents OR Agents Analgesic OR Antinociceptive Agents OR Anesthetic Drugs OR Drugs Anesthetic OR Anesthetic Agents OR Agents Anesthetic OR Pain Postoperative OR Postoperative Pain OR Postoperative Pains (title, abstract, keyword)

#2.

MeSH descriptor [Pain] explode all trees OR MeSH descriptor: [Analgesia] explode all trees

#3 .

#1 or #2

#4.

Arthroplasty Replacement Hip OR Arthroplasty Replacement Knee OR Total Hip Replacement OR Total Hip Replacements OR Hip Replacements Total OR Replacement Total Hip OR Hip Replacement Total OR Hip Replacement Arthroplasties OR Arthroplasties Hip Replacement OR Replacement Arthroplasty Hip OR Replacement Arthroplasties Hip OR Hip Replacement Arthroplasty OR Prosthesis Implantations Hip OR Prosthesis Implantation Hip OR Implantations Hip Prosthesis OR Implantation Hip Prosthesis OR Hip Prosthesis Implantations OR Hip Prosthesis Implantation OR Arthroplasty Hip Replacement OR Arthroplasties Replacement Hip OR Replacement Arthroplasty Knee OR Arthroplasties Knee Replacement OR Arthroplasty Knee OR Knee Arthroplasty OR Knee Replacement Total OR Total Knee Replacement OR Replacement Total Knee OR Total Knee Arthroplasty OR Arthroplasty Total Knee OR Knee Arthroplasty Total OR Replacement Arthroplasties Knee OR Knee Replacement Arthroplasty OR Knee Replacement Arthroplasties OR Arthroplasty Knee Replacement OR Arthroplasties Replacement Knee OR knee replacement OR TKA OR knee surgery OR hip arthroplasty OR hip replacement OR THA OR hip surgery OR Total Hip Arthroplasty OR hip prosthesis OR knee prosthesis (title, abstract, keyword)

#5

MeSH descriptor: [Arthroplasty, Replacement, Knee] explode all trees OR MeSH descriptor: [Arthroplasty, Replacement, Hip] explode all trees

#6.

#4 or #5

#7.

#3 AND #6

#7

limit #6 to clinical trials

8651 HITS

This search includes the following Mesh Terms: pain; analgesia; Arthroplasty, Replacement Knee; and Arthroplasty, Replacement Hip.
