# Supplementary material for: Adherence to participant flow diagrams in trials on postoperative pain management after total hip and knee arthroplasty: a methodological review
Source: Trials. 2021 Apr 14;22:280. doi: 10.1186/s13063-021-05233-5 (PMC8048275; doi:10.1186/s13063-021-05233-5)
Supplement: Supplementary file 2 — Additional file 2. Included trials. [file 13063_2021_5233_MOESM2_ESM.pdf]

Appendix 2: Included articles

ID	Publication year	First author	Title	Continent	Type of arthroplasty	Journal
1	1981	Reiz, S	Epidural morphine for postoperative pain relief	Europe	Hip	Acta Anaesth Scand
2	1983	O'Sullivan, G	A comparison of intramuscular and sublingual buprenorphine, intramuscular morphine and placebo as premedication	Europe	Hip	Anaesthesia
3	1983	Porter, EJ	Comparison of effects of intraoperative and postoperative methadone: acute tolerance to the postoperative dose?	Europe	Hip	Br J Anaesth
4	1984	Moore, RA	Controlled comparison of intrathecal cinchocaine with intrathecal cinchocaine and morphine. Clinical effects and plasma morphine concentrations	Europe	Hip	Br J Anaesth
5	1985	Lindgren	Diclofenac for pain after hip surgery	Europe	Hip	Acta Orthop Scand
6	1988	Buchanan, JM	Postoperative pain relief; a new approach: narcotics compared with non-steroidal antiinflammatory drugs	Europe	Hip	Ann Royal Coll Surg Eng
7	1988	Ferrante, F.M.	A statistical model for pain in patient-controlled analgesia and conventional intramuscular	North America	Knee	Anesth Analg
8	1988	Jacobsen, L	A dose-response study of intrathecal morphine: Efficacy, duration, optimal dose and side effects	North America	Hip and knee	Anesth Analg
9	1989	Fee, JPH	Analgesia after hip replacement surgery: Comparison of nalbuphine with morphine	Europe	Hip	Br J Anaesth
10	1989	Jacobson, L	Intrathecaldiamorphine: a dose- response study	Europe	Knee	Ann Royal Coll Surg Eng
11	1989	Nielsen, PT	Less pain with epidural morphine after knee arthroplasty	Europe	Knee	Acta Orthop Scand
12	1989	Reay, BA	low-dose intrathecal diamorphine analgesia following major ortopaedic surgery	Europe	Hip and knee	Br J Anaesth
13	1989	Serpell, MG	Comparison of piroxicam with placebo in the management of pain after total hip replacement	Europe	Hip	Br J Anaesth
14	1990	Segstro, R	The efficacy of indomethacin as a postoperative analgesic following total hip arthroplasty	North America	Hip	Can J Anaesth
15	1991	Carabine, UA	Extradural clonidine infusions for analgesia after total hip replacement	Europe	Hip	Br J Anaesth
16	1991	Misra, U	Plasma concentrations of bupivacaine following combined sciatic and femoral 3 in 1 nerve blocks in open knee surgery	Europe	Knee	Br J Anaesth
17	1991	Robinson, SL	Morphine compared with diamorphine	Europe	Hip	Anaesthesia
18	1991	Serpell, MG	Comparison of lumbar plexus block versus conventional opioid analgesia after total knee replacement	Europe	Knee	Anaesthesia
19	1991	Badner, Neal H	Low-Dose Bupivacaine Does Not Improve Postoperative Epidural Fentanyl Analgesia in Orthopedic Patients	North America	Knee	Anesth Analg
20	1991	Lytle, SA	Combining epidural fentanyl and lidocaine for postoperative pain	North America	Hip and knee	J Am Osteop Ass
21	1991	Segstro, R	Indomethacin as a postoperative analgesic for total hip arthroplasty	North America	Hip	Can J Anaesth
22	1991	Weller, R	Comparison of epidural and patient-controlled intravenous morphine following joint replacement surgery	North America	Hip and knee	Can J Anaesth
23	1992	Boeckstyns, Me	Piroxicam spares buprenorphine after total joint repl	Europe	Hip and knee	Acta Orthop Scand
24	1992	Edwards, ND	Continuous Low-Dose 3-in-1 Nerve Blockade for Postoperative Pain Relief After Total Knee Replacement	Europe	Knee	Anesth Analg
25	1992	Laitinen, J	Intravenous diclofenac coupled with PCA fentanyl for pain relief after total hip replacement	Europe	Hip	Anesthesiology
26	1992	Uhrbrand, B	Perioperative analgesia by 3-in-one block in total hip arthroplasty prospective randomized blind study	Europe	Hip	Acta Orthop. Belg
27	1993	Fogarty, DJ	Comparison of the analgesic effects of intrathecal clonidine and intrathecal morphine after spinal anaesthesia in patients undergoing total hip replacement	Europe	Hip	Br J Anaesth
28	1993	Holmstrom, B	Combined spinal epidural block versus spinal and epidural block for orthopaedic surgery	Europe	Hip and knee	Can J Anaesth
29	1993	Kandler, D	Analgesic action of metoclopramide in prosthetic hip surgery	Europe	Hip	Acta Anaesth Scand
30	1993	Ashburn, M.A	Oral transmucosal fentanylcitrate for treatment of postoperative pain	North America	Hip and knee	Anesth Analg
31	1993	McCormack, JP	A comparison of regularly dosed oral morphine and ondemand intramuscular morphine in the treatment of postsurgical	North America	Hip	Can J Anaesth
32	1994	Dahl, Jb	Immediate and prolonged effects of pre- versus postOP epidural	Europe	Knee	Acta Anaesth Scand
33	1994	Grace, D	Co-administration of pethidine and clonidine: a spinal anaesthetic technique for total hip replacement	Europe	Hip	Br J Anaesth
34	1994	Hommeril, Jl	Ketoprofen for pain after hip and knee arthroplasty	Europe	Hip and knee	Br J Anaesth
35	1994	Møiniche, S	The effect of balanced analgesia on early convalescence after major orthopaedic surgery	Europe	Hip and knee	Acta Anaesth Scand
36	1994	Wilder-Schmidt, CH	Epidural droperidol reduces the side effects and duration of analgesia of epidural sufentanil	Europe	Hip and knee	Anesth Analg
37	1995	Dahl, V	Prophylactic oral ibuprofen or ibuprofen-codeine versus placebo for postoperative pain after primary hip arthroplasty	Europe	Hip	Acta Anaesth Scand
38	1995	Fletcher	Influence of timing on the analgesic effect of intravenous ketorolac after orthopedic surgery	Europe	Hip	Pain
39	1995	Fogarty, Dj	Intramuscular ketorolac following total hip replacement with spina anaesthesia	Europe	Hip	Acta Anaesth Scand
40	1995	Fogarty, DJ	Postoperative analgesia following total hip replacement: a comparison of intrathecal morphine and diamorphine	Europe	Hip	J R Soc Med
41	1995	Grace, D	Ineffective analgesia after extradural tramadol hydrochloride in patients	Europe	Knee	Anaesthesia
42	1995	Grace, D	Postoperative Analgesia After Co-administration of Clonidine and Morphine by the Intrathecal Route in Patients Undergoing Hip Replacement	Europe	Hip	Anesth Analg
43	1995	Klimscha, W	Hemodynamic and analgesic effects of clonidine added repetitively to continuous epidural and spinal blocks	Europe	Hip	Anesth Analg
44	1995	Stubhaug	Lack of analgesic effect of 50 and 100 mg oral tramadol after orthopaedic surgery: a randomized, double-blind, placeboand standard active drug comparison	Europe	Hip	Pain
45	1996	Grace, D	A comparison of intrathecal morphine-6-glucuronide and intrathecal morphine sulfate as analgesics for total hip	Europe	Hip	Anesth Analg
46	1996	Hendolin, H	Does morphine premedication influence the pain and consumption of postoperative analgesics after total knee	Europe	Knee	Acta Anaesth Scand
47	1996	Kostamovaara, PA	Intravenous ketoprofen for pain relief after total hip or knee replacement	Europe	Hip	Acta Anaesth Scand
48	1996	Niemi, L	Evaluation of the usefulness of intrathecal bupivacaine infusion for analgesia after hip and knee arthroplasty	Europe	Hip and knee	Br J Anaesth
49	1996	Tarradell, R	Respiratory and analgesic effects of meperidine and tramadol in patients undergoing orthopedic surgery	Europe	Hip and knee	Met Find Exp Clin Pharm
50	1996	Badner, NH	Intra-Articular Injection of Bupivacaine in Knee-Replacement Operations	North America	Knee	J Bone Joint Surg Inc
51	1996	Herrick, Ia	Postoperative cognitive impairment in elderly. Choice of patient-controlled	North America	Hip and knee	Anaesthesia
52	1996	Hirst, GC	Femoral Nerve Block, Single injection versus continuous infusion for total knee arthroplasty	North America	Knee	Reg Anesth
53	1997	Wong, CS	Pre-emptive analgesia with ketamine, morphine and epidural lidocaine prior to total knee replacement	Asia	Knee	Can J Anaesth
54	1997	Bachmann, M	Intrathecal infusion of bupivacaine with or without morphine	Europe	Hip and knee	Br J Anaesth
55	1997	McSwiney, M	Intravenous regional analgesia using morphine. The effect on postoperative pain following total knee arthroplasty	Europe	Knee	Acta Anaesth Scand
56	1997	Rundshagen, I	Continuous spinal anaesthesia	Europe	Hip	Reg Anesth
57	1997	Badner, NH	Addition of morphine to intra-articular bupivacaine does not improve analgesia following knee joint replacement	North America	Knee	Reg Anesth
58	1997	Mauerhan, DR	Intra-articular Morphine and/or Bupivacaine in the Management of Pain After Total Knee Arthroplasty	North America	Knee	J Arthroplasty
59	1998	Allen, Jg	Postoperative analgesia following total knee arthroplasty: a study comparing spinal anesthesia and combined sciatic femoral 3-in-1 block	Europe	Knee	Reg Anesth Pain Med
60	1998	Berti, Marco	Comparison between epidural infusion of fentanyl/bupivacaine and morphine/bupivacaine after orthopaedic surgery	Europe	Hip	Can J Anaesth
61	1998	Fournier, R	Postoperative analgesia with "3-in- 1" femoral nerve block after prosthetic hip surgery	Europe	Hip	CAN J ANAESTH
62	1998	Kostamovaara, PA	Ketorolac, diclofenac and ketoprofen are equally efficacious for pain relief after total hip replacement surgery	Europe	Hip	Br J Anaesth
63	1998	Muldoon, T	Comparison between extradural infusion of ropivacaine or bupivacaine for the prevention of postoperative pain after total knee arthroplasty	Europe	Knee	Br J Anaesth
64	1998	Peduto, VA	Efficacy of propacetamol in the treatment of postoperative pain	Europe	Hip	Acta Anaesth Scand
65	1998	Singelyn, F	Effects of Intravenous Patient-Controlled Analgesia withMorphine, Continuous Epidural Analgesia, and ContinuousThree-in-One Block on Postoperative Pain and KneeRehabilitation After Unilateral Total Knee Arthroplasty	Europe	Knee	Anesth Analg
66	1998	Vercauteran, MP	Postoperative intrathecal patient-controlled analgesia with bupivacaine, sufentanil or a mixture of both	Europe	Hip and knee	Anaesthesia
67	1998	Weir, PS	Double-blind comparison of extradural block with three bupivacaine–ketamine mixtures in knee arthroplasty	Europe	Knee	Br J Anaesth
68	1998	Allen, HW	Peripheral Nerve Blocks Improve Analgesia After Total Knee Replacement Surgery	North America	Knee	Anesth Analg
69	1998	Tsueda, K	Mood during epidural patient-controlled analgesia with morphine or fentanyl	North America	Hip and knee	Anesthesiology
70	1999	Pang, W	Comparison of patient-controlled analgesia (PCA) with tramadol or morphine	Asia	Hip and knee	Can J Anaesth
71	1999	Kampe, S	Postoperative Analgesia with No Motor Block by Continuous Epidural Infusion of Ropivacaine 0.1% and Sufentanil After Total Hip Replacement	Europe	Hip	Anesth Analg
72	1999	Klasen, JA	Intraarticular, epidural, and intravenous analgesia after total knee arthroplasty	Europe	Knee	Acta Anaesth Scand
73	1999	Milligan, K	The charactaristics of analgesic requirements following subarachnoid diamorphine in patients undergoing total hip	Europe	Hip	Reg Anesth
74	1999	Möllman, M	Continuous spinal anaesthesia or continuous epidural anaesthesia for post-operative pain control after hip replacement	Europe	Hip	Eur J Anaesthesiol
75	1999	Wulf, H	Ropivacaine epidural anesthesia and analgesia versus general anesthesia and intravenous patient-controlled analgesia with morphine in the perioperative management of hip replacement. Ropivacaine Hip Replacement Multicenter Study	Europe	Hip	Anesth Analg

ID	Publication year	First author	Title	Continent	Type of arthroplasty	Journal
76	1999	Ganapathy, S	Modified Continuous Femoral Three-in-One Block for Postoperative Pain After Total Knee Arthroplasty	North America	Knee	Anesth Analg
77	1999	Ritter, MA	Intra-articular morphine and/or bupivacaine after total knee replacement	North America	Knee	J Bone Joint surg (Br)
78	2000	Yeh, CC	Absence of the preemptive analgesic effect of dextromethorphan in total knee replacement under epidural anesthesia	Asia	Knee	Acta Anaesthesiologica Sinica
79	2000	Bourke, M	A Comparison of Regularly Administered Sustained Release Oral Morphine with Intramuscular Morphine for Control of Postoperative Pain	Europe	Hip	Anesth Analg
80	2000	Cole, PJ	Efficacy and respirtory effects of low-dose spinal morphine for postoperative analgesia following knee arthroplasty	Europe	Knee	Br J Anaesth
81	2000	Fernandez-Liesa, JI	Effect of intrathecal methadone on postoperative analgesia after total hip arthroplasty	Europe	Hip	Acute Pain
82	2000	Fournier, R	A Comparison of Intrathecal Analgesia with Fentanyl or Sufentanil After Total Hip Replacement	Europe	Hip	Anesth Analg
83	2000	Fournier, R	Onset and offset of intrathecal morphine versus nalbuphine for postoperative pain relief after total hip replacement	Europe	Hip	Acta Anaesth Scand
84	2000	Milligan, K	The efficacy and safety of epidural infusion of levobupivacain with and without clonidine for postoperative pain relief in patients undergoing total hip replacement	Europe	Hip	Anesth Analg
85	2000	Singelyn, FJ	Extended "three-in-one" block after total knee arthroplasty: continuous versus patient-controlled techniques	Europe	Knee	Anesth Analg
86	2000	Stevens, RD	Lumbar Plexus Block Reduces Pain and Blood Loss Associated with Total Hip Arthroplasty	Europe	Hip	Anesthesiology
87	2000	Ward, M	A comparison of patient-controlled analgesia administered by the intravenous or intranasal route during the early	Europe	Hip and knee	Anaesthesia
88	2001	Ng, HP	Intraoperative single-shot "3-in-1" femoral nerve block with ropivacaine 0.25%, ropivacaine 0.5% or bupivacaine 0.25% provides comparable 48-hr analgesia after unilateral total knee replacement	Asia	Knee	Can J Anaesth
89	2001	Tan, PH	Intrathecal bupivacaine with morphine or neostig- mine for postoperative analgesia after total knee replacement surgery	Asia	Knee	Can J Anaesth
90	2001	Bertini, L	Postoperative analgesia by combined continuous infusion and patient-controlled epidural analgesia (PCEA) following hip replacement: ropivacaine versus bupivacaine	Europe	Hip	Acta Anaesth Scand
91	2001	Kampe, S	Comparison of continuous epidural infusion of ropivacaine and sufentanil with intravenous patient-controlled analgesia after total hip replacement	Europe	Hip	Anaesthesia
92	2001	McNamee, DA	Total knee replacement: a comparison of ropivacaine and bupivacaine in combined femoral and sciatic block	Europe	Knee	Acta Anaesth Scand
93	2001	Silvasti, M	Patient-controlled epidural analgesia versus continuous epidural analgesia after total knee arthroplasty	Europe	Knee	Acta Anaesth Scand
94	2001	Singelyn, F	Extended Femoral Nerve Sheath Block AfterTotal Hip Arthroplasty: Continuous Versus Patient-Controlled	Europe	Hip	Anesth Analg
95	2001	Weber, A	Epinephrine Does Not Prolong the Analgesia of 20 mL Ropivacaine 0.5% or 0.2% in a Femoral Three-In-One Block	Europe	Knee	Anesth Analg
96	2001	Zhou, T	Propacetamol Versus Ketorolac for Treatment of Acute Postoperative Pain After Total Hip or Knee Replacement	North America	Hip and knee	Anesth Analg
97	2002	Adams, Ha	Postoperative pain management in orthopaedic patients: no differences in pain score, but improved stress control by epidural anaesthesia	Europe	Knee	Eur J Anaesthesiol
98	2002	Casati, A	Intraoperative Epidural Anesthesia and Postoperative Analgesia with Levobupivacaine for Major Orthopedic Surgery: A Double-Blind, Randomized Comparison of Racemic Bupivacaine and Ropivacaine	Europe	Hip	Journal of Clinical Anesthesia
99	2002	Fournier, R	Epinephrine and clonidine do not improve intrathecal sufentanil analgesia after total hip replacement	Europe	Hip	Br J Anaesth
100	2002	Iohom, G	Effect of perioperative administration of dexketoprofen on opioidrequirements and in ammatory response following elective hiparthroplasty	Europe	Hip	Br J Anaesth
101	2002	Riad, T	Intrathecal morphine compared with diamorphine for postoperative analgesia following unilateral knee arthroplasty	Europe	Knee	Acute Pain
102	2002	Souron, V	Intrathecal morphine provides better postoperative analgesia than psoas compartment block after primary hip arthroplasty	Europe	Hip	Can J Anaesth
103	2002	Bogoch, Er	Lumbar paravertebral nerve block in the management	North America	Hip and knee	J Arthroplasty
104	2002	Camu, F	Valdecoxib, a COX-2–Specific Inhibitor, Is an Efficacious, Opioid-Sparing Analgesic in Patients Undergoing Hip	North America	Hip	Am J Therap
105	2002	Wang, H	The Effect of Single-Injection Femoral Nerve Block on Rehabilitation and Length of Hospital Stay After Total Knee	North America	Knee	Reg Anesth Pain Med
106	2003	Bianconi, M	Pharmacokinetics andefficacy of ropivacaine continuous wound	Europe	Hip and knee	Br J Anaesth
107	2003	Hubbard, AC	Parecoxib sodium has opioid-sparing effects in patients undergoing total knee arthroplasty under spinal anaesthesia	Europe	Knee	Br J Anaesth
108	2003	Manoir, BD	Randomized prospective study of the analgesic effect of nefopam after orthopaedic surgery	Europe	Hip	Br J Anaesth
109	2003	Maurer, K	Continuous spinal anesthesia/analgesia vs. single-shot spinal anesthesia with patient-controlled analgesia for elective hip	Europe	Hip	Acta Anaesth Scand
110	2003	Murphy, PM	Optimizing the Dose of Intrathecal Morphine in Older Patients Undergoing Hip Arthroplasty	Europe	Hip	Anesth Analg
111	2003	Reiter, A	Preoperative oral administration of fast-release morphine sulfate reduces postoperative piritramide consumption	Europe	Hip and knee	Wien Klin Wochenschr
112	2003	Bugter, MLT	Prior ibuprofen exposure does not augment opioid drug potency or modify opioid requirements for pain inhibition in	North America	Hip	Reg Anesth Pain
113	2003	Buvanendran, A	Effects of perioperative administration of a selective cyclooxygenase 2 inh	North America	Knee	JAMA
114	2003	Keita, H	Comparison between patient-controlled analgesia and subcutaneous morphine in elderly patients after total hip	North America	Hip	Br J Anaesth
115	2003	Ratmell, JP	Intrathecal Morphine for Postoperative Analgesia: A Randomized, Controlled, Dose-Ranging Study After Hip and Knee Arthroplast	North America	Hip and knee	Anesth Analg
116	2004	Biboulet, P	Postoperative analgesia after total-hip arthroplasty: comparison of intravenous	Europe	Hip	Reg Anesth Pain Med
117	2004	Davies, Af	Epidural infusion or combined femoral and sciatic	Europe	Knee	Br J Anaesth
118	2004	Förster, JG	Small dose of clonidine mixed with low-dose ropivacaine and fentanyl for epidural analgesia after total knee arthroplasty	Europe	Knee	Br J Anaesth
119	2004	Gurlit, S	Continuous spinal analgesia or opioid-added continuous epidural analgesia for postoperative pain control after hip	Europe	Hip	Eur J Anaesthesiol
120	2004	Macalou,D	Postoperative Analgesia After Total Knee Replacement: The Effect of an Obturator Nerve Block Added to the Femoral 3-in-1 Nerve Block	Europe	Knee	Anesth Analg
121	2004	Strebel, S	Small-Dose Intrathecal Clonidine and Isobaric Bupivacaine for Orthopedic Surgery: A Dose-Response Study	Europe	Hip and knee	Anesth Analg
122	2004	Browne, C	Bupivacaine bolus injection versus placebo for pain management following total knee arthroplasty	North America	Knee	J Arthroplasty
123	2004	Kaloul, I	The posterior lumbar plexus (psoas compartment) block and the three-in-one femoral nerve block provide similar postoperative analgesia after total knee replacement	North America	Knee	Can J Anaesth
124	2004	Sites, BD	A single injection ultrasound-assisted femoral nerve block provides side effect-sparing analgesia when compared with intrathecal morphine in patients undergoing total knee arthroplasty	North America	Knee	Anesth Analg
125	2004	Szczukowski, MJ	Femoral Nerve Block for Total Knee Arthroplasty Patients	North America	Knee	J arthroplasty
126	2005	Pitimana-aree, S	An economic evaluation of bupivacaine plus fentanyl versus ropivacaine alone for patient-controlled epidural analgesia after total-knee replacement procedure: a double-blinded randomized study	Asia	Knee	Reg Anesth Pain Med
127	2005	Barrington, M.J	Continuous femoral nerve blockade or epidural analgesia after total knee replacement	Australasia	Knee	Reg Anesth
128	2005	Adam, F	Small-Dose Ketamine Infusion Improves PostoperativeAnalgesia and Rehabilitation After Total Knee Arthroplast	Europe	Knee	Anesth Analg
129	2005	Casati, A	Adding Clonidine to the Induction Bolus and PostoperativeInfusion During Continuous Femoral Nerve Block DelaysRecovery of Motor Function After Total Knee Arthroplast	Europe	Knee	Anesth Analg
130	2005	Dang, CP	The value of adding sciatic block to continuous femoral block for analgesia after total knee replacement	Europe	Knee	Reg Anesth Pain Med
131	2005	Dobrydnjov, I	Improved analgesia with clonidine when added to local anesthetic during combined spinal-epidural anesthesia for hip arthroplasty: a double-blind, randomized and placebo-controlled study	Europe	Hip	Acta Anaesth Scand
132	2005	Fournier, R	Intrathecal Sufentanil Is More Potent Than Intravenous for Postoperative Analgesia After Total-Hip Replacement	Europe	Hip	Reg Anesth Pain Med
133	2005	Klasen, J	Impact on postoperative pain of long-lasting pre-emptive epidural analgesia before total hip replacement: a prospective, randomised, double-blind study	Europe	Hip	Anaesthesia
134	2005	Mannion, S	Tramadol as adjunct to psoas compartment block with levobupivacaine 0.5%: a randomized double-blinded study	Europe	Hip and knee	Br J Anaesth
135	2005	Morin, AM	Postoperative Analgesia and Functional Recovery After Total-Knee Replacement: Comparison of a Continuous Posterior Lumbar Plexus (Psoas Compartment) Block, a Continuous Femoral Nerve Block, and the Combination of a Continuous Femoral and Sciatic Nerve Block	Europe	Knee	Reg Anesth Pain Med
136	2005	Watson, MW	Continuous Versus Single-Injection Lumbar Plexus Blocks: Comparison of the Effects on Morphine Use and Early Recovery After Total Knee Arthroplasty	Europe	Knee	Reg Anesth Pain Med
137	2005	Chan,V.W	The Post-operative analgesic efficacy	North America	Hip and knee	Acta Anaesth Scand
138	2005	Nechleba, J	Continuous Intra-Articular Infusion of Bupivacaine for Postoperative Pain Following Total Knee Arthroplasty	North America	Knee	J knee Surg
139	2005	Sinatra, RS	Efficacy and Safety of Single and Repeated Administration of 1 Gram Intravenous Acetaminophen Injection (Paracetamol) for Pain Management after Major Orthopedic Surgery	North America	Hip and knee	Anesthesiology
140	2005	Yadeau, JT	The Effects of Femoral Nerve Blockade in Conjunction with Epidural Analgesia After Total Knee Arthroplasty	North America	Knee	Anesth Analg
141	2006	Bunburaphong, P	Postoperative analgesia for total knee replacement: Comparing	Asia	Knee	J Med Assoc Thai

ID	Publication year	First author	Title	Continent	Type of arthroplasty	Journal
142	2006	Chu, C.P	Postoperative outcome in Chinese patients having pimary total knee	Asia	Knee	Hong Kong Med J
143	2006	Ozen, M	The effect of 3-in-1 femoral nerve block with ropivacaine 0.375 % on postoperative morphine consumption in elderly patients after total knee replacement surgery	Asia	Knee	Agri
144	2006	Seet, E	Effectiveness of 3-in-1 Continuous Femoral Block of Differing Concentrations Compared to Patient Controlled Intravenous Morphine for Post Total Knee Arthroplasty Analgesia and Knee Rehabilitation	Asia	Knee	Anaesth and intensive care
145	2006	Tugay, N	Single injection femoral nerve block effects on the independence level in functional activities in the early postoperative period in patients with total knee arthroplasty	Asia	Knee	Neurosciences
146	2006	Casey, G	Perioperative Nimodipine and Postoperative Analgesi	Europe	Knee	Anesth Analg
147	2006	Manoir, BD	Evaluation of the pharmacokinetic profile and analgesic efficacy of oral morphine after total hip arthroplasty	Europe	Hip	Eur J Anaesthesiol
148	2006	Zaric, D	A comparison of epidural analgesia with combined continuous femoral-sciatic nerve blocks after total knee replacement	Europe	Knee	Anesth Analg
149	2006	Zippel, H	Comparison of the efficacy and safety of intravenously administered dexketoprofen trometamol and ketoprofen in the management of pain after orthopaedic surgery: a multicentre, double-blind, randomised, parallel-group clinical trial	Europe	Hip and knee	Clin Drug Investig
150	2006	Beaulieu, P	The pharmacodynamics of ropivacaine and bupivacaine in combined sciatic…	North America	Knee	Anesth Analg
151	2006	Hartrick, CT	Evaluation of a Single-Dose, Extended-Release Epidural Morphine Formulation for Pain After Knee Arthroplasty	North America	Knee	J bone joint surg
152	2006	Hartrick, CT	Fentanyl Iontophoretic Transdermal System for Acute-Pain Management After Orthopedic Surgery: A Comparative Study With Morphine Intravenous Patient-Controlled Analgesia	North America	Hip	Reg Anesth Pain Med
153	2006	Long, WT	Postoperative pain management following total knee arthroplasty: a randomized comparison of continuous epidural versus femoral nerve infusion	North America	Knee	J Knee Surg
154	2006	Mistraletti, G	Comparison of analgesic methods for total knee arthroplasty: metabolic effect of exogenous glucose	North America	Knee	Reg Anesth Pain Med
155	2006	Vendittoli, PA	A Multimodal Analgesia Protocol for Total Knee Arthroplasty	North America	Knee	J Bone Joint Surg Inc
156	2007	Bilir, A	Epidural magnesium reduces postoperative analgesic requirement	Asia	Hip	Br J Anaesth
157	2007	Huang, YS	Epidural Clonidine for Postoperative Pain After Total Knee Arthroplasty: A Dose–Response Study	Asia	Knee	Anesth Analg
158	2007	Inan, N	Efficacy of lornoxicam in postoperative analgesia after total knee replacement surgery	Asia	Knee	Agri
159	2007	Kim, MK	Epidural naloxone reduces postoperative nausea and vomiting in patients receiving epidural sufentanil for postoperative	Asia	Knee	Br J Anaesth
160	2007	Ozalp, G	The analgesic efficacy of two different approaches to the lumbar plexus for patient-controlled analgesia after total knee	Asia	Knee	J Anesth
161	2007	Rajeev, S	Combined Continuous b3-In-1Q and Sciatic Nerve Blocks Provide Improved Postoperative Analgesia with No Correlation to Catheter Tip Location After Unilateral Total Knee Arthroplasty	Asia	Knee	J arthroplasty
162	2007	Stevens, M	A modified fascia iliaca compartment block has significant morphine-sparing effect after total hip arthroplasty	Australasia	Hip	Anaesth and intensive care
163	2007	Andersen, KV	Reduced hospital stay and narcotic consumption, and improved mobilization with local and intraarticular infiltration after hip arthroplasty	Europe	Hip	Acta Orthopaedica
164	2007	Andersen, LJ	Pospoperative analgesia in total hip arthroplasty	Europe	Hip	Acta Orthopaedica
165	2007	Green, Rj	Comparison of the relative analgisic efficacies of epidural or intramuscular diamorphine	Europe	Knee	Eur J Anaesthesiol
166	2007	Martinez,V	The Influence of Timing of Administration on the Analgesic Efficacy of Parecoxib in Orthopedic Surgery	Europe	Hip	Anesth Analg
167	2007	Meunier, A	Effects of celecoxib on blood loss, pain, and recovery of function after total knee replacement	Europe	Knee	Acta Orthopaedica
168	2007	Romberg, R	A Randomized, Double-blind, Placebo-controlled Pilot Study of IV Morphine-6-Glucuronide for Postoperative Pain Relief After Knee Replacement Surgery	Europe	Knee	Clin J Pain
169	2007	Sitsen, E	Postoperative epidural analgesia after total knee arthroplasty with sufentanil 1 microg/ml combined with ropivacaine 0.2%, ropivacaine 0.125%, or levobupivacaine 0.125%: a randomized, double-blind comparison	Europe	Knee	Reg Anesth Pain Med
170	2007	Stiller, CO	The addition of tramadol to morphine via patient-controlled analgesia does not lead to better post-operative pain relief after total knee arthroplasty	Europe	Knee	Acta Anaesth Scand
171	2007	Toftdahl, K	Comparison of peri- and intraarticular analgesia with femoral nerve block after total knee arthroplasty: a randomized	Europe	Knee	Acta Orthopaedica
172	2007	Good, RP	Effects of a Preoperative Femoral Nerve Block on Pain Management and Rehabilitation After Total Knee Arthroplasty	North America	Knee	Am J Orthop
173	2007	Kardash, K	Obturator Versus Femoral Nerve Block for Analgesia After Total Knee Arthroplasty	North America	Knee	Anesth Analg
174	2007	Parvataneni, Hk	Controlling pain after total hip and knee arthroplasty using a multimodal protocol with local periarticular injections: a prospective randomized study	North America	Hip and knee	J arthroplasty
175	2007	Siddiqui, Z	Continuous Lumbar Plexus Block Provides Improved Analgesia With Fewer Side Effects Compared With Systemic Opioids After Hip Arthroplasty: A Randomized Controlled Trial	North America	Hip	Reg Anesth Pain Med
176	2007	Zugliani, AF	Control of Postoperative Pain Following Total Knee Arthroplasty: Is It Necessary to Associate Sciatic Nerve Block to Femoral Nerve Block?	South America	Knee	Rev Bras Anestesiol
177	2008	Elmawgoud, AA	Effect of addition of magnesium sulphate and fentanyl to ropivacaine continuous femoral nerve block in patients undergoing elective total knee replacement	Africa	Knee	Journal of Medical Sciences
178	2008	Fu, P	Efficacy of intra-articular cocktail analgesic injection in total knee arthroplasty—A randomized controlled trial	Asia	Knee	Knee
179	2008	Huang, YM	Perioperative celecoxib administration for pain management after total knee arthroplasty – A randomized, controlled	Asia	Knee	BMC musculoskeletal dis
180	2008	Koroglu, S	Total kalça protezinde preoperatif 3-1 blo¤un postoperatif a¤r› ve tramadol tüketimi üzerine etkisi	Asia	Hip	Agri
181	2008	Kumar, M	Tramadol Added to Bupivacaine Does Not Prolong Analgesia of Continuous Psoas Compartment Block	Asia	Hip	Pain Practice
182	2008	Becchi, C	Opioid-free analgesia by continuous psoas compartment block after total hip arthroplasty. A randomized study	Europe	Hip	Eur J Anaesthesiol
183	2008	Campbell, A	Epidural vs. Lumbar plexus infusions following	Europe	Knee	Eur J Anaesthesiol
185	2008	Förster, JG	Epinephrine 4 g/mL Added to a Low-Dose Mixture of Ropivacaine and Fentanyl for Lumbar Epidural Analgesia After Total Knee Arthroplasty	Europe	Knee	Anesth Analg
184	2008	Frassanito, L	The efficacy of the psoas compartment block versus the intrathecal combination of morphine, fentanyl and bupivacaine for postoperative analgesia after primary hip arthroplasty: a randomized single-blinded study	Europe	Hip	Eur Rev Med Pharmacol Sci
186	2008	Heid, F	Postoperative analgesic efficacy of peripheral levobupivacaine and ropivacaine: A prospective	Europe	Knee	Anesth Analg
187	2008	Leeuw	The Efficacy of Levobupivacaine, Ropivacaine, and Bupivacaine for Combined Psoas Compartment– Sciatic Nerve Block in Patients Undergoing Total Hip Arthroplasty	Europe	Hip	Pain Practice
188	2008	Martin, F	Lack of Impact of Intravenous Lidocaine on Analgesia, Functional Recovery, and Nociceptive Pain Threshold after Total Hip Arthroplasty	Europe	Hip	Anesthesiology
189	2008	Mathiesen, O	Pregabalin and dexamethasone for postoperative pain control: a randomized controlled study in hip arthroplasty	Europe	Hip	Br J Anaesth
190	2008	Minville, V	Postoperative analgesia after total hip arthroplasty: patient-controlled analgesia versus transdermal fentanyl patch	Europe	Hip	J Clin Anesth
191	2008	Smet, I	Randomized controlled trial of patient-controlled epidural analgesia after orthopaedic surgery with sufentanil and ropivacaine 0.165% or levobupivacaine 0.125%	Europe	Hip and knee	Br J Anaesth
192	2008	Tveita, T	A controlled comparison between single doses of intravenous and intramuscular morphine with respect to analgesic effects and patient safety	Europe	Hip	Acta Anaesthesiol Scand.
193	2008	Dorr, L.D	Multimodal analgesia without parenteral narcotics	North America	Knee	J Arthroplasty
194	2008	Kardash, K	Single-Dose Dexamethasone Reduces Dynamic Pain After Total Hip Arthroplasty	North America	Hip	Anesth Analg
195	2009	Makharita, MY	A comparison between intrathecal 100 mug morphine and intrathecal 75 mug neostigmine added to bupivacaine in patients undergoing total hip arthroplasty	Africa	Hip	Egypt J Anaesth
196	2009	Orbach-Zinger, S	Transdermal nitroglycerin as an adjuvant to patient-controlled morphine analgesia after total knee arthroplasty	Asia	Knee	Pain Res Manage
197	2009	Park, CK	Optimizing the dose of intrathecal morphine when combined with continuous 3-in-1 nerve block after total knee	Asia	Knee	Korean J Anesthesiol
198	2009	Shum, CF	Continuous femoral nerve block in total knee arthroplasty: immediate and two-year outcomes	Asia	Knee	J arthroplasty
199	2009	Sundarathiti, P	A Comparison of Continuous Femoral Nerve Block (CFNB) and Continuous Epidural Infusion (CEI) in Postoperative Analgesia and Knee Rehabilitation after Total Knee Arthroplasty (TKA)	Asia	Knee	J Med Assoc Thai
200	2009	Perrin, SB	Intraoperative ketamine may influence persistent pain following knee arthroplasty under combined general and spinal anaesthesia: a pilot study	Australasia	Knee	Anaesth and intensive care
201	2009	Reeves, M	Continuous intra-articular infusion of ropivacaine after unilateral total knee arthroplasty	Australasia	Knee	Anaesth and intensive care
202	2009	Aveline, C	Postoperative analgesia and early rehabilitation after total knee replacement:A comparison of continuous low-dose intravenous ketamine versus nefopam	Europe	Knee	Eur J Pain
203	2009	Frassanito, L	Anaesthesia for total knee arthroplasty: efficacy of single-injection or continuous lumbarplexus associated with sciatic nerve blocks –A randomized controlled study	Europe	Knee	Eur Rev Med Pharmacol Sci
204	2009	Kadic, L	Continuous femoral nerve block after total knee arthroplasty?	Europe	Knee	Acta Anaesth Scand
205	2009	Smith, TW	Efficacy and safety of morphine-6-glucuronide (M6G) for postoperative pain relief: a randomized, double-blind study	Europe	Knee	European Journal of Pain
206	2009	Christensen, CP	Effect of Periarticular Corticosteroid Injections During Total Knee Arthroplasty	North America	Knee	J Bone Joint Surg Inc

ID	Publication year	First author	Title	Continent	Type of arthroplasty	Journal
207	2009	Clarke, H	Adding Gabapentin to a multimodal regimen does not reduce acute pain, opioid consumption or chronic pain after total hip arthroplasty	North America	Hip	Acta Anaesth Scand
208	2009	Clarke, HA	Gabapentin decreases morphine consumption and improves functional recovery following total knee arthroplasty	North America	Knee	Pain Res Man
209	2009	Hunt, KJ	Single-Injection Femoral and Sciatic Nerve Blocks for Pain Control After Total Knee Arthroplasty	North America	Knee	J arthroplasty
210	2009	Marino, J	Continuous Lumbar Plexus Block for Postoperative Pain Control After Total Hip Arthroplasty	North America	Hip	J Bone Joint Surg Inc
211	2009	Duarte, Lt	Effects of epidural analgesia and continuous lumbar plexus	South America	Hip	Rev Bras Anestesiol
212	2009	Duarte, LTD	Posterior Lumbar Ple0us Block in Postoperative Analgesia for Total Hip Arthroplasty. A Comparative Study between 0.5% Bupivacaine with Epinephrine and 0.5% Ropivacaine	South America	Hip	Rev Bras Anestesiol
213	2010	Bengisun, ZK	Intraarticular levobupivacaine or bupivacaine administration decreases pain scores and provides a better recovery after total knee arthroplasty	Asia	Knee	J Anesth
214	2010	Chen, D	Continuous intra-articular infusion of bupivacaine for post-operative pain relief after total hip arthroplasty: A randomized, placebo-controlled, double-blind study	Asia	Hip	Eur J Pain
215	2010	FU, PL	Efficacy of a Multimodal Analgesia Protocol in Total Knee Arthroplasty: a Randomized, Controlled Trial	Asia	Knee	J Int Med Res
216	2010	Gupta, S.	Addition of epidural clonidine enhances postoperative analgesia: A double-blind	Asia	Knee	Anesth Essays Res
217	2010	Ho, KY	Duloxetine reduces morphine requirements after knee replacement surgery	Asia	Knee	Br J Anaesth
218	2010	Horasanli, E	A comparison of epidural anesthesia and lumbar plexus-sciatic nerve blocks for knee surgery	Asia	Knee	Clinics
219	2010	Hwang, JY	I.V. infusion of magnesium sulphate during spinal anaesthesia improves postoperative analgesia	Asia	Hip	Br J Anaesth
220	2010	Ittichaikulthol, W	The post-operative analgesic efficacy of celecoxib compared with placebo and parecoxib	Asia	Hip and knee	J Med Assoc Thai
221	2010	Ong, JCA	Continuous in ltration of local anaesthetic following total knee arthroplasty	Asia	Knee	J Orth Surg
222	2010	McMeniman, TJ	Femoral nerve block vs fascia iliaca block for total knee arthroplasty postoperative pain control: a prospective, randomized controlled trial	Australasia	Knee	J Arthroplasty
223	2010	Andersen, L.Ø	Analgesic efficacy of intracapsular and intra-articular local anaesthesia for knee arthroplasty	Europe	Knee	Anaesthesia
224	2010	Andersen, KV	A randomized, controlled trial comparing local infiltration analgesia with epidural infusion for total knee arthroplasty	Europe	Knee	Acta Orthopaedica
225	2010	Busch,	The Efficacy of Periarticular Multimodal Drug Infiltration in Total Hip Arthroplasty	Europe	Hip	Clin Orthop Relat Res
226	2010	Essving, P	Reduced morphine consumption and pain intensity with local infiltration analgesia (LIA) following total knee	Europe	Knee	Acta Orthopaedica
227	2010	Frassanito, L	Post.operative analgesia following total knee arthroplasty: Comparison of low-	Europe	Knee	Eur Rev Med Pharmacol Sci
228	2010	Gombotz, H	Opiate sparing effect of fixed combination of diclophenac and orphenadrine after unilateral total hip arthroplasty: A double-blind, randomized, placebo-controlled, multi-centre clinical trial	Europe	Hip and knee	Wien Med Wochenschr
229	2010	Gomez-Cardero, P	Postoperative Analgesia in TKA Ropivacaine Continuous Intraarticular Infusion	Europe	Knee	Clin Orthop Relat Res
230	2010	Rasmussen, M	Multimodal analgesia with gabapentin, ketamine and dexamethasone in combination with paracetamol and ketorolac after hip arthroplasty: a preliminary study	Europe	Hip	Eur J Anaesthesiol
231	2010	Spreng, UJ	High-volume local infiltration analgesia combined with intravenous or local ketorolac+morphine compared with epidural analgesia after total knee arthroplasty	Europe	Knee	Br J Anaesth
232	2010	Carli, F	Analgesic nd functional outcome after	North America	Knee	Br J Anaesth
233	2010	Ilfeld, Bm	A Multicenter, randomized, triple-masked, placebo-controlled trial of the effect	North America	Knee	Pain
234	2010	Rosen, AS	A Randomized Controlled Trial of Intraarticular Ropivacaine for Pain Management Immediately Following Total Knee Arthroplasty	North America	Knee	Hospital for special Surgery/HSSJ
235	2010	Garcia, JBS	Analgesic Efficacy of the Intra-Articular Administration of High Doses of Morphine in Patients Undergoing Total Knee	South America	Knee	Rev Brasil Anest
236	2011	Anis, S	Lumbar plexus block as a method of postoperative analgesia after hip surgery	Africa	Hip	Egypt J Anaesth
237	2011	Lee, AR	Effect of Combined Single-Injection Femoral Nerve Block and Patient-Controlled Epidural Analgesia in Patients Undergoing Total Knee Replacement	Asia	Knee	Yonsei Med J
238	2011	Liu, W	Reduced Opioid Consumption and ImprovedEarly Rehabilitation with Local andIntraarticular Cocktail Analgesic Injection inTotal Hip Arthroplasty: A RandomizedControlled Clinical Trial	Asia	Hip	Pain Medicine
239	2011	Seah, VWT	Single-dose periarticular steroid in ltration for pain management in total knee arthroplasty: a prospective, double- blind, randomised controlled trial	Asia	Knee	Singapore Med J
240	2011	Zhang, S	Effect of Single-injection versus Continuous Local Infiltration Analgesia after Total Knee Arthroplasty: a Randomized, Double-blind, Placebo- controlled Study	Asia	Knee	J Int Med Res
241	2011	Affas, F	Pain control after total knee arthroplasty: a randomized trial comparing local infiltration anesthesia and continuous	Europe	Knee	Acta Orthopaedica
242	2011	Cappelleri, G	Does Continuous Sciatic Nerve Block Improve PostoperativeAnalgesia and Early Rehabilitation After TotalKnee Arthroplasty?	Europe	Knee	Reg Anesth Pain Med
243	2011	Essving, P	Local infiltration analgesia versus intrathecal morphine for postoperative	Europe	Knee	Anesth Analg
244	2011	Lunn, TH	Effect of high-dose preoperative methylprednisolone on pain and recovery after total knee arthroplasty: a randomized, placebo-controlled trial	Europe	Knee	Br J Anaesth
245	2011	Lunn, TH	Intraoperative Local Infiltration Analgesia for Early AnalgesiaAfter Total Hip Arthroplasty	Europe	Hip	Reg Anesth Pain Med
246	2011	Oberhofer, D	Low dose spinal morphine and intravenous diclofenac for postoperative analgesia after total hip and knee arthroplasty	Europe	Hip and knee	Periodicum Biol
247	2011	Rothwell, MP	Oral oxycodone offers equivalent analgesia to intravenous patient-controlled analgesia after total hip replacement: a randomized, single-centre, non-blinded, non-inferiority study	Europe	Hip	Br J Anaesth
248	2011	Specht, K	No evidence of a clinically important efect of adding local infusion analgesia administrated through a catheter in pain treatment after total hip arthroplasty	Europe	Hip	Acta Orthopaedica
249	2011	Wegener, YT	Value of Single-Injection or Continuous Sciatic Nerve Block in Addition to a Continuous Femoral Nerve Block in Patients Undergoing Total Knee Arthroplasty	Europe	Knee	Reg Anesth Pain Med
250	2011	Hartrick, CT	Capsaicin instillation for postoperative pain following total knee arthroplasty	North America	Knee	Clin Drug Investig
251	2011	Johnson, Cb	The use of continuous femoral nerve block versus extended release epidural morphine	North America	Knee	Orthopaedic Nursing
252	2012	Abrisham, SMJ	Reduced morphine consumption and pain severitywith transdermal fentanyl patches following total	Asia	Knee	Knee Surg Sports Traumatol Arthrosc
253	2012	Chan, M	Single-injection femoral nerve block lacks preemptive effect on postoperativepain and morphine consumption in total knee arthroplasty	Asia	Knee	Acta Anaesth Taiwanica
254	2012	Chinachoti, T	Periarticular infiltration of 0,25% bupivacaine on top of femoral nerve block and intratthecal morphine improves quality of pain control after total knee arthroplasty: A randomized double -blind placebo controlled clinical trial	Asia	Knee	J Med Assoc Thai
255	2012	Garg	Evaluation of single epidural bolus dose of magnesium as an adjuvant to epidural fentanyl for postoperative analgesia: A prospective, randomized, double-blind study	Asia	Hip	Saudi J Anaesth
256	2012	Jain and Jolly	Evaluation of efficacy of oral pregabalin in reducing postoperative pain in patients undergoing total knee arthroplasty	Asia	Knee	Indian J Orthop
257	2012	Jain, A	Analgesic efficacy of low-dose intrathecal neostigmine in combination with fentanyl and bupivacaine for total knee replacement surgery	Asia	Knee	J Anaesth Clin Pharm
258	2012	Koh, IJ	Does periarticular injection have additional pain relieving effects during contemporary multimodal pain control protocols for TKA?: A randomised, controlled study	Asia	Knee	Knee
259	2012	Koh, IJ	Does ramosetron reduce postoperative emesis and pain after TKA?	Asia	Knee	Clin Orthop Relat Res
260	2012	Lee, JJ	Effect of continuous psoas compartment block and intravenous patient controlled analgesia on postoperative pain control after total knee arthroplasty	Asia	Knee	Korean J Anesthesiol
261	2012	Yuenyongviwat, V	Periarticular Injection with Bupivacaine for Postoperative Pain Control in Total Knee Replacement: A Prospective Randomized Double-Blind Controlled Trial	Asia	Knee	Advances in Orthopedics
262	2012	Widmer, BJ	Is Femoral Nerve Block Necessary During Total Knee Arthroplasty?	Australasia	Knee	J Arthroplasty
263	2012	Aguirre, J	Continuous epicapsular ropivacaine 0.3% infusion after minimally invasive hip arthroplasty: a prospective, randomized, double-blinded, placebo-controlled study comparing continuous wound infusion with morphine patient-controlled	Europe	Hip	Anesth Analg
264	2012	Divella, M	Pain relief after total hip replacement: oral CR oxycodone plus IV paracetamol versus epidural levobupivacaine and sufentanil. A randomized controlled trial	Europe	Hip	Minerva anestesiologica
265	2012	Dobie, I	Periarticular Local Anesthesia does not Improve Pain or Mobility after THA	Europe	Hip	Clin Orthop Relat Res
266	2012	Jaeger, P	Effect of adductor-canal-blockade on established, severe post-operative pain after total knee arthroplasty: a randomised	Europe	Knee	Acta Anaesth Scand
267	2012	Jenstrup, MT	Effects of Adductor-Canal-Blockade on pain and ambulation after total knee arthroplasty: a randomized study	Europe	Knee	Acta Anaesth Scand
268	2012	Joppich, R	Analgesic Efficacy and Tolerability of Intravenous Morphine Versus Combined Intravenous Morphine and Oxycodone in a 2-Center, Randomized, Double-Blind, Pilot Trial of Patients With Moderate to Severe Pain After Total Hip	Europe	Hip	Clinical Therapeutics
269	2012	Lunn, TH	Effect of high-dose preoperative methylprednisolone on recovery after total hip arthroplasty: a randomized, double-blind, placebo-controlled trial	Europe	Hip	Br J Anaesth
270	2012	Mahadevan, D	Combined femoral and sciatic nerve block vs combined femoral and periarticular infiltration in total knee arthroplasty: a randomized controlled trial	Europe	Knee	J arthroplasty
271	2012	McNamee, DA	Post-operative analgesia following total knee replacement: an evaluation of the addition of an obturator nerve block to combined femoral and sciatic nerve block	Europe	Knee	Acta Anaesth Scand

ID	Publication year	First author	Title	Continent	Type of arthroplasty	Journal
272	2012	Murphy, TP	Can a Periarticular Levobupivacaine Injection Reduce Postoperative Opiate Consumption During Primary Hip	Europe	Hip	Clin Orthop Relat Res
273	2012	Onutu, AH	Wound infiltration with 1% pethidine provides anopioid-sparing effect after uncemented total hip arthroplasty:a prospective randomized study	Europe	Hip	J Român de Anestezie Ter int
274	2012	Rikalainen, SR	Local infiltration analgesia with levobupivacaine compared with intrathecal morphine in total hip arthroplasty patients	Europe	Hip	Acta Anaesth Scand
275	2012	Bramlett, K	A randomized, double-blind, dose-ranging study comparing wound infiltration of depofoam	North America	Knee	Knee
276	2012	Shanthanna, H	Comparative study of ultrasound-guided continuous femoral nerve blockade with continuous epidural analgesia for pain relief following total knee replacement	North America	Knee	Indian Journal of Anaesthesia
277	2012	Sinha, SK	Femoral nerve block with selective tibial nerve block provides effective analgesia without foot drop after total knee arthroplasty: a prospective, randomized, observer-blinded study	North America	Knee	Anesth Analg
278	2012	Xie, Z	Three-in-One Nerve Block With Different Concentrations of Bupivacaine in Total Knee Arthroplasty	North America	Knee	J Arthroplasty
279	2012	Sobrinho, G	Analgesic Efficacy of the Intra-articular Administration of S(+)- Ketamine in Patients Undergoing Total Knee	South America	Knee	Rev Bras Anestesiol.
280	2013	Samir, EM	Intrathecal vs intravenous magnesium as anadjuvant to bupivacaine spinal anesthesia for totalhip arthroplasty	Africa	Hip	Egypt J Anaesth
281	2013	Bayazit, Eg	Effect of epidural levobupivacaine and levobupivacaine with fentanyl	Asia	Knee	Int J Clin Pharm Ther
282	2013	Cengiz, P	Intraoperative Low-Dose Ketamine Infusion Reduces AcutePostoperative Pain Following Total Knee Replacement Surgery: A Prospective, Randomized Double-Blind Placebo-Controlled Tria	Asia	Knee	J College Phys Surg Pakistan
283	2013	Daabiss, MA	Evaluation of the effect of magnesium vs. midazolam as adjunct to epidural bupivacaine in patients undergoing total knee replacement	Asia	Knee	Brit J Med Pract
284	2013	Gong, L	Effects of Combined Application of Muscle Relaxants and Celecoxib Administration After Total Knee Arthroplasty (TKA) on Early Recovery: A Randomized, Double-Blind, Controlled Study	Asia	Knee	J Arthroplasty
285	2013	Ikeuchi, M	Effects of dexamethasone on local infiltration analgesia in total knee arthroplasty: a randomized controlled trial	Asia	Knee	Knee Surg Sports Traumatol Arthrosc
286	2013	Ikeuchi, M	Local infusion analgesia using intra-articular double lumen catheter after otal knee	Asia	Knee	Knee Surg Sports Traumatol Arthrosc
287	2013	Koh, IJ	Preemptive Low-dose Dexamethasone Reduces Postoperative Emesis and Pain After TKA: A Randomized Controlled	Asia	Knee	Clin Orthop Relat Res
288	2013	Misiran, KB	The effectiveness of patient-controlled epidural analgesia with ropivacaine 0.165% with fentanyl 2.0 miroc g/ml or levobupivacaine 0.125% with fentanyl 2.0 micro g/ml as a method of postoperative analgesia after major orthopaedic	Asia	Hip and knee	M E J Anesth
289	2013	Nakai, T	Controlling pain after total knee arthroplasty using a multimodal protocol with local periarticular injections	Asia	Knee	Journal of orthopaedics
290	2013	Niruthisard, S	Preoperative pregabalin and/or celecoxib for pain management after total knee arthroplasty under intrathecal morphine: a randomized controlled trial	Asia	Knee	Asian Biomedicine
291	2013	Sakai, N	Continuous femoral versus epidural block for attainment of 120 degrees knee fle0ion after total knee arthroplasty: a randomized controlled trial	Asia	Knee	J arthroplasty
292	2013	Tammachote, N	Is Pain After TKA Better with Periarticular Injection or Intrathecal Morphine?	Asia	Knee	Clin Orthop Relat Res
293	2013	Yue, DB	Ef cacy of multimodal cocktail periarticular injection with or without steroid in total knee arthroplasty	Asia	Knee	Chin Med J
294	2013	Zhu, Y	Effect of perioperative parecoxib on postoperative pain and local inflammation factors PGE2 and IL-6 for total knee arthroplasty: a randomized, double-blind, placebo-controlled study	Asia	Knee	Eur J Surg Traumatol
295	2013	Chan, E	Comparing the Analgesia Effects of Single-injection and Continuous Femoral NerveBlocks with Patient Controlled Analgesia after Total Knee Arthroplasty	Australasia	Knee	Journal of Arthroplasty
296	2013	Chia, Sk	Peri-articular teroid injection in total knee	Australasia	Knee	J Arthroplasty
297	2013	Andersen, HL	Continuous Saphenous Nerve Block as Supplement toSingle-Dose Local Infiltration Analgesia for PostoperativePain Management After Total Knee Arthroplasty	Europe	Knee	Reg Anesth Pain Med
298	2013	Andersen, KV	Local infiltration analgesia for total knee arthroplasty:should ketorolac be added?	Europe	Knee	Br J Anaesth
299	2013	Ashraf, A	Pain control after primary total knee replacement	Europe	Knee	Knee
300	2013	Chaumeron, A	Periarticular injection in knee arthroplasty	Europe	Knee	Clin Orthop Relat Res
301	2013	Dauri, M	The comparing of ultrasound-guided techniques: sciati	Europe	Knee	Open Anesth J
302	2013	Harsten, A.	General anaesthesia with multimodal principles versus intrathecal analgesia	Europe	Knee	J Clin Med Res
303	2013	Jæger, P	Adductor canal block versus femoral nerve block for analgesia after total knee	Europe	Knee	Reg Anesth Pain Med
304	2013	Kuchalik, J	Postoperative pain relief after total hip arthroplasty: a randomized, double-blind comparison between intrathecal morphine and local infiltration analgesia	Europe	Hip	Br J Anaesth
305	2013	Pandazi, A	Periarticular infiltration for pain relief after total hip arthroplasty: a comparison with epidural and PCA analgesia	Europe	Hip	Arch Orthop Trauma Surg
306	2013	Rawal, N	Evaluation of etoricoxib in patients undergoing total knee replacement surgery in a double-blind, randomized controlled	Europe	Knee	BMC musculoskeletal disorders
307	2013	Theodosiadis, P	Ropivacaine versus bupivacaine for 3-in-1 block during total knee arthroplasty	Europe	Knee	Journal of Orthopaedic Surgery
308	2013	Backes, J	Dexamethasonereduces length of hospitalization and improves postOP pan	North America	Hip and knee	J Arthroplasty
309	2013	Carmichael, Nm	An intensive perioperative regimen	North America	Hip	Pain Res Man
310	2013	Hillegass, Mg	The efficacy of automated intermittent boluses for continuous femoral nerve block: a prospective	North America	Knee	J Clin Anesth
311	2013	Kelley, TC	Efficacy of Multimodal Perioperative Analgesia Protocol With Periarticular Medication Injection in Total Knee Arthroplasty: A Randomized, Double-Blinded Study	North America	Knee	J arthroplasty
312	2013	Nader, A	A dose-ranging study of 0.5% bupivacaine or ropivacaine on the success and duration of the ultrasound-guided, nerve-stimulator-assisted sciatic nerve block: a double-blind, randomized clinical trial	North America	Knee	Reg Anesth Pain Med
313	2013	Pang, W	Metoclopramide improves the quality of tramadol PCA indistinguishable to morphine PCA: a prospective, randomized, double blind clinical comparison	North America	Knee	Pain Medicine
314	2013	Paul, JE	Gabapentin does not improve multimodal analgesia outcomes for total knee arthroplasty: a randomized controlled trial	North America	Knee	Can J Anaesth
315	2013	Shariat, A	Fascia lliaca Block for Analgesia After Hip Arthroplasty	North America	Hip	Reg Anesth Pain Med
316	2013	Solovyova, O	Local Infiltration Analgesia Followed by Continuous Infusion of Local Anesthetic Solution forTotal Hip	North America	Hip	J Bone Joint Surg Inc
317	2013	Williams, D	Continuous Infusion of Bupivacaine Following Total Knee Arthroplasty: A Randomized Control Trial Pilot Study	North America	Knee	J Arthroplasty
318	2013	YaDeau, JT	Analgesia after total knee replacement: Local infiltration versus epidural combined with a femoral nerve blockade: A prospective, randomised pragmatic trial	North America	Knee	Bone Joint J
319	2013	Lauretti, GR	Intrathecal ketorolac enhances intrathecal morphine analgesia following total knee arthroplasty	South America	Knee	J Anaesthesiol Clin Pharm
320	2014	Binici, Be	A comparison of epidural analgesia and local infiltration	Asia	Knee	Acta Orthop Traumatol Turc
321	2014	Chen, D	Intra-Articular Bupivacaine Reduces Postoperative Pain and Meperidine Use After Total Hip Arthroplasty: A Randomized, Double-Blind Study	Asia	Hip	J Arthroplasty
322	2014	Erol, IB	Effect of Ketamine on the Quality of Anesthesia and Postoperative Analgesia in Epidural Anesthesia	Asia	Hip	J Exp Clin Med
323	2014	Gi, E	Effects of local infiltration analgesia for posterior knee pain after total knee	Asia	Knee	J Anesth
324	2014	Kunopart, M	Effects of single shot femoral nerve block combined with intrathecal morphine for postoperative analgesia: A randomized, controlled, dose-ranging study after total knee arthroplasty	Asia	Knee	J Med Assoc Thai
325	2014	Leownorasate, M	Post-op pain and blood loss in total knee arthroplasty: An RCT using periarticular injection with diclofenac-based	Asia	Knee	J Med Assoc Thai
326	2014	Long, G	Effects of adenosine triphosphate (ATP) on early recovery after total knee arthroplasty (TKA): a randomized, double-blind, controlled study	Asia	Knee	J arthroplasty
327	2014	Moghtadaei, M	Pain management for total knee arthroplasty: single-injection femoral nerve block versus local infiltration analgesia	Asia	Knee	Iran Red Cres Med J
328	2014	Peng, L	Continuous femoral nerve block versus intravenous patient controlled analgesia for knee mobility and long-term pain in patients receiving total knee replacement: a randomized controlled trial	Asia	Knee	Evidence-Based Compl Alt Med
329	2014	Sahin, L	Ultrasound-guided single-injection femoral nerve block provides effective analgesia after total knee arthroplasty up to 48	Asia	Knee	Agri
330	2014	Sathitkarnmanee, T	Transdermal fentanyl patch for postoperative analgesia in total knee arthroplasty: a randomized double-blind controlled	Asia	Knee	J Pain Res
331	2014	Sato, K	Continuous Versus Single-Injection Sciatic Nerve Block Added to Continuous Femoral Nerve Block for Analgesia After Total Knee Arthroplasty A Prospective, Randomized, Double-Blind Study	Asia	Knee	Reg Anesth Pain Med
332	2014	Shah, NA	Is continuous adductor canal block better than continuous femoral nerve block after total knee arthroplasty? Effect on ambulation ability, early functional recovery and pain control: a randomized controlled trial	Asia	Hip	J arthroplasty
333	2014	Sivrikoz, N	Perioperative dexketoprofen or lornoxicam administration for pain management after major orthopedic surgery: a randomized, controlled study	Asia	Hip and knee	Agri
334	2014	Tsukada, S	Postoperative Epidural Analgesia Compared with Intraoperative Periarticular Injection for Pain Control Following Total Knee Arthroplasty Under Spinal Anesthesia	Asia	Knee	J Bone Joint Surg Inc
335	2014	Uesugi, K	Comparison of peripheral nerve block with periarticular injection analgesia after total knee arthroplasty: A randomized, controlled study	Asia	Knee	Knee
336	2014	Wu, JWS	Elective unilateral total knee replacement using continuous femoral nerve blockade versus conventional patient-controlled analgesia: perioperative patient management based on a multidisciplinary pathway	Asia	Knee	Hong Kong Med J
337	2014	Yavuz, N	The effect of intraarticular levobupivacaine and bupivacaine injection on the postoperative pain management in total knee artroplastic surgery	Asia	Knee	Pak J Med Sci

ID	Publication year	First author	Title	Continent	Type of arthroplasty	Journal
338	2014	Zhang, W	Ultrasound-guided continuous adductor canal block for analgesia after total knee replacement	Asia	Knee	Chin Med J
339	2014	Anastase, D	Effects of regional anaesthesia techniques on patients' satisfaction after total knee arthroplasty	Europe	Knee	J Român de Anestezie Ter int
340	2014	Jagla, C	Peripheral opioid receptor blockade increases postoperative morphine demands -- a randomized…	Europe	Knee	Pain
341	2014	Kosel, J	Buprenorphine Added to Bupivacaine Prolongs Femoral Nerve Block Duration and Improves Analgesia in Patients Undergoing Primary Total Knee Arthroplasty—A Randomised Prospective Double-Blind Study	Europe	Knee	J arthroplasty
342	2014	Martinez,V	The analgesic efficiency of combined pregabalin and ketamine for total hip arthroplasty: a randomised, double-blind,	Europe	Hip	Anaesthesia
343	2014	Niemeläinen, M	Single periarticular local in ltration analgesia reduces opiate consumption until 48 hours after total knee arthroplasty	Europe	Knee	Acta Orthopaedica
344	2014	Oreskovic, Z	Treatment of postoperative pain after total hip arthroplasty: comparison between metamizol and paracetamol as adjunctive to opioid analgesics-prospective, double-blind, randomised study	Europe	Hip	Arch Orthop Trauma Surg
345	2014	Wiesmann,	Supplemental singleshot femoral nerve block for total hip arthroplasty	Europe	Hip	Minerva anestesiologica
346	2014	Zoric, L	Single-shot intraoperative local anaesthetic infiltration does not reduce morphine consumption after total hip arthroplasty: a double-blinded placebo-controlled randomized study	Europe	Hip	Br J Anaesth
347	2014	Abdallah, FW	The Analgesic Effects of Proximal, Distal, or No Sciatic Nerve Block on Posterior Knee Pain after Total Knee Arthroplasty	North America	Knee	Anesthesiology
348	2014	Albrecht, Eric	Single-injection or Continuous Femoral Nerve Block for Total Knee Arthroplasty?	North America	Knee	Clin Orthop Relat Res
349	2014	Clarke, HA	Perioperative gabapentin reduces 24 h opioid consumptionand improves in-hospital rehabilitation but not post-dischargeoutcomes after total knee arthroplasty with peripheralnerve block	North America	Knee	Br J Anaesth
350	2014	Hanson, NA	Continuous Ultrasound-Guided Adductor Canal Block for Total Knee Arthroplasty: A Randomized, Double-Blind Trial	North America	Knee	Anesth Analg
351	2014	Kim, DH	Adductor canal block versus femoral nerve block for total knee arthroplasty: a prospective, randomized, controlled trial	North America	Knee	Anesthesiology
352	2014	Lamplot, JD	Multimodal pain management in total knee arthroplasty: a prospective randomized controlled trial	North America	Knee	J Arthroplasty
353	2014	Reinhardt, KR	Intraarticular analgesia versus epidural plus femoral nerve block after TKA: a randomized, double-blind trial	North America	Knee	Clin Orthop Relat Res
354	2014	Safa, B	Comparing the Effects of Single Shot Sciatic Nerve Block Versus Posterior Capsule Local Anesthetic Infiltration on Analgesia and Functional Outcome After Total Knee Arthroplasty	North America	Knee	J arthroplasty
355	2014	Spangehl, MJ	The Chitranjan Ranawat Award: periarticular injections and femoral & sciatic blocks provide similar pain relief after TKA: a randomized clinical trial	North America	Knee	Clin Orthop Relat Res
356	2014	Surdam, J	The Use of Exparel (Liposomal Bupivacaine) to Manage Postoperative Pain in Unilateral Total Knee Arthroplasty	North America	Knee	J arthroplasty
357	2014	Wardhan, R	Is L2 Paravertebral Block Comparable to Lumbar Plexus Block for Postoperative Analgesia After Total Hip	North America	Hip	Clin Orthop Relat Res
358	2014	Neto, JO	Methadone patient-controlled analgesia for postoperative pain: a randomized, controlled, double-blind study	South America	Hip	J Anesth
359	2015	Al-Zahrani, T	Randomized clinical trial of continuous femoral nerve block combined with sciatic nerve block versus epidural analgesia for unilateral total knee arthroplasty	Asia	Knee	J Arthroplasty
360	2015	Chen, JIA	Efficacy of celecoxib for acute pain management following total hip arthroplasty in elderly patients: A prospective, randomized, placebo-control trial	Asia	Hip	Exp Ther Med
361	2015	Kasture, S	Epidural versus intra-articular infusion analgesia following total knee replacement	Asia	Knee	J Orthop Surg (Hong Kong)
362	2015	Kim, TW	Which analgesic mixture is appropriate for periarticular injection after total knee arthroplasty? Prospective, randomized, double-blind study	Asia	Knee	Knee Surg Sports Traumatol Arthrosc
363	2015	Kovalak, E	A comparison of continuous femoral nerve block and periarticular local infiltration analgesia in the management of early period pain developing after total knee arthroplasty	Asia	Knee	Acta Orthop Traumatol Turc
364	2015	Lee, JK	The Effect of a Single Dose of Preemptive Pregabalin Administered With CO0-2 Inhibitor: A Trial in Total Knee	Asia	Knee	J Arthroplasty
365	2015	Saglik, Y	Investigation of Effects of Epidural Anaesthesia Combined with General Anaesthesia on the Stress Response in Patients Undergoing Hip and Knee Arthroplasty	Asia	Hip and knee	Turk J Anaesth Reanim
366	2015	Sakai, N	Equivalence of postoperative quadriceps strength during 1 or 0.5 mg ml(-1) levobupivacaine administration for continuous femoral nerve block following total knee arthroplasty: a double-blinded, randomised controlled trial	Asia	Knee	Eur J Anaesthesiol
367	2015	Shah, NA	Adductor Canal Blockade Following Total Knee Arthroplasty—Continuous or Single Shot Technique? Role in Postoperative Analgesia, Ambulation Ability and Early Functional Recovery: A Randomized Controlled Trial	Asia	Knee	J arthroplasty
368	2015	Shen, SY	Analgesic Effects of Intra-Articular Bupivacaine/Intravenous Parecoxib Combination Therapy versus Intravenous Parecoxib Monotherapy in Patients Receiving Total Knee Arthroplasty	Asia	Knee	BioMed Research International
369	2015	Song, MH	Peri-articular injections of local anaesthesia can replace patient-controlled analgesia after total knee arthroplasty: a randomised controlled study	Asia	Knee	International Orthopaedics
370	2015	Vaisha, R	Local Infiltration Analgesia reduces pain and hospital stay after primary TKA : randomized controlled double blind trial	Asia	Knee	Acta Orthop. Belg
371	2015	Olive, DJ	A randomised controlled trial comparing three analgesia regimens following total knee joint replacement: Continuous femoral nerve block, intrathecal morphine or both	Australasia	Knee	Anaesthesia and intensive care
372	2015	Wyatt, MC	Femoral nerve infusion after primary total knee arthroplasty	Australasia	Knee	Bone Joint Res
373	2015	Ali, A	Doubtful effect of continuous intraarticular analgesia after total knee arthroplasty	Europe	Knee	Acta Orthopaedica
374	2015	Andersen, KV	Local infiltration analgesia is not improved by postoperative intra-articular bolus injections for pain after total hip	Europe	Hip	Acta Orthopaedica
375	2015	Frassanito, L	Intravenous infusion of magnesium sulfate and postoperative analgesia in total knee arthroplasty	Europe	Knee	Minerva anestesiologica
376	2015	Hartog	No effect of the infiltration of local anaesthetic for total hip arthroplasty using an anterior approach	Europe	Hip	Bone Joint J
377	2015	Hofstad, JK	Perioperative local infiltration anesthesia with ropivacaine has no effect on postoperative pain after total hip arthroplasty	Europe	Hip	Acta Orthopaedica
378	2015	Kratz, T	Impact of regional femoral nerve block during general anesthesia for hip arthoplasty on blood pressure, heart rate and pain control: a randomized controlled study	Europe	Hip	Technology and Health Care
379	2015	Lunn, TH	Analgesic and sedative effects of perioperative gabapentin in total knee arthroplasty: a randomized, double-blind, placebo-controlled dose-finding study	Europe	Knee	Pain
380	2015	Lunn, TH	Analgesic effect of perioperative escitalopram in high pain catastrophizing patients after total knee arthroplasty: a randomized, double-blind, placebo-controlled trial	Europe	Knee	Anesthesiology
381	2015	Sarridou, DG	Intravenous Parecoxib and Continuous Femoral Block for Postoperative Analgesia after Total Knee Arthroplasty. A Randomized, Double- Blind, Prospective Trial	Europe	Knee	Pain Physician
382	2015	Schotanus, MGM	No advantage of adrenaline in the local in ltration analgesia mixture during total knee arthroplasty	Europe	Knee	Knee Surg Sports Traumatol Arthrosc
383	2015	Spinarelli, A	Pain management after total knee arthroplasty: The good, the bad and the ugly	Europe	Knee	J Biol Reg Homeost Agents
384	2015	Stathellis,A	Periarticular injections with continuous perfusion of local anaesthetics provide better pain relief and better function compared to femoral and sciatic blocks after TKA: a randomized clinical trial	Europe	Knee	Knee Surg Sports Traumatol Arthrosc
385	2015	Clarke, H	Pregabalin reduces postoperative opioid consumption	North America	Hip	Br J Anaesth
386	2015	Jove, M	Sufentanil sublingual tablet system for the management of postoperative pain	North America	Hip and knee	Anesthesiology
387	2015	Jules-Elysee, Km	Patient-controlled epidural analgesia or multimodal pain regimen with periarticular	North America	Hip	J Bone Joint Surg Inc
388	2015	Machi, AT	Discharge Readiness after Tricompartment Knee Arthroplasty: adductor Canal versus Femoral Continuous Nerve Blocks-A Dual-center, Randomized Trial	North America	Knee	Anesthesiology
389	2015	Paul, JE	Randomized controlled trial of gabapentin as an adjunctto perioperative analgesia in total hip arthroplasty	North America	Hip	Can J Anaesth
390	2015	Yadeau, JT	Pregabalin and pain after total knee arthroplasty: a double-blind, randomized, placebo-controlled, multidose trial	North America	Knee	Br J Anaesth
391	2016	Eksandr, A.M	A dose reduction of local anesthetic with addition of dexmedetomidine	Africa	Knee	Egypt J Anaesth
392	2016	Sherif, AA	Dexamethasone as adjuvant for femoral nerve block following knee arthroplasty: a randomized, controlled study	Africa	Knee	Acta Anaesth Scand
393	2016	Bali, C	Postoperative analgesic efficacy of fascia iliaca block..	Asia	Knee	J Clin Anesth
394	2016	Fan, L	Comparison of local infiltration analgesia with femoral nerve block for	Asia	Knee	J Arthroplasty
395	2016	Heo, B. H.	Femoral nervblock for patient undergoing total knee arthroplasty prospective, randomized,	Asia	Knee	Medicine
396	2016	Iwakiri, K	Effect of periarticular morphine injection for total knee arthroplasty: a	Asia	Knee	J Arthroplasty
397	2016	Jianda, X	Impact of preemptive analgesia on inflammatory responses and rehabilitation after total knee	Asia	Knee	Scientific **RepoRts**
398	2016	Kilickaya, R	Comparison of the effects of intrathecal fentanyl and intrathecal morphine on pain in elective total knee replacement	Asia	Knee	Pain Research and Management
399	2016	Kurosaka, K	Local Infiltration Analgesia Versus Continuous Femoral Nerve Block in Pain Relief After Total Knee Arthroplasty: a Randomized Controlled Trial	Asia	Knee	J Arthroplasty
400	2016	Sharma, B	Effect of addition of dexmedetomidine to ropivacaine 0.2% for femoral nerve block in patients undergoing unilateral total knee replacement: a randomised double-blind study	Asia	Knee	Indian Journal of Anaesthesia
401	2016	Sundarathiti, P	Comparison of continuous femoral nerve block (CFNB/SA) and continuous femoral nerve block with mini-dose spinal morphine (CFNB/SAMO) for postoperative analgesia after total knee arthroplasty (TKA): a randomized controlled study	Asia	Knee	BMC anesthesiology
402	2016	Tang, Q	Preoperative ropivacaine with or without tramadol for femoral nerve block in total knee arthroplasty	Asia	Knee	Journal of Orthopaedic Surgery
403	2016	Tsukada, S	The impact of including corticosteroid in a periarticular injection for pain control after total knee arthroplasty	Asia	Knee	Bone Joint J

ID	Publication year	First author	Title	Continent	Type of arthroplasty	Journal
404	2016	Varshney, RK	A prospective randomised double blind study comparing the ef cacy of peri-articular injection with bupivacaine and levobupivacaine for postoperative pain control in total knee arthroplasty	Asia	Knee	Anaesth, Pain & Intensive Care
405	2016	Yang, SA	Oxycodone vs. fentanyl in the treatment of early post-operative pain after total knee replacement: randomized controlled	Asia	Knee	Anesth Pain Med
406	2016	Youm, YS	Preemptive Femoral Nerve Block Could Reduce the Rebound Pain After Periarticular Injection in Total Knee	Asia	Knee	J Arthroplasty
407	2016	Zhu, YZ	Parecoxib prevents early postoperative cognitive dysfunction in elderly patients undergoing total knee arthroplasty	Asia	Knee	Medicine
408	2016	Mulford, JS	Short-term outcomes of local infiltration anaesthetic in total knee arthroplasty: a randomized controlled double-blinded	Australasia	Knee	ANZ J Surg
409	2016	Canata, G. L	Pain management in total knee arthroplasty	Europe	Knee	Joints
410	2016	Francesco, A	Continuous intraarticular and periarticular levobupivacaine for management of pain relief	Europe	Knee	Journal of orthopaedics
411	2016	Hinarejos, P	Local infiltration analgeisa adds no clinical benefit in pain control to peripheral nerve	Europe	Knee	Knee Surg Sports Traumatol Arthrosc.
412	2016	Kadic, L	The effect of pregabalin and s-ketamine in total knee arthroplasty patients: a randomized trial	Europe	Knee	J Anaest Clin Pharm
413	2016	Kearns, R	A randomised, controlled, double blind, non-inferiority trial of ultrasound-guided fascia iliaca block vs. spinal morphine for analgesia after primary hip arthroplasty	Europe	Hip	Anaesthesia
414	2016	McDonald, DA	The technique of delivery of peri-operative analgesia does not affect the rehabilitation or outcomes following total knee	Europe	Knee	Bone Joint J
415	2016	Munteanu, AM	Is there any analgesic benefit from preoperative vs. postoperative administration of etoricoxib in total knee arthroplasty under spinal anaesthesia?: A randomised double-blind placebo-controlled trial	Europe	Knee	Eur J Anaesthesiol
416	2016	Piirainen, A	The Cerebrospinal Fluid Distribution of Postoperatively Administred Dexketoprofen and Etoricoxib and Their Effect on Pain and Inflammatory Markers in Patients Undergoing Hip Arthroplasty	Europe	Hip	Clin Drug Investig
417	2016	Runge, C	The Analgesic Effect of Obturator Nerve Block Added to a Femoral Triangle Block After Total Knee Arthroplasty A Randomized Controlled Trial	Europe	Knee	Reg Anesth Pain Med
418	2016	Sarridou, DG	Parecoxib Possesses Anxiolytic Properties in Patients Undergoing Total Knee Arthroplasty: a Prospective, Randomized, Double-Blind, Placebo-Controlled, Clinical Study	Europe	Knee	Pain Ther
419	2016	Soltesz, S	Intermittent versus continuous sciatic block combined with femoral block for patients undergoing knee arthroplasty. A randomized controlled trial	Europe	Knee	International Orthopaedics
420	2016	Thybo, KH	Effect of lateral femoral cutaneous nerve-block on pain after total hip arthroplasty:a randomised, blinded, placebo-controlledtrial	Europe	Hip	BMC anesthesiology
421	2016	Vilatte, G	Effect of local anaesthetic wound infiltration on acute pain and bleeding after primary total hip arthroplasty: the EDIPO randomised controlled study	Europe	Hip	International Orthopaedics
422	2016	Wiesmann, T	Continuous adductor canal block versus continuous femoral nerve block after total knee arthroplasty for mobilisation capability and pain treatment: a randomised and blinded clinical trial	Europe	Knee	Arch Orthop Trauma Surg
423	2016	Barringtong, JW	No Difference in Early Analgesia Between Liposomal BupivacaineInjection and Intrathecal Morphine After TKA	North America	Knee	Clin Orthop Relat Res
424	2016	Beausang, Dh	A randomized, controlled trial comparing adductor canal catheter	North America	Knee	J Arthroplasty
425	2016	Chan, IA	Dexmedetomidine during total knee arthroplasty performed underspinal anesthesia decreases opioid use: a randomized-controlledtrial	North America	Knee	Can J Anaesth
426	2016	Choi, S	Optimizing Pain and Rehabilitation after Knee Arthroplasty	North America	Knee	Anesth Analg
427	2016	Collis, P.N	Periarticular injection after total knee arthroplasty	North America	Knee	J Arthroplasty
428	2016	Goytizolo, E. A.	The effect of regional analgesia on vascular tone in hip arthroplasty patients	North America	Hip	Musculoskel J Hosp Spec Surg
429	2016	Hadzic, A	Liposome bupivacaine femoral nerve block for postsurgical analgesia after total	North America	Knee	Anesthesiology
430	2016	Jain, Rk	The AAHKS Clinical research Award: liposomal bupivacaine and periarticular injection	North America	Knee	J arthroplasty
431	2016	Nader, A	Single-Dose Adductor Canal Block With Local Infiltrative Analgesia Compared With Local Infiltrate Analgesia After Total Knee Arthroplasty: a Randomized, Double-Blind, Placebo-Controlled Trial	North America	Knee	Reg Anesth Pain Med
432	2016	Sawhney, M	Pain After Unilateral Total Knee Arthroplasty: a Prospective Randomized Controlled Trial Examining the Analgesic Effectiveness of a Combined Adductor Canal Peripheral Nerve Block with Periarticular Infiltration Versus Adductor Canal Nerve Block Alone Versus Periarticular Infiltration Alone	North America	Knee	Anesth Analg
433	2016	Snyder, MA	Improving total knee arthroplasty perioperative pain management using a periarticular injection with bupivacaine liposomal suspension	North America	Knee	Arthroplasty Today
434	2017	Rizk, H	Combined adductor canal and sciatic nerve block compared with local intraarticular infiltration analgesia for total knee arthroplasty: a prospective blinded randomized controlled study	Africa	Knee	Current Orthopaedic Practice
435	2017	Ban, W.R	Effects of periarticular injection on analgesic effects and NSAID	Asia	Hip and knee	Clinics
436	2017	Canakci, E	The effect of unilateral spinal anaesthesia and psoas compartment	Asia	Knee	Pain Res Man
437	2017	Hua, X	Efficacy and safety of ultrasound-guided fascia iliaca compartment block using dex	Asia	Hip	Int J Clin Exp Med
438	2017	Li, D	Effects of multi-site infiltration analgesia on pain management and early rehabilitation compared with femoral nerve or adductor canal block for patients undergoing total knee arthroplasty: a prospective randomized controlled trial	Asia	Knee	International Orthopaedics
439	2017	Li, J	Adding dexmedetomidine to ropivacaine for femoral nerve block inhibits local inflammatory response	Asia	Knee	Minerva anestesiologica
440	2017	Liang, C	Efficacy and safety of 3 different anesthesia techniques used in total hip arthroplasty	Asia	Hip	Med Sci Monit
441	2017	Lu, Y	Comparison of postoperative femoral nerve block, epidural block and intravenous patient-controlled analgesia in pain control and postoperative rehabilitation after total knee arthroplasty	Asia	Knee	Int J Clin Exp Med
442	2017	Mei, B	Peripheral Nerve Block as a Supplement to Light or Deep General Anesthesia in Elderly Patients Receiving Total Hip	Asia	Hip	Clin J Pain
443	2017	Murata-Ooiwa, M	Intravenous Acetaminophen in Multimodal Pain Management for Patients Undergoing Total Knee Arthroplasty: a Randomized, Double-Blind, Placebo-Controlled Trial	Asia	Knee	J arthroplasty
444	2017	Packiasabapathy, SK	Effect of deXmedetomidine as an adjuvant to bupivacaine in femoral nerve block for perioperative analgesia in patients undergoing total knee replacement arthroplasty: a dose-response study	Asia	Knee	Saudi J Anaesth
445	2017	Panwar, S	Comparative evaluation of ropivacaine and fentanyl versus ropivacaine and fentanyl with clonidine for postoperative epidural analgesia in total knee replacement surgery	Asia	Knee	J Clin Diag Res
446	2017	Pinsornsak, P	Multimodal infiltration of local anaesthetic in total knee arthroplasty; is posterior capsular infiltration worth the risk? a prospective, double-blind, randomised controlled trial	Asia	Knee	Bone Joint J
447	2017	Sigirci, A	Pain management in total knee arthroplasty by intraoperative local anesthetic application and one-shot femoral block	Asia	Knee	Indian J Orthop
448	2017	Stav, A	Femoral versus Multiple Nerve Blocks for Analgesia after Total Knee Arthroplasty	Asia	Knee	Rambam Maimonides Medical Journal
449	2017	Tammachote, N	Periarticular multimodal drug injection is better than single anesthetic drug in controlling pain after total knee	Asia	Knee	Eur J Ort Surg Traumatol
450	2017	Tontisirin, N	Parenteral parecoxib provides a similar reduction in opioid requirement to single-shot sciatic nerve block after total knee arthroplasty when combined with continuous femoral nerve block	Asia	Knee	J Med Assoc Thai
451	2017	Wang, CJ	Efficacy of perineural dexamethasone with ropivacaine in adductor canal block for post-operative analgesia in patients undergoing total knee arthroplasty: a randomized controlled trial	Asia	Knee	Exp Ther Med
452	2017	Zhou, M	Adductor canal block in combination with posterior capsular infiltration on the pain control after TKA	Asia	Knee	Ir J Med Sci
453	2017	Camu, F	Parecoxib, propacetamol, and their combination for analgesia	Europe	Hip	Acta Anaesth Scand
454	2017	Desmet, M	A longitudinal suprainguinal fascia iliaca compartment block	Europe	Hip	Reg Anesth Acute Pain
455	2017	Gudmundsdottir, S	Continuous adductor canal blok added to local infiltration analgesia (LIA) after total knee arthroplasty	Europe	Knee	Acta Orthopaedica
456	2017	Kendrisic, M	Analgesic efficacy and safety of four different anesthesia/postoperative analgesia protocols in patients following total hip	Europe	Hip	Vojnosanit Pregl
457	2017	Kuchalik, J	Local infiltration analgesia or femoral nerve block for postoperative pain management in patients undergoing total hip arthroplasty. A randomized, double-blind study	Europe	Hip	Scandinavian Journal of Pain
458	2017	Luna, IE	The Effect of Preoperative Intra-Articular Methylprednisolone on Pain After TKA: a Randomized Double-Blinded Placebo Controlled Trial in Patients With High-Pain Knee Osteoarthritis and Sensitization	Europe	Knee	J Pain
459	2017	Ortiz-Gomez, JR	Postoperative analgesia for elective total knee arthroplasty under subarachnoid anesthesia with opioids: comparison between epidural, femoral block and adductor canal block techniques (with and without perineural adjuvants). Aprospective, randomized, clinical trial	Europe	Knee	Minerva anestesiologica
460	2017	Van Beek, R	In patients undergoing fast track total knee arthroplasty, addition of buprenorphine to a femoral nerve block has no	Europe	Knee	Medicine
461	2017	Wall, PDH	A pragmatic randomised controlled trial comparing the efficacy of a femoral nerve block and periarticular infiltration for early pain relief following total knee arthroplasty	Europe	Knee	Bone Joint J
462	2017	Zinkus, J	Comparison of 2 analgesia modalities in total knee replacement surgery: is there an effect on knee function rehabilitation?	Europe	Knee	Med Sci Monit
463	2017	Alijanipour, P	Periarticular Injection of Liposomal Bupivacaine Offers No Benefit Over Standard Bupivacaine in Total Knee Arthroplasty: a Prospective, Randomized, Controlled Trial	North America	Knee	J Arthroplasty
464	2017	Amundson, Aw	A Three-arm Randomized Clinical Trial Comparing Continuous Femoral Plus Single-injection Sciatic Peripheral Nerve Blocks versus Periarticular Injection with Ropivacaine or Liposomal Bupivacaine for Patients Undergoing Total Knee	North America	Knee	Anesthesiology
465	2017	DeClaire, Jh	Effectiveness of bupivacaine liposome injectable	North America	Knee	J Arthroplasty
466	2017	Johnson, R. L.	Continuous posterior lumbar plexus nerve block versus periarticular injection with ropivacaine or	North America	Hip	J Bone Joint Surg Inc
467	2017	Kayupov, E	Continuous Adductor Canal Blocks Provide Superior Ambulation and Pain Control Compared to Epidural Analgesia for Primary Knee Arthroplasty: a Randomized, Controlled Trial	North America	Knee	J arthroplasty

ID	Publication year	First author	Title	Continent	Type of arthroplasty	Journal
468	2017	Lee, S	A Randomized Non-Inferiority Trial of Adductor Canal Block for Analgesia After Total Knee Arthroplasty: single Injection Versus Catheter Technique	North America	Knee	J Arthroplasty
469	2017	Macrinici, GI	Prospective, Double-Blind, Randomized Study to Evaluate Single-Injection Adductor Canal Nerve Block Versus Femoral Nerve Block: postoperative Functional Outcomes After Total Knee Arthroplasty	North America	Knee	Reg Anesth Pain Med
470	2017	Mont, MA	Local Infiltration Analgesia With Liposomal Bupivacaine Improves Pain Scores and Reduces Opioid Use After Total Knee Arthroplasty: results of a Randomized Controlled Trial	North America	Knee	J arthroplasty
471	2017	O'Neal, JB	Intravenous vs Oral Acetaminophen as an Adjunct to Multimodal Analgesia After Total Knee Arthroplasty: a Prospective, Randomized, Double-Blind Clinical Trial	North America	Knee	J Arthroplasty
472	2017	Perets, I	Intraoperative Infiltration of Liposomal Bupivacaine vs Bupivacaine Hydrochloride for Pain Management in Primary Total Hip Arthroplasty: a Prospective Randomized Trial	North America	Hip	J arthroplasty
473	2017	Politi, JR	Randomized Prospective Trial Comparing the Use of Intravenous versus Oral Acetaminophen in Total Joint Arthroplasty	North America	Hip and knee	J arthroplasty
474	2017	Smith, EB	Periarticular Liposomal Bupivacaine Injection Versus Intra-Articular Bupivacaine Infusion Catheter for Analgesia After Total Knee Arthroplasty: a Double-Blinded, Randomized Controlled Trial	North America	Knee	J Bone Joint Surg Inc
475	2017	Sogbein, OA	Ultrasound-Guided Motor-Sparing Knee Blocks for Postoperative Analgesia Following Total Knee Arthroplasty	North America	Knee	J Bone Joint Surg Inc
476	2017	Sadigursky, D	Local Periarticular Analgesia in Total Knee Arthroplasty	South America	Knee	Acta Ortop Bras
477	2018	Sultan, W. A.	Continuous psoas sciatic blockade for total knee arthroplasty	Africa	Knee	Saudi J Anaesth
478	2018	Aso, K.	Additional benefit of local infiltration of analgesia to femoral nerve block in total knee arthroplasty: double-blind randomized control study	Asia	Knee	Knee Surg Sports Traumatol Arthrosc
479	2018	Bian, Y. Y.	Role of Parecoxib Sodium in the Multimodal Analgesia after Total Knee Arthroplasty: A Randomized Double-blinded Controlled Trial	Asia	Knee	Orthop Surg
480	2018	Kampitakk, W.	Does Adductor Canal Block Have a Synergistic Effect with Local Infiltration Analgesia for Enhancing Ambulation and Improving Analgesia after Total Knee Arthroplasty?	Asia	Knee	Knee Surg Relat Res
481	2018	Kampitakt, W.	Comparison of adductor canal block versus local infiltration analgesia on postoperative pain and functional outcome after total knee arthroplasty: a randomized controlled trial	Asia	Knee	Malaysian orthopaedic journal
482	2018	Kim, M. K.	Comparison of analgesic efficacy of oxycodone and fentanyl after total hip replacement surgery: A randomized controlled	Asia	Hip	Medicine
483	2018	Lee, C.	Pregabalin and Dexmedetomidine Combined for Pain After Total Knee Arthroplasty or Total Hip Arthroplasty Performed Under Spinal Anesthesia	Asia	Hip and knee	Orthopedics
484	2018	Lei, Y	Multiple Low-Dose Dexamethasone Further Improves Clinical Outcomes Following Total Hip Arthroplasty	Asia	Hip	J arthroplasty
485	2018	Liu, J.	Preoperative celecoxib analgesia is more efficient and equally tolerated compared to postoperative celecoxib analgesia in knee osteoarthritis patients undergoing total knee arthroplasty: A randomized, controlled study	Asia	Knee	Medicine
486	2018	Lubis, A. M. T.	Preemptive Analgesia in Total Knee Arthroplasty: Comparing the Effects of Single Dose Combining Celecoxib with Pregabalin and Repetition Dose Combining Celecoxib with Pregabalin: Double-Blind Controlled Clinical Trial	Asia	Knee	Pain Res Treat
487	2018	Miyamoto, S.	The effect of morphine added to periarticular multimodal drug injection or spinal anesthesia on pain management and functional recovery after total knee arthroplasty	Asia	Knee	Journal of orthopaedic science
488	2018	Shi, Z.	Efficacy of multimodal perioperative analgesia protocol with periarticular medication injection and nonsteroidal anti-inflammatory drug use in total knee arthroplasty	Asia	Knee	Nigerian journal of clinical practice
489	2018	Tan, Z.	A comparison of adductor canal block and femoral nerve block after total-knee arthroplasty regarding analgesic effect, effectiveness of early rehabilitation, and lateral knee pain relief in the early stage	Asia	Knee	Medicine
490	2018	Tong, QJ	Comparing adductor canal block with local infiltration analgesia in total knee arthroplasty: A prospective, blinded and randomized clinical trial	Asia	Knee	J Clin Anesth
491	2018	Wu, Y.	Perioperative multiple low-dose Dexamethasones improves postoperative clinical outcomes after Total knee arthroplasty	Asia	Knee	BMC musculoskeletal disorders
492	2018	Yu, Y. L.	Continuous femoral nerve block and patient-controlled intravenous postoperative analgesia on TH1/TH2 in patients undergoing total knee arthroplasty	Asia	Knee	J Biol Reg Homeostatic Agents
493	2018	Zhang, Y.	The Prolonged Analgesic Efficacy of an Ultrasound-Guided Single-Shot Adductor Canal Block in Patients Undergoing Total Knee Arthroplasty	Asia	Knee	Orthopedics
494	2018	Dissanayake, R.	Does Dexamethasone Reduce Hospital Readiness for Discharge, Pain, Nausea, and Early Patient Satisfaction in Hip and Knee Arthroplasty? A Randomized, Controlled Trial	Australasia	Hip and knee	Journal of Arthroplasty
495	2018	Sargant, S.	Extended duration regional analgesia for total knee arthroplasty: a randomised controlled trial comparing five days to three days of continuous adductor canal ropivacaine infusion	Australasia	Knee	Anaesthesia and intensive care
496	2018	Bron, J. L.	No effect of double nerve block of the lateral cutaneous nerve and subcostal nerves in total hip arthroplasty: A randomized controlled trial	Europe	Hip	Acta Orthopaedica
497	2018	Caceres-Sanchez, L.	The use of an intraarticular catheter on fast-track primary knee arthroplasty, is it a step forward?	Europe	Knee	Rev Esp Cir Ortop Traumatol
498	2018	Fenten, M.	Femoral nerve catheter vs local infiltration for analgesia in fast track total knee arthroplasty: short-term and long-term	Europe	Knee	British journal of anaesthesia
499	2018	Jaeger, P	Adductor Canal Block With Continuous Infusion Versus Intermittent Boluses and Morphine Consumption: A Randomized, Blinded, Controlled Clinical Trial	Europe	Knee	Anesth Analg.
500	2018	Manassero, A.	Oral prolonged-release oxycodone/naloxone offers equivalent analgesia to intravenous morphine patient-controlled analgesia after total knee replacement. Arandomized controlled trial	Europe	Knee	Minerva anestesiologica
501	2018	Paglia, A.	Pain management after total knee arthroplasty: a prospective randomized study	Europe	Knee	J Clinical Ort and trauma
502	2018	Tziona, D.	Local infiltration analgesia combined with a standardized multimodal approach including an adductor canal block in total knee arthroplasty: a prospective randomized, placebo-controlled, double-blinded clinical trial	Europe	Knee	J Anesthesia
503	2018	Wiesmann, T.	Ultrasound-guided single injection versus continuous sciatic nerve blockade on pain management and mobilisation after total knee arthroplasty	Europe	Knee	Eur J Anaesthesiol.
504	2018	Biswas, A	Relative contributions of adductor canal block	North America	Knee	Reg Anesth Acute Pain
505	2018	Dixit, V.	Effectiveness of continuous versus single injection femoral nerve block for total knee arthroplasty: A double blinded,	North America	Knee	Knee
506	2018	Fahs, A. M.	Psoas Compartment Block vs Periarticular Local Anesthetic Infiltration for Pain Management After Anterior Total Hip Arthroplasty: A Prospective, Randomized Study	North America	Hip	Journal of Arthroplasty
507	2018	Grosso, M.	Adductor canal block compared with periarticular bupivacaine injection for total knee arthroplasty: a prospective	North America	Knee	J Bone and Joint Surg
508	2018	Hickman, S. R.	Randomized trial of oral versus intravenous acetaminophen for postoperative pain control	North America	Hip and knee	Am J Health-System Pharm
509	2018	Kaczocha, M.	Intrathecal morphine administration reduces postoperative pain and peripheral endocannabinoid levels in total knee arthroplasty patients: a randomized clinical trial	North America	Knee	BMC anesthesiology
510	2018	Kim, D. H.	Addition of Infiltration Between the Popliteal Artery and the Capsule of the Posterior Knee and Adductor Canal Block to Periarticular Injection Enhances Postoperative Pain Control in Total Knee Arthroplasty: A Randomized Controlled Trial	North America	Knee	Anesth Analg
511	2018	Leung, P.	Postoperative continuous adductor canal block for total knee arthroplasty improves pain and functional recovery: A randomized controlled clinical trial	North America	Knee	Journal of Clinical Anesthesia
512	2018	Perry, C.	Intraoperative Psoas Compartment Block vs Preoperative Fascia Iliaca Block for Pain Control After Direct Anterior Total Hip Arthroplasty: a Randomized Controlled Trial	North America	Hip	Journal of arthroplasty
513	2018	Romano, C.	A Randomized Comparison of Pain Control and Functional Mobility between Proximal and Distal Adductor Canal Blocks for Total Knee Replacement	North America	Knee	Anesth Essays Res
514	2018	Schumer, G.	Liposomal Bupivacaine Utilization in Total Knee Replacement Does Not Decrease Length of Hospital Stay	North America	Knee	J Knee Surg
515	2018	Suarez, J. C.	Effectiveness of Novel Adjuncts in Pain Management Following Total Knee Arthroplasty: A Randomized Clinical Trial	North America	Knee	J Arthroplasty
516	2018	Talmo, C. T.	Prospective Randomized Trial Comparing Femoral Nerve Block With Intraoperative Local Anesthetic Injection of Liposomal Bupivacaine in Total Knee Arthroplasty	North America	Knee	Journal of Arthroplasty
517	2018	Turner, J. D.	Single-Injection Adductor Canal Block With Multiple Adjuvants Provides Equivalent Analgesia When Compared With Continuous Adductor Canal Blockade for Primary Total Knee Arthroplasty: A Double-Blinded, Randomized,	North America	Knee	Journal of Arthroplasty
518	2019	Canbek, U.	Continuous adductor canal block following total knee arthroplasty provides a better analgesia compared to single shot: A prospective randomized controlled trial	Asia	Knee	Acta Orthop Traumatol Turc
519	2019	Chandy, V. J.	How effective is periarticular drug infiltration in providing pain relief following Total Knee Replacement as compared to epidural analgesia?	Asia	Knee	J Arthroscopy and Joint Surg
520	2019	Cheng, B. L. Y.	Pre-operative intravenous steroid improves pain and joint mobility after total knee arthroplasty in Chinese population: a double-blind randomized controlled trial	Asia	Knee	Eur J Orthop Surg Traumatol.
521	2019	Cicekci, F.	Ultrasound-guided adductor canal block using levobupivacaine versus periarticular levobupivacaine infiltration after totalknee arthroplasty: a randomized clinical trial.	Asia	Knee	Sao Paulo Med J
522	2019	Diwakar, M	Periarticular regional analgesia in total knee arthroplasty – Efficacy and outcome of single posterior capsular vs multiple site injections	Asia	Knee	J Arthroscopy Joint Surg
523	2019	Gandhi, H. J.	A randomized, controlled trial of comparison of a continuous femoral nerve block (CFNB) and continuous epidural infusion (CEI) using 0.2% ropivacaine for postoperative analgesia and knee rehabilitation after total knee arthroplasty	Asia	Knee	J Anaesthesiol Clin Pharmacol.
524	2019	Gautam, V. K.	Comparative evaluation of periarticular infiltration of two cocktail regimens for analgesia in post-operative patients of total knee replacement	Asia	Knee	J Arthroscopy Joint Surg
525	2019	Iwakiri, K.	Effect of periarticular morphine injection for total hip arthroplasty: a randomised, double-blind trial	Asia	Hip	HIP International

ID	Publication year	First author	Title	Continent	Type of arthroplasty	Journal
526	2019	Kampitak, W.	Opioid-Sparing Analgesia and Enhanced Recovery After Total Knee Arthroplasty Using Combined Triple Nerve Blocks With Local Infiltration Analgesia	Asia	Knee	J Arthroplasty
527	2019	Kim, M. K.	The analgesic efficacy of the continuous adductor canal block compared to continuous intravenous fentanyl infusion with a single-shot adductor canal block in total knee arthroplasty: a randomized controlled trial	Asia	Knee	Korean J Pain
528	2019	Kim, Y. M.	The role of ultrasound-guided single-shot femoral and sciatic nerve blocks on pain management after total knee	Asia	Knee	Knee
529	2019	Koh, I. J.	Duloxetine Reduces Pain and Improves Quality of Recovery Following Total Knee Arthroplasty in Centrally Sensitized Patients: A Prospective, Randomized Controlled Study	Asia	Knee	J Bone Joint Surg Am
530	2019	Kulkarni, M. M.	Randomized Prospective Comparative Study of Adductor Canal Block vs Periarticular Infiltration on Early Functional Outcome After Unilateral Total Knee Arthroplasty	Asia	Knee	J Arthroplasty.
531	2019	Li, D.	Multiple Low Doses of Intravenous Corticosteroids to Improve Early Rehabilitation in Total Knee Arthroplasty: A Randomized Clinical Trial	Asia	Knee	J Knee Surg
532	2019	Lim, Y. C.	A randomised controlled trial comparing adductor canal block and femoral nerve block for knee arthroplasty	Asia	Knee	Singapore medical journal
533	2019	Liu, M	Comparison of the Post-Total Knee Arthroplasty Analgesic Effect of Intraoperative Periarticular Injection of Different Analgesics	Asia	Knee	J Coll Physicians Surg Pak
534	2019	Meng, Z. T.	Epidural morphine improves postoperative analgesia in patients after total knee arthroplasty: A randomized controlled trial	Asia	Knee	PLoS One
535	2019	Park, S.K.	Comparison of bupivacaine plus intrathecal fentanyl and bupivacaine alone for spinal anesthesia with intravenous dexmed	Asia	Knee	Reg Anesth Pain Med
536	2019	Shao, Y.	Comparison of analgesic effect, knee joint function recovery, and safety profiles between pre-operative and post-operative administrations of meloxicam in knee osteoarthritis patients who underwent total knee arthroplasty	Asia	Knee	Ir J Med Sci.
537	2019	Shin, H. J.	Comparison of Intraoperative Sedation With Dexmedetomidine Versus Propofol on Acute Postoperative Pain in Total Knee Arthroplasty Under Spinal Anesthesia: A Randomized Trial	Asia	Knee	Anesth Analg.
538	2019	Takeda, Y.	Evaluating the Effect of Intravenous Acetaminophen in Multimodal Analgesia After Total Hip Arthroplasty: A Randomized Controlled Trial.	Asia	Hip	J Arthroplasty
539	2019	Tammachote, N.	Intravenous Dexamethasone Injection Reduces Pain From 12 to 21 Hours After Total Knee Arthroplasty: A Double-Blind, Randomized, Placebo-Controlled Trial	Asia	Knee	J Arthroplasty.
540	2019	Ueshima, H.	Greater analgesic effect with intermittent compared with continuous mode of lumbar plexus block for total hip arthroplasty: a randomized controlled trial	Asia	Hip	Reg Anesth Pain Med
541	2019	Wang, H.	Effects of Intrathecal Bupivacaine and Bupivacaine Plus Fentanyl in Elderly Patients Undergoing Total Hip Arthroplasty	Asia	Hip	J Coll Physicians Surg Pak
542	2019	Wang, Q.	Efficacy of Single-Shot Adductor Canal Block Combined With Posterior Capsular Infiltration on Postoperative Pain and Functional Outcome After Total Knee Arthroplasty: A Prospective, Double-Blind, Randomized Controlled Study.	Asia	Knee	J Arthroplasty
543	2019	Yang, X.	The effect of dexmedetomidine as adjuvant to ropivacaine 0.1% for femoral nerve block on strength of quadriceps muscle in patients undergoing total knee arthroplasty: A double-blinded randomized controlled trial	Asia	Knee	J Pain Res.
544	2019	Yik, J. H.	Perioperative pregabalin does not reduce opioid requirements in total knee arthroplasty	Asia	Knee	Knee Surg Sports Traumatol Arthrosc
545	2019	Zhao, X. Y.	Ultrasound-Guided Continuous Femoral Nerve Block with Dexmedetomidine Combined with Low Concentrations of Ropivacaine for Postoperative Analgesia in Elderly Knee Arthroplasty	Asia	Knee	Med Princ Pract
546	2019	Zhou, Z. J.	Preoperative intravenous flurbiprofen reduces postoperative pain and inflammatory cytokines in elderly patients after hip	Asia	Hip	Exp Ther Med
547	2019	Tan, N. L.	Impact of Local Infiltration Analgesia on the Quality of Recovery After Anterior Total Hip Arthroplasty: A Randomized, Triple-Blind, Placebo-Controlled Trial	Australasia	Hip	Anesth Analg
548	2019	Borys, M.	Continuous femoral nerve block is more effective than continuous adductor canal block for treating pain after total knee arthroplasty: A randomized, double-blind, controlled trial	Europe	Knee	Medicine
549	2019	Cappelleri, G.	Stimulating versus non-stimulating catheter for lumbar plexus continuous infusion after total hip replacement	Europe	Hip	Minerva anestesiologica
550	2019	El-Boghdadly, K.	Addition of dexamethasone to local infiltration analgesia in elective total hip arthroplasty: a double-blind, randomized	Europe	Hip	Reg Anesth Pain Med.
551	2019	Kastelik, J.	Local infiltration anaesthesia versus sciatic nerve and adductor canal block for fast-track knee arthroplasty: A randomised controlled clinical trial	Europe	Knee	Eur J Anaesthesiol
552	2019	Lyngeraa, T. S.	Comparison of the analgesic effect of an adductor canal block using a new suture-method catheter vs. standard perineural catheter vs. single-injection: a randomised, blinded, controlled study	Europe	Knee	Anaesthesia
553	2019	Mayr, Ho	Pain relieve without impairing muscle function after local infiltration anaesthesia in primary knee arthroplasty: a prospective randomized study	Europe	Knee	Arch Orthop Trauma Surg
554	2019	McCarthy, D.	A comparison of the analgesic efficacy of local infiltration analgesia vs. intrathecal morphine after total knee replacement: A randomised controlled trial	Europe	Knee	Eur J Anaesthesiol
555	2019	Nielsen, N. D.	An Obturator Nerve Block does not Alleviate Postoperative Pain after Total Hip Arthroplasty: a Randomized Clinical	Europe	Hip	Reg Anesth Pain Med
556	2019	Thybo, K. H.	Effect of Combination of Paracetamol and Ibuprofen vs Either Alone on Patient-Controlled Morphine Consumption in the First 24 hours After Total Hip Arthroplasty	Europe	Hip	JAMA
557	2019	Angers, M.	Negative Influence of femoral nerve block on quadriceps strength recovery following total knee replacement: A prospective randomized trial	North America	Knee	Orthop Traumatol Surg Res
558	2019	Chuan, A.	Adductor canal versus femoral triangle anatomical locations for continuous catheter analgesia after total knee arthroplasty: a multicentre randomised controlled study.	North America	Knee	Br J Anaesth
559	2019	Elkassabany, N.	A prospective randomized open-label study of single injection versus continuous adductor canal block for postoperative analgesia after total knee arthroplasty	North America	Knee	The bone & joint journal
560	2019	Goytizolo, E. A.	Addition of Adductor Canal Block to Periarticular Injection for Total Knee Replacement: A Randomized Trial	North America	Knee	J Bone Joint Surg Am
561	2019	Hyland, S. J.	Liposomal Bupivacaine Versus Standard Periarticular Injection in Total Knee Arthroplasty With Regional Anesthesia: A Prospective Randomized Controlled Trial	North America	Knee	J Arthroplasty
562	2019	Kukreja, P.	Anterior quadratus lumborum block analgesia for total hip arthroplasty: a randomized, controlled study	North America	Hip	Reg Anesth Pain Med
563	2019	Marino, J.	Periarticular Knee Injection With Liposomal Bupivacaine and Continuous Femoral Nerve Block for Postoperative Pain Management After Total Knee Arthroplasty: A Randomized Controlled Trial	North America	Knee	J Arthroplasty
564	2019	Meftah, M.	Efficacy of Adductor Canal Block With Liposomal Bupivacaine: A Randomized Prospective Clinical Trial	North America	Knee	Orthopedics
565	2019	Swenson, J. D.	Randomized controlled trial of a simplified adductor canal block performed for analgesia following total knee arthroplasty	North America	Knee	Reg Anesth Pain Med
566	2019	Tan, T. L.	Intraoperative Ketamine in Total Knee Arthroplasty Does Not Decrease Pain and Narcotic Consumption: A Prospective Randomized Controlled Trial	North America	Knee	J Arthroplasty
567	2019	Westrich, G. H.	Intravenous vs Oral Acetaminophen as a Component of Multimodal Analgesia After Total Hip Arthroplasty: A Randomized, Blinded Trial.	North America	Hip	J Arthroplasty
568	2020	Kim, J. K.	Efficacy of Systemic Steroid Use Given One Day After Total Knee Arthroplasty for Pain and Nausea: A Randomized Controlled Study	Asia	Knee	J Arthroplasty.
569	2020	Dizdaveric, A.	Feasibility and efficacy trial comparing liposomal bupivacaine and bupivacaine mixture with bupivacaine only in pre-oper	North America	Knee	J Clin Anesth
570	2020	Qian, Y.	Electromyographic Comparison of the Efficacy of Ultrasound-guided Suprainguinal and Infrainguinal Fascia Iliaca Compartment Block for Blockade of the Obturator Nerve in Total Knee Arthroplasty: A Prospective Randomized	Asia	Knee	Clin J Pain
