# Supplementary material for: Adherence to participant flow diagrams in trials on postoperative pain management after total hip and knee arthroplasty: a methodological review
Source: Trials. 2021 Apr 14;22:280. doi: 10.1186/s13063-021-05233-5 (PMC8048275; doi:10.1186/s13063-021-05233-5)
Supplement: Supplementary file 3 — Additional file 3. Reasons for exclusion of trials. [file 13063_2021_5233_MOESM3_ESM.pdf]

Appendix 3: Excluded articles

Author	Title	Journal	Reason for exclusion
Abdelmawgoud, A	The analgesic efficacy of continuous fascia iliaca block vs continuous psoas compartment block after hip surgery: a comparative study	Egyptian journal of anaesthesia	Includes fracture
Ahdieh, H	Efficacy of oxymorphone extended release in postsurgical pain: a randomized clinical trial in knee arthroplasty	Journal of clinical pharmacology	Includes other types of surgery
Akhondzade, R	Comparison of the analgesic effect of intra-articular and extra-articular injection of morphine and ketamine compound in arthrotomy lower limb surgery under spin	Pakistan journal of medical sciences	Arthrotomy
Akin, A	Preemptive effectiveness of piroxicam in patients undergoing total hip replacement	Turk anesteziyoloji ve reanimasyon	Not English
Aksoy, Ms	Does spinal anesthesia increase the pain and anxiety after total knee arthroplasty? a randomized prospective study	Joint diseases & related surgery	Non-analgesic intervention
Al, Oweidi As	The impact of long-lasting preemptive epidural analgesia before total hip replacement on the hormonal stress response A prospective, randomized, double-blind st	Middle east journal of anaesthesiology	Substudy
Albrecht, E	Single-injection or continuous femoral nerve block for total knee arthroplasty?	Clinical orthopaedics and related research	Duplication
Aldahish, Mm	Regional anaesthesia and postoperative analgesia for major knee surgery: comparison between epidural and combined lumbar plexus and sciatic nerve block	Egyptian journal of anaesthesia	Not found
Anastase, D	A comparative study of efficacy and safety of lornoxicam versus etoricoxib after total knee arthroplasty	Regional Anesthesia and Pain Medicine	Conference Abstract
Andersen, K.V	Comparison between ropivacaine local infiltration analgesia with ketorolac or placebo for total knee replacement surgery	Scandinavian Journal of Pain	Conference Abstract
Andersen, LO	Analgesic efficacy of intracapslar versus intraarticular local anesthetics after knee arthroplasty, A prospective, randomizez, double-blind trial	Acta Anaesthesiologica Scandinavica, Supplement	Conference Abstract
Andersen, LØ	Local anesthetics after total knee arthroplasty: intraarticular or extraarticular administration? A randomized, double-blind, placebo-controlled study	Acta orthopaedica	Randomized after 24 h postoperative
Anderson, SK	Diclofenac in combination with opiate infusion after joint replacement surgery	Anaesthesia and intensive care	Includes age below 18
Arendt-Nielsen, L	Comparing adductor canal block with local infiltration analgesia in total knee arthroplasty: A prospective, blinded and randomized clinical trial	Eur J Pain	Duplication
Aveline, C	Pain and recovery after total knee arthroplasty: a 12-month follow-up after a prospective randomized study evaluating Nefopam and Ketamine for early rehabilitatio	Clinical journal of pain	Obersevational study
Axelsson, K	Postoperative extradural analgesia with morphine and ropivacaine A double-blind comparison between placebo and ropivacaine 10 mg/h or 16 mg/h	Acta anaesthesiologica scandinavica	Hemi-arthroplasty
Aydin, GB	Preemptive effect of dexketoprophen trometamol or paracetamol/tramadol combination on postoperative analgesia	Anestezi dergisi	Not English
Babaiants, Av	Comparative analysis of the efficiency and safety of different types of neuroaxial blocks during total hip replacement	Anesteziologiia i reanimatologiia	Not English
Babu, SC	Postoperative Ultrasound Guided Continuous Femoral Nerve Blockade for Unilateral Total Knee Arthroplasty: A Comparison of 0125% Bupivacaine and 02% Ro	Anesth Analg.	Comparative study
Bagry, H	Effect of a continuous peripheral nerve block on the inflammatory response in knee arthroplasty	Regional anesthesia and pain medicine	Not primary pain study
Bai, SJ	The Effects of Preemptive Intravenous Ketorolac for Total Hip Replacement Patients	Korean journal of anesthesiology	Not English
Bao, Y	Comparison of preincisional and postincisional parecoxib administration on postoperative pain control and cytokine response after total hip replacement	Journal of international medical research	Includes fracture
Baranovic, S	Peripheral regional analgesia with femoral catheter versus intravenous patient controlled analgesia after total knee arthroplasty: a prospective randomized study	Collegium antropologicum	Not found
Barrington, J.W	Postoperative Pain After Primary Total Knee Arthroplasty: Comparison of Local Injection Analgesic Cocktails and the Role of Demographic and Surgical Factors	Journal of Arthroplasty	Non-randomized
Beaupre, La	Impact of a preemptive multimodal analgesia plus femoral nerve blockade protocol on rehabilitation, hospital length of stay, and postoperative analgesia after prim	The scientific world journal	Non-randomized
Beek, R	In patients undergoing fast track total knee arthroplasty, addition of buprenorphine to a femoral nerve block has no clinical advantage	Medicine	Duplication
Bengtsson, M	Postoperative pain relief with intrathecal morphine after major hip surgery	Regional anesthesia	Non-randomized
Berge, R	Femoral nerve block after total knee arthroplasty: comparison of single dose versus continuous infusion. Analgesia and functional recovery	European journal of anaesthesiology.	Conference Abstract
Bertinin, L	Patient-controlled postoperative analgesia in orthopedic surgery: epidural PCA versus intravenous PCA	Minerva anestesiologica	Not English
Bhandari, M	Does perioperative oral rofecoxib therapy improve functional recovery after knee replacement surgery?	CMAJ : Canadian Medical Association journal	Conference Abstract
Bichel, T	Analgesia for total hip replacement: Epidural analgesia versus femoral nerve blockade?	Cahiers d'anesthesiologie	Not English
Borisov, Db	Iliofascial versus epidural block during hip endoprosthesis	Anesteziologiia i reanimatologiia	Not English
Bormann, B	Postoperative pain therapy by transdermal fentanyl	Anasthesie, intensivtherapie, notfallmedizin	Not English
Boucher, C	Intrathecal fentanyl does not modify the duration of spinal procaine block	Canadian journal of anaesthesia	Includes other types of surgery
Bowrey, S	A comparison of 0.2 and 0.5 mg intrathecal morphine for postoperative analgesia after total knee replacement	Anaesthesia	Not proper controlled
Bozkurt, M	Comparing the effects of analgesia techniques with controlled intravenous and epidural on postoperative pain and knee rehabilitation after total knee arthroplasty	Drugs	Not English
Brodner, G	Postoperative analgesia after knee surgery: a comparison of three different concentrations of ropivacaine for continuous femoral nerve blockade	Anesthesia and analgesia	Not proper controlled
Bullingham, Re	Mandatory sublingual buprenorphine for postoperative pain	Anaesthesia	Non-randomized
Busch, C	The Efficacy of Periarticular Multimodal Drug Infiltration in Total Hip Arthroplasty	Journal of bone and joint surgery. American volume	Duplication
Cakan, T	The comparison of two different PCA dose regimens after total knee and hip replacement. [Turkish]	Agri	Not English
Capdevila, X	Effects of perioperative analgesic technique on the surgical outcome and duration of rehabilitation after major knee surgery	Anesthesiology	Includes other types of surgery
Carabine, UA	Extradural clonidine infusions for analgesia after total hip replacement	British journal of anaesthesia	Duplication
Casati, A	72-hour epidural infusion of 0.125% levobupivacaine following total knee replacement: a prospective, randomized, controlled, multicenter evaluation	Acta bio-medica	Randomized after 24 h postoperative
Celik, F	Comparison of the effectiveness of intrathecal bupivacaine and levobupivacaine in hip surgery	Zdravniski vestnik	Randomized after 24 h postoperative
Chan, Ee-Yuee	Functional discharge readiness and mobility following total knee arthroplasty for osteoarthritis: a comparison of analgesic techniques	Arthritis care & research	Not primary pain study
Chaubey, D.	Comparison of continuous femoral nerve block versus local infiltration analgesia as a postoperative analgesia in unilateral total knee arthroplasty	Journal of Clinical and Diagnostic Research	Not found
Chelly, J.E	Continuous femoral blocks improve recovery and outcome of patients undergoing total knee arthroplasty	Journal of Arthroplasty	Non-randomized
Chen, DW	Intra-articular bupivacaine reduces postoperative pain and meperidine use after total hip arthroplasty: a randomized, double-blind study	Journal of arthroplasty	Necrosis
Chen, DW	Continuous intra-articular infusion of bupivacaine for post-operative pain relief after total hip arthroplasty: a randomized, placebo-controlled, double-blind study	European journal of pain (london, england)	Necrosis
Chen, H.	Relative Contributions of Adductor Canal Block and Intrathecal Morphine to Analgesia and Functional Recovery After Total Knee Arthroplasty: A Randomized C	Phys Sportsmed.	Duplication
Chen, Xf	Spinal anesthesia in combination with epidural anesthesia for surgeries of hips and lower limbs	Chinese journal of anesthesiology	Not found
Chen, Y	Efficacy and safety of an intra-operative intra-articular magnesium/ropivacaine injection for pain control following total knee arthroplasty	Journal of international medical research	Not found
Cheville, A	A randomized trial of controlled-release oxycodone during inpatient rehabilitation following unilateral total knee arthroplasty	Journal of bone and joint surgery. American volume	Interventions starts at POD 8
Chinachoti, At	A randomized, double-blinded, placebo-controlled trial of periarticular infiltration of 0.25% bupivacaine for postoperative pain control after total knee	British journal of anaesthesia.	Conference Abstract
Cho, Sy	The Effects of Intrathecal Neostigmine on Postoperative Analgesia in Patients Undergoing Total Knee Replacement	Korean journal of anesthesiology	Not English
Choi, HG	The Analgesic Effect of Postoperative Combined Epidural, Soft Tissue, and Intra-articular Injection of Morphine and Bupivacaine in Patients undergoing Total Kn	Korean journal of anesthesiology	Not English
Christie, Iw	Pain scores after total hip arthroplasty: prospective, randomized, open comparison of two analgesic techniques	Br-j-anaesth	Conference Abstract
Churadze, Bt	Comparative assessment of prolonged femoral nerve blockade and epidural analgesia for postoperative pain in total knee joint arthroplasty	Anesteziologiia i reanimatologiia	Not English
Cien, A.J.	Comparison Between Liposomal Bupivacaine and Femoral Nerve Block in Patients Undergoing Primary Total Knee Arthroplasty	J Neurosurg Spine.	Retrospective study
Colwell, C.W.,Jr	Patient-controlled analgesia compared with intramuscular injection of analgesics for the management of pain after an orthopaedic procedure	J Am Coll Surg.	Includes revision
Corbett, Ar	Use of regional i.v. morphine analgesia in patients undergoing total knee arthroplasty	Br-j-anaesth	Conference Abstract
Courtney, P.M / Manassero, A	Oral prolonged-release oxycodone/naloxone offers equivalent analgesia to intravenous morphine patient-controlled analgesia after total knee replacement	J arthroplasty/Minerva anestesiologica	Not found
Cruz, P	Postoperative analgesia after total knee replacement through three in one block	Revista de la Sociedad Espanola del dolor	Not English
Dahl, J.B.	Continuous blockade of the lumbar plexus after knee surgery - postoperative analgesia and bupivacaine plasma concentrations	Anaesthesia	Fracture surgery
Dahl, J.B.	Comparison of two active analgesic interventions: a suggestion for an 'approximated placebo control'	Trials.	Editorial
Dahl, J.B.	Perineuronal morphine: a comparison with epidural morphine	Anaesthesia	Includes other types of surgery
Dar, Fa	Hyperbaric spinal ropivacaine in lower limb and hip surgery: a comparison with hyperbaric bupivacaine	Journal of anaesthesiology, clinical pharmacology	No pain measurements
de Beer Jde, V.	Efficacy and safety of controlled-release oxycodone and standard therapies for postoperative pain after knee or hip replacement	Can J Anaesth.	Two phase study, one not randomized
De Miguel Rivero, C	Comparative efficacy of oral ibuprofen-arginine, intramuscular magnesic dipyrone and placebo in patients with postoperative pain following total hip replacement	Clinical Drug Investigation	Includes revision
Demirel, I	Comparison of unilateral spinal anesthesia and L_1 paravertebral block combined with psoas compartment and sciatic nerve block in patients to undergo partial hip	European review for medical and pharmacological sciences	Hemi-arthroplasty
Den Hartog, Y.M	Which patient-specific and surgical characteristics influence postoperative pain after THA in a fast-track setting?	BMC Musculoskeletal Disorders	Not proper controlled
Deng, Y	Effect of continuous femoral nerve block combined with periarticular local infiltration analgesia on early operative functional recovery after total knee arthroplasty	Journal of Peking University. Health sciences	Not English
Deniz, S	Comparison of the postoperative analgesic efficacy of an ultrasound-guided fascia iliaca compartment block versus3 in 1 block in hip prosthesis sur	AĞRI	Includes fracture
Dighe, K	Perioperative gabapentin and delirium following total knee arthroplasty: a post-hoc analysis of a double-blind randomized placebo-controlled trial	Journal canadien d'anesthesie [Canadian journal of anaesthesia]	Correspondance
Dimaculangan, D	Periarticular injection and continuous femoral nerve block versus continuous femoral nerve block alone on postoperative opioid consumption and pain control foll	Journal of clinical orthopaedics and trauma	Not found
Dolecek, R	Salmon calcitonin (injection and nasal spray) and placebo in patients undergoing total hip replacement: a double-blind study	Current therapeutic research - clinical and experimental	Not primary pain study
Dominguez-Hervella, F. D.	Combined subarachnoid and epidural block with a single injection, with a modified Tuohy needle and used in hip surgery	Reg Anesth.	Not English
Du, X-p	Analgesic effects of intraarticular cocktail versus intravenous parecoxib injection after total hip arthroplasty	Chinese journal of tissue engineering research	Not English
Duan, L_B	Influence of sufentanial in postoperative patient controlled epidural analgesia in hemorheology after total hip replacement	Journal of jilin university medicine edition	Not English
Dueck, Mh	Addition of sufentanil 1 mug/ml to ropivacaine 0.2% does not improve postoperative analgesia by continuous epidural infusion following total hip replacement	Anasthesiologie und intensivmedizin	Not found
Dysart, S	A Randomized, Multicenter, Double-Blind Study of Local Infiltration Analgesia with Liposomal Bupivacaine for Postsurgical Pain Following Total Knee Arthropla	Open Orthop J.	Protocol
Dysart, S	Local Infiltration Analgesia With Liposomal Bupivacaine Improves Early Outcomes After Total Knee Arthroplasty: 24-Hour Data From the PILLAR Study	Journal of arthroplasty	Substudy
Ebert, Jr	Randomized trial investigating the efficacy of manual lymphatic drainage to improve early outcome after total knee arthroplasty	Archives of physical medicine and rehabilitation	Bilateral surgery
Ekmekci, P	The effect of tramadol adjunct to ropivacaine in continuous femoral nerve block in patients undergoing total knee arthroplasty for postoperative pain treatment. [Tu	Anestezi Dergisi	Not English
Eledjam, J.J	Postoperative analgesia by femoral nerve block with ropivacaine 0.2% after major knee surgery: continuous versus patient-controlled techniques	Knee Surg Sports Traumatol Arthrosc.	Includes other types of surgery
Eloy, J.D	Gabapentin does not appear to improve postoperative pain and sleep patterns in patients who concomitantly receive regional anesthesia for lower extremity orthop	Pain Research and Management	Includes fracture
Fagen, Rj	Effect of ketorolac tromethamine on bleeding and on requirements for analgesia after total knee arthroplasty	Journal of bone and joint surgery. American volume	Bilateral surgery
Fang, Z	Analgesic efficacy of oxycodone-acetaminophen tablets after total knee arthroplasty	Chinese journal of new drugs	Not English
Farag, E	Epidural analgesia improves early rehabilitation after total knee replacement	Journal of clinical anesthesia	Bilateral surgery
Fattorini, F	Peridural analgesic therapy in orthopedic surgery: comparison of ropivacaine and bupivacaine	Clinica terapeutica	Not English
Feng, Y	Effects of a selective cyclooxygenase-2 inhibitor on postoperative inflammatory reaction and pain after total knee replacement	Journal of pain	Bilateral surgery
Feng, Y	Postoperative analgesic and anti-inflammatory effects of rofecoxib after total knee replacement. [Chinese]	Chinese journal of surgery	Not English
Fenten, MGE	No clinically relevant advantage of intrathecal morphine in total hip arthroplasty ?	Acta anaesthesiologica Belgica	Observational study
Fernandez, Castillo A	Post-surgical acute pain complementary assessment in an analgesic power contrast	Revista de la sociedad espanola del dolor	Not English
Ferrante, F	A comparison of the efficacy of pain relief with PCA and standard intramuscular opioid therapy after total knee replacement	Regional Anesthesia	Conference Abstract
Ferrante, FM	Regression of sensory anesthesia during continuous epidural infusions of bupivacaine and opioid for total knee replacement	Anesthesia and analgesia	Not primary pain study
Figueiredo Gonzalez, O	Femoral nerve block versus epidural analgesia after total knee arthroplasty	Dolor	Not found
Florescu, Sc	Perioperative etoricoxib administration reduces postoperative opioid consumption without affecting transfusion requirements in total hip arthroplasty	Romanian journal of anaesthesia and intensive care	Not found
Flory, D.A	Postoperative pain control in total joint arthroplasty: a prospective, randomized study of a fixed-dose, around-the-clock, oral regimen	J Refract Surg.	Includes revision
Forget, P	Less long term pain with an iliofascial than an epidural block after hip arthroplasty	Journal canadien d'anesthesie	Not English
Fransen, M	Preventing chronic ectopic bone-related pain and disability after hip replacement surgery with perioperative ibuprofen. (HIPAID)	Controlled clinical trials	Revision surgery
Fredrickson, Mj	Spinal anaesthesia with adjunctive intrathecal morphine versus continuous lumbar plexus blockade: a randomised comparison for analgesia after hip replacement	Anaesthesia and intensive care	Not found
Fritze, P	Pain therapy using stimulating catheters after total knee arthroplasty	Schmerz (berlin, germany)	Not English
Fusco, P	The addition of continuous wound infusion of local anaesthetics to local infiltration in the management of postoperative pain and rehabilitation after total hip arthro	J Bone Joint Surg Am.	Not found
Galasko, C.S	Trial of oral flupirtine maleate in the treatment of pain after orthopaedic surgery	Arch Orthop Trauma Surg	Revision surgery
Gallardo, J	Efficacy of the fascia iliaca compartment block vs continuous epidural infusion for analgesia following total knee replacement surgery	Revista espanola de anestesiologia y reanimacion	Not English
Gandhi, K	The effect of stimulating versus conventional perineural catheters on postoperative analgesia following ultrasound-guided femoral nerve localization	Journal of clinical anesthesia	Non-analgesic intervention
Gao, W-L	Analgesic effect of femoral and sciatic nerve block under multimodal analgesia in total knee arthroplasty	Chinese journal of tissue engineering research	Not English
Gao, Y.Z	Progress on prevention for anterior knee pain after primary total knee arthroplasty	Saudi J Anaesth.	Not English
Gasanova, I	Ultrasound-guided suprainguinal fascia iliaca compartment block versus periarticular infiltration for pain management after total hip arthroplasty: a randomized cont	Regional anesthesia and pain medicine	Not found

Author	Title	Journal	Reason for exclusion
Gatti, A	Technique of postoperative anesthesia and analgesia in knee surgery	Impegno Ospedaliero, Sezione Scientifica	Not English
Geisler, A	Challenging the multimodal analgesic principle: Data from a cohort of total hip arthroplasty patients in five different hospitals	Acta Anaesthesiologica Scandinavica	Conference Abstract
Gelmanas,A	Postoperative pain management after hip replacement: Efficiency comparison of epidural analgesia and continuous lumbar plexus block	Regional Anesthesia and Pain Medicine	Conference Abstract
Gendy, Ha	Ultrasound guided single injection caudal epidural anesthesia of isobaric bupivacaine with/without dexamethasone for geriatric patients undergoing total hip replace	Egyptian journal of anaesthesia	Includes fracture
Gerig, Hj	Postoperative analgesia with epidural morphine after hip operations (author's transl)	Der anaesthesist	Not English
Gerig, Hj	Epidural buprenorphine for postoperative analgesia after hip operations	Der anaesthesist	Not English
Ghabach, ME	Combined block of the femoral and lateral femoral cutaneous nerves under ultrasound for post-operative analgesia in patients undergoing hip surgery	Middle east journal of anaesthesiology	Includes fracture
Ghosh, S	Adductor canal block and functional recovery after total knee arthroplasty. A double-blind, randomized controlled trial	Regional anesthesia and pain medicine	Not found
Giesinger, J.M	Patient-reported outcome metrics following total knee arthroplasty are influenced differently by patients' body mass index	Knee Surg Sports Traumatol Arthrosc	No active intervention
Gimbel, J	The efficacy and safety of oral immediate-release oxymorphone for postsurgical pain	Anesthesia and analgesia	Osteotomy
Girauedet-Le	Positive effect of patient education for hip surgery: a randomized trial	Arthroscopy.	Non-analgesic intervention
Goitia, Arrola L	Fascia iliaca compartment block for analgesia following total hip replacement surgery	Revista espanola de anestesiologia y reanimacion	Not English
Gómez-Munoz, C	Effect of the '3 in 1' block pre- or post-operatively in postoperative pain following total knee arthroplasty	Revista Espanola de anestesiologia y reanimacion	Not found
Goodchild, Cs	Antinociceptive properties of neurosteroids IV: pilot study demonstrating the analgesic effects of alphadolone administered orally to humans	British journal of anaesthesia	Includes reconstruction
Gotfredsen, K	A 10-year prospective study of single tooth implants placed in the anterior maxilla	Clin Exp Rheumatol.	Includes other types of surgery
Gothesen,O/Gøtesen	Functional outcome and alignment in computer-assisted and conventionally operated total knee replacements: a multicentre parallel-group randomised controlled tr	Asian Cardiovasc Thorac Ann.	Non-analgesic intervention
Goutallier, D	Diflunisal vs. pentazocin after operations on the knee	Rhumatologie - revue international de rhumatologie	Not found
Goyal, N.	The 2012 chitranjan ranawat award: Intraarticular analgesia after TKA reduces pain: A randomized, double-blinded, placebo-controlled, prospective study knee	Clinical Orthopaedics and Related Research	Not found
Grabowska-Gawel, A.	Combined subarachnoid and epidural anaesthesia for endoprosthetoplasty of the knee joint	Zentralbl Gynakol.	Not found
Green, E	Additive effect of continuous adductor canal block and liposomal bupivacaine periarticular injection in total knee arthroplasty	Eur J Orthop Surg Traumatol	Retrospective study
Grenngrass, R. A.	Lumbar plexus and sciatic nerve block for knee arthroplasty: comparison of ropivacaine and bupivacaine	Anesth Analg.	Not primary pain study
Grevstad, U	Effect of adductor canal block versus femoral nerve block on quadriceps strength, mobilization, and pain after total knee arthroplasty: a randomized, blinded study	Physiother Can.	Randomized after 24 h postoperative
Grider Js	Comparison of single shot versus continuous femoral local anesthetic nerve block in patients undergoing knee arthroplasty	Regional anesthesia and pain medicine	Not found
Gruhl, Dw	Propanidid drip as basal anaesthesia during regional anaesthesia (author's transl)	Zeitschrift fur praktische anasthesie, wiederbelebung und IT	Not English
Guan, D	Analgesic effect and security of tramadol/acetaminophen during perioperative total knee arthroplasty	Chinese Journal of Tissue Engineering Research	Not English
Guo, X-Z	Total knee arthroplasty analgesia: gabapentin combined with continuous femoral nerve block	Chinese journal of tissue engineering research	Not English
Gurava Reddy, A	Validating the Role of Steroid in Analgesic Cocktail Preparation for Local Infiltration in Total Knee Arthroplasty: A Comparative Study	Anesth Essays Res 12	Non-randomized
Gurava Reddy, A	Comparison between Multisite Infiltration Analgesia versus Adductor Canal Block for Pain Management in Total Knee Arthroplasty: A Prospective Study	Anesth Essays Res 12	Non-randomized
Guyochin, A	Peridural analgesia and general anesthesia in hip surgery. (Apropos of 34 cases)	Anesthesie, analgesie, reanimation	Not English
Hall, Mj	A randomized controlled trial of postoperative analgesia following total knee replacement: transdermal Fentanyl patches versus patient controlled analgesia (PCA)	European journal of orthopaedic surgery & traumatology	Randomized after 24 h postoperative
Han, Js	Effects of Intravenous and Epidural Patient-Controlled Analgesia on Postoperative Pain and Knee Rehabilitation after a Unilateral Total Knee Replacement	Korean journal of anesthesiology	Not English
Hanna, M. H.	Randomized, double-blind study of the analgesic efficacy of morphine-6-glucuronide versus morphine sulfate for postoperative pain in major surgery	Anesthesiology.	Includes revision
Harsten, A	Total intravenous general anaesthesia vs. spinal anaesthesia for total hip arthroplasty: a randomised, controlled trial	Acta anaesthesiologica scandinavica	Non-analgesic intervention
Harsten, A	Recovery after total intravenous general anaesthesia or spinal anaesthesia for total knee arthroplasty: a randomized trial	British journal of anaesthesia	Non-analgesic intervention
Hartrick, Ct	Fentanyl iontophoretic transdermal system versus morphine intravenous patient-controlled analgesia for pain management following orthopedic surgery	Journal of opioid management	Includes other types of surgery
He, J	Effects and safety of quadratus lumborum block in analgesia after hip arthroplasty	Zhonghua yi xue za zhi	Not English
Herreweghe, I	Longitudinal fascia iliaca compartment block for total hiparthroplasty	Regional anesthesia and pain medicine.	Conference abstract
Himmelseher, S	Small-dose S(+)-ketamine reduces postoperative pain when applied with ropivacaine in epidural anesthesia for total knee arthroplasty	Anesthesia and analgesia	Revision surgery
Horn, Ep	Postoperative pain facilitates nonthermoregulatory tremor	Anesthesiology	Arthroscopy
Horsanli, E	A comparison of epidural anesthesia and lumbar plexus-sciatic nerve block for knee surgery	Clinics (Sao Paulo)	Includes other types of surgery
Hou, X	Effect of adductor canal block combined with local infiltration anesthesia on rehabilitation of primary total knee arthroplasty	Chinese journal of reparative and reconstructive surgery	Not English
Hu, Dh	Epidural butorphanol analgesia in elderly patients undergoing hip replacement	Journal of Southern Medical University	Not English
Hung, Njo Ak	Postoperative patient-controlled epidural analgesia after total knee arthroplasty with 2 mug/mL fentanyl combine with 0.2% ropivacaine or 0.2% levobupivacaine	Pain medicine (united states).	Abstract
Huo, L-R	Characteristics of the effect of preemptive epidural analgesia on catecholamine in patients following total hip replacement	Journal of clinical rehabilitative tissue engineering research	Not English
Hur, MJ	Effect of Intrathecal Morphine for Total Knee Replacement Arthroplasty Elderly Patients	Korean journal of anesthesiology	Not English
Hynes, D	Analgesic efficacy of parenteral paracetamol (propacetamol) and diclofenac in post-operative orthopaedic pain	Acta anaesthesiologica scandinavica	Osteotomy
Ilahi, O.A	Continuous epidural analgesia using fentanyl and bupivacaine after total knee arthroplasty	Clinical Orthopaedics and Related Research	Revision surgery
Ilfeld, Bm	Health-related quality of life after tricompartment knee arthroplasty with and without an extended-duration continuous femoral nerve block	Anesthesia and analgesia	Duplication
Ilfeld, Bm	Health-related quality of life after hip arthroplasty with and without an extended-duration continuous posterior lumbar plexus nerve block	Anesthesia and analgesia	Followup on RCT
Ilfeld, Bm	Long-term pain, stiffness, and functional disability after total knee arthroplasty with and without an extended ambulatory continuous femoral nerve block	Regional anesthesia and pain medicine	Followup on RCT
Ilfeld, Bm	Continuous femoral versus posterior lumbar plexus nerve blocks for analgesia after hip arthroplasty: a randomized, controlled study	Anesthesia and analgesia	Includes other types of surgery
Ilfeld, Bm	Ambulatory continuous posterior lumbar plexus nerve blocks after hip arthroplasty: a dual-center, randomized, triple-masked, placebo-controlled trial	Anesthesiology	Randomized after 24 h postoperative
Illgen, R. L.	Prospective Analysis of a Novel Long-acting Oral Opioid Analgesic Regimen for Pain Control After Total Hip and Knee Arthroplasty	Journal of Arthroplasty	Non-randomized
Jacobson, L	Intrathecal methadone: A dose-response study and comparison with intrathecal morphine 0.5 mg	Pain	Only men
Jeong, MS	Effectiveness of Pain Relief for Femoral Nerve Block in Multimodal Pain Control Protocols in Total Knee Arthroplasty	Journal of the korean orthopaedic association	Not English
Jiang, T	Appliation of multimodal pain control protocol in primary total knee arthroplasty	Chinese Journal of Tissue Engineering Research	Not English
Johansson, S	Analgesic efficacy and safety comparison of ketorolac tromethamine and Doleron for the alleviation of orthopaedic post-operative pain	Journal of international medical research	No specification of orthopedic surgery
Johnson, A	Influence of intrathecal morphine and naloxone intervention on postoperative ventilatory regulation in elderly patients	Acta anaesthesiologica scandinavica	Pain not primary outcome
Jolly, A	Evaluation of efficacy of oral pregabalin in reducing postoperative pain in patients undergoing total knee arthroplasty	Indian journal of orthopaedics	Duplication
Joo, J-H	Is Intra-Articular Multimodal Drug Injection Effective in Pain Management After Total Knee Arthroplasty? A Randomized, Double-Blinded, Prospective Study	Journal of arthroplasty	Correspondance
Jun, W	Different analgesia in elderly patients with total knee arthroplasty: incidence of cognitive dysfunction	Chinese journal of tissue engineering research	Not English
Jung, Mg	Comparison of hydromorphone and fentanyl administered by lumbar epidural as patient-controlled analgesia after total knee arthroplasty	Anesthesia and pain medicine	Not English
Kalliopi, P	Rofecoxibe and Lornoxicam as adjuncts to morphine by patient - controlled analgesia after total knee replacement	The journal of bone and joint surgery (proceedings)	Oral presentation
Kampe, S	Continuous epidural infusion of ropivacaine with sufentanil 1.5 microg x mL(-1) for postoperative analgesia after total knee replacement	Anaesthesia.	Correspondance
Kandikatu, S	Acute post operative pain management in total knee arthroplasty; a comparative study between PCA and PCA with local nerve blocks	The journal of bone and joint surgery (proceedings)	Abstract
Kararmaz, A	The Effects of the Type of Neuroaxial Block on Postoperative Pain after Total HIP Replacement Surgery: spinal vs. Epidural Anaesthesia	Turk anesteziyoloji ve reanimasyon demegi dergisi	Not found
Karvonen, S	Efficacy of oral paracetamol and ketoprofen for pain management after major orthopedic surgery	Methods and findings in experimental and clinical pharmacology	Includes age below 18
Kastanias, P	Patient-controlled oral analgesia for postoperative pain management following total knee replacement	Pain research & management	Intervention on POD2
Kaya, M	Patient-controlled regional analgesia after total hip arthroplasty: psoas compartment block versus three-in one femoral nerve block	Anestezi dergisi	Not English
Kearns, RJ	Intrathecal opioid versus ultrasound guided fascia iliaca plane block for analgesia after primary hip arthroplasty: study protocol for a randomised, blinded, noninfe	Med Sci Monit.	Protocol
Kerpsack, JM	The use of controlled-release versus scheduled oxycodone in the immediate postoperative period following total joint arthroplasty	Orthop Nurs.	Includes revision
Kerrick, JM	Low-dose amitriptyline as an adjunct to opioids for postoperative orthopedic pain: a placebo-controlled trial	Pain	Intervention on night after surgery
Khalili, GR	Comparison of intravenous and epidural patient-controlled analgesia after knee and hip arthroplasty	Journal of isfahan medical school	Duplication
Khalili, GR	Comparison of intravenous and epidural patient-controlled analgesia after knee and hip arthroplasty	Journal of isfahan medical school	Not English
Khanna, M	Treating pain with the lidocaine patch 5% after total knee arthroplasty	PM & r : the journal of injury, function, and rehabilitation	Non-randomized
Kilickan, L	The effects of preemptive intravenous versus preemptive epidural morphine on postoperative analgesia and surgical stress response after orthopaedic procedures	Minerva anestesiologica	Not found
Kim, D	Saphenous (adductor canal) nerve block vs. femoral nerve block for total knee arthroplasty: a novel approach for postoperative analgesia	Regional anesthesia and pain medicine	Not found
Kim, DJ	Effects of dexmedetomidine on smooth emergence from anaesthesia in elderly patients undergoing orthopaedic surgery	BMC anesthesiology	Includes other types of surgery
Kim, JH	A comparison of femoral/sciatic nerve block with lateral femoral cutaneous nerve block and combined spinal epidural anesthesia for total knee replacement arthrop	Korean Journal of Anesthesiology	Observational study
Kim, KH	Comparison of Motor Block of 1.0% Ropivacaine and 0.5% Bupivacaine in Epidural Anesthesia for Hip Arthroplasty Surgery	Korean journal of anesthesiology	Not English
Kim, KH	A Comparison of the Use of Several Concentrations of Bupivacaine with Epidural Volume Extension during Combined Spinal-epidural Anesthesia in Total Knee F	Korean journal of anesthesiology	Not English
Klimscha, W	Hemodynamic and analgesic effects of clonidine added repetitively to continous epidural and spinal blocks	Anesth Analg.	Fractures
Koch, T	Levobupivacaine for epidural anaesthesia and postoperative analgesia in hip surgery: a multi-center efficacy and safety equivalence study with bupivacaine and rop	Der anaesthesist	Includes fracture
Koh, IJ	Preemptive low-dose dexamethasone reduces postoperative emesis and pain after TKA: a randomized controlled study	Clinical orthopaedics and related research	Not primary pain study
Kohan, L	Re: Short-term outcomes of local infiltration anaesthetic in total knee arthroplasty: a randomized controlled double-blinded controlled trial	Anesth Analg.	Editorial
Kostadinova, R	Epidural morphine and ultralow dose naloxone to postoperative analgesia in the orthopaedic surgery. [Bulgarian]	Anaesthesiology and Intensive Care	Not English
Kostamovaara, PA	Ropivacaine 1 mg x ml(-1) does not decrease the need for epidural fentanyl after hip replacement surgery	Acta anaesthesiologica scandinavica	Revision surgery
Kramer, S	Continuous spinal anesthesia versus continuous femoral nerve block for elective total knee replacement	Minerva Anestesiologica	Non-randomized
Krenzel, Ba	Posterior capsular injections of ropivacaine during total knee arthroplasty: a randomized, double-blind, placebo-controlled study	Journal of arthroplasty	Bilateral surgery
Krishnan, SH	Addition of buprenorphine to local anesthetic in adductor canal blocks after total knee arthroplasty improves postoperative pain relief: a randomized controlled tria	Journal of clinical anesthesia	No pain measurements
Kutzner, KP	Postoperative analgesia after total knee arthroplasty: continuous intra-articular catheter vs. continuous femoral nerve block	Der orthopade	Not English
Kuusniemi, K	Prolonged-release oxycodone/naloxone in postoperative pain management: from a randomized clinical trial to usual clinical practice	Journal of international medical research	More than one study
Kytta, J	Comparison between 0.5% bupivacaine with and without glucose in lumbar epidural block for knee surgery	Acta anaesthesiologica scandinavica	Arthroscopy
Langford, RM	Comparison of fentanyl iontophoretic transdermal system and routine care with morphine intravenous patient-controlled analgesia in the management of early posto	British journal of pain	Includes other types of surgery
Latasch, L.	Transdermal fentanyl against postoperative pain	Acta anaesthesiologica belgica	Arthrotomy
Lauretti, G	Postoperative analgesia by intraarticular and epidural neostigmine following knee surgery	J Bone Joint Surg Am.	Arthroscopy
Lauretti, G	The combination of epidural clonidine and S(+)-ketamine did not enhance analgesic efficacy beyond that for each individual drug in adult orthopedic surgery	Journal of clinical anesthesia	Arthroscopy
Lauretti, G	Transdermal nitroglycerine enhances spinal sufentanil postoperative analgesia following orthopedic surgery	Anesthesiology	Arthroscopy
Lauretti, G	Postoperative analgesia by intraarticular and epidural neostigmine following knee surgery	J Bone Joint Surg Am.	Duplication
Lauretti, G	The effect of transdermal nitroglycerin on spinal S(+)-ketamine antinociception following orthopedic surgery	Journal of clinical anesthesia	No specification of orthopedic surgery
Lauretti, G	Study of three different doses of epidural neostigmine coadministered with lidocaine for postoperative analgesia	Anesthesiology	No specification of orthopedic surgery
Lee, Kj	Efficacy of multimodal pain control protocol in the setting of total hip arthroplasty	Clinics in orthopedic surgery	Osteonecrosis
Lee, SH	A Comparison of Patient-Controlled Epidural Analgesia and Continuous Epidural Analgesia after Total Knee Arthroplasty in Elderly Patients over 60 Years of Age	Korean journal of anesthesiology	Not English
Li, L	Analgesics-assisted early functional exercise following minimally invasive total hip arthroplasty	Journal of clinical rehabilitative tissue engineering research	Not English
Litke, J	Diclofenac for treatment of postoperative pain after hip arthroplasty in older patients	Chirurgia narzadow ruchu i ortopedia polska	Not English
Liu, B-S	Multimodal analgesia after total knee arthroplasty	Chinese journal of tissue engineering research	Not English
Liu, J	Peripheral nerve blocks versus general anesthesia for total knee replacement in elderly patients on the postoperative quality of recovery	Clinical interventions in aging	Not found
Liu, SS	A prospective survey of patient-controlled epidural analgesia with bupivacaine and clonidine after total hip replacement	Anesthesia and analgesia	Non-randomized
Liu, Y	Safety of paracetamol and tramadol for perioperative analgesia of total knee arthroplasty. [Chinese]	Chinese Journal of Tissue Engineering Research	Not English
Loland, Vj	Continuous femoral versus posterior lumbar plexus nerve blocks for analgesia following hip arthroplasty: a randomized, controlled study	Regional anesthesia and pain medicine	Duplication
Loncar Stojiljkovic, D.	Comparative postoperative analgesia with femoral nerve block "3-in-1" and with fascia iliaca compartment nerve block after hip alloarthroplasty	Medicinski Casopis	Not English

Author	Title	Journal	Reason for exclusion
Looseley, A	A randomized, observer blinded, trial of intrathecal diamorphine vs femoral nerve block for postoperative analgesia following primary total knee arthroplasty	British journal of anaesthesia	Conference abstract
Lopez, GJ	Analysis of the efficiency and safety of the ileofascial block for postoperatory pain after total knee arthroplasty	Revista de la sociedad espanola del dolor	Not English
Lorenzini, C	Efficacy of ropivacaine compared with ropivacaine plus sufentanil for postoperative analgesia after major knee surgery	Anaesthesia	Includes reconstruction
Lu, H	Continuous analgesia of local infiltration after total knee arthroplasty. [Chinese]	Chinese Journal of Tissue Engineering Research	Not English
Lu, ZD	Analgesic effect of periarticular Ropivacaine infiltration and cyclooxygenase-2 inhibitor following total hip arthroplasty	Journal of clinical rehabilitative tissue engineering research	Not English
Lum, Z.C	Does Local Soft Tissue Infiltration With a Liposomal Bupivacaine Cocktail Have a Synergistic Effect When Combined With Single-Shot Adductor Canal Peripher	Journal of surgical orthopaedic advances	Retrospective study
Lunn, TH	Effect of high-dose preoperative methylprednisolone on recovery after total hip arthroplasty: a randomized, double-blind, placebo-controlled trial	British journal of anaesthesia	Not primary pain study
Macrinici, G	Poster 391 Prospective, Double-blind, Randomized Clinical Trial to Compare Single Shot Adductor Canal Nerve Block Versus Femoral Nerve Block Combined v	Pak J Med Sci.	Poster
Madei, W	Reducing post-operative analgesic requirements by pre-medication with dextromethorphan	Anasthesiologie und intensivmedizin	Not English
Mahoney, OM	The effect of continuous epidural analgesia on postoperative pain, rehabilitation, and duration of hospitalization in total knee arthroplasty	Clinical orthopaedics and related research	Conference abstract
Malam, PP	Comparison of analgesic effect of epidural ropivacaine (0.2%) and ropivacaine (0.2%) with fentanyl (4mcg/ml) in post operative patient of total knee replacement s	International Journal of Pharmaceutical Sciences and Research	Observational study
Malan, T. P., Jr.	Parecoxib sodium, a parenteral cyclooxygenase 2 selective inhibitor, improves morphine analgesia and is opioid-sparing following total hip arthroplasty	Anesth Analg	Revision surgery
Malek, IA	A comparison between the direct anterior and posterior approaches for total hip arthroplasty: the role of an 'Enhanced Recovery' pathway	Arch Phys Med Rehabil.	Non-randomized
Malik, P	Comparative evaluation of epidural fentanyl and butorphanol for postoperative analgesia	Journal of anaesthesiology, clinical pharmacology	No specification of orthopedic surgery
Man, Z. T.	Local injection of bupivacaine following total knee arthroplasty: Analgesia effect and safety evaluation.	Journal of Clinical Rehabilitative Tissue Engineering Research	Not English
Manassero, A.	Comparison of the efficacy of femoral nerve block and fascia iliaca compartment block in patients with total knee replacement	Minerva Anestesiol	Not found
Mangar, D	Knee strength retention and analgesia with continuous perineural fentanyl infusion after total knee replacement: randomized controlled trial	Journal of anesthesia	Not primary pain study
Martin, G	Single-dose extended-release epidural morphine for pain after hip arthroplasty	Journal of opioid management	Hemi-arthroplasty
Martinez-Navas, A.	Postoperative analgesia after total hip arthroplasty using continuous 3-in-1 blockade with stimulating catheter: Ropivacaine 0.2% vs ropivacaine 0.125% vs bupivac	Revista de la Sociedad Espanola del Dolor	Not English
Martinez-Navas, A.	Continuous versus single-dose sciatic nerve block to complement a femoral block after total knee replacement surgery: a randomized clinical trial	Revista espanola de anestesiologia y reanimacion	Not English
Maslanka, MA	Comparison of oral ketorolac, intramuscular morphine, and placebo for treatment of pain after orthopedic surgery	Southern medical journal	Includes other types surgery
Masoudifar, M	Comparison of performance and pain intensity after total knee arthroplasty using general or regional anesthesia	Journal of isfahan medical school	Not English
Matsumoto, S	Transdermal fentanyl patch improves post-operative pain relief and promotes early functional recovery in patients undergoing primary total knee arthroplasty	Archives of orthopaedic and trauma surgery	Intervention starts on POD 2
Mattia, C	Efficacy and safety of fentanyl HCl iontophoretic transdermal system compared with morphine intravenous patient-controlled analgesia for postoperative pain man	European journal of anaesthesiology	Includes other types of surgery
McLeod, GA	Determination of the EC50 of levobupivacaine for femoral and sciatic perineural infusion after total knee arthroplasty	British journal of anaesthesia	Only 1 group
McNamee, Da	Spinal anaesthesia: comparison of plain ropivacaine 5 mg ml(-1) with bupivacaine 5 mg ml(-1) for major orthopaedic surgery	British journal of anaesthesia	Bilateral surgery
McQuay, Hj	Randomized clinical trial of dexketoprofen/tramadol 25 mg/75 mg in moderate-to-severe pain after total hip arthroplasty	Revista de la sociedad espanola del dolor	Duplication
McQuay, Hj	Clinical effects of buprenorphine during and after operation	British journal of anaesthesia	Non-randomized
McQuay, Hj	Randomized clinical trial of dexketoprofen/tramadol 25 mg/75 mg in moderate-to-severe pain after total hip arthroplasty	Revista de la sociedad espanola del dolor	Randomized after 24 h postoperative
Meftah, M	Pain management after total knee arthroplasty using a multimodal approach	Orthopedics	Retracted article
Meier, AW	The use of an intraarticular catheter on fast-track primary knee arthroplasty, is it a step forward?	Reg Anesth Pain Med.	Quasi-randomized
Mejia-Terrazas, Ge	Postoperative analgesia for total knee arthroplasty; a comparative study	Revista mexicana de anestesiologia	Not English
Mendieta Sanchez, J. M.	Efficacy of 0.1 mg of subarachnoid morphine combined with bupivacaine on postoperative analgesia in total hip arthroplasty	Pain	Not English
Milani, P	Multimodal Analgesia in Total Knee Arthroplasty: a Randomized, Double-Blind, Controlled Trial on Additional Efficacy of Periarticular Anesthesia	Journal of arthroplasty	Bilateral surgery
Ming, JL	Effectiveness of Different Pain Control Methods in Patients with Knee Osteoarthritis after Knee Arthroplasty	Biomed research international	Non-randomized
Misiran, KB	The effectiveness of patient-controlled epidural analgesia with ropivacaine 0.165% with fentanyl 2.0 miroc g/ml or levobupivacaine 0.125% with fentanyl 2.0 micro	Middle east journal of anaesthesiology	Duplication
Miyagawa, Y	Methylprednisolone reduces postoperative nausea in total knee and hip arthroplasty	Journal of Clinical Pharmacy and Therapeutics	Non-randomized
Mochizuki, T	Tramadol hydrochloride/acetaminophen combination versus non-steroidal anti-inflammatory drug for the treatment of perioperative pain after total knee arthroplast	J Neurosurg Spine.	Randomized after 24 h postoperative
Moghtadaei, M	Pain Management for Total Knee Arthroplasty: Single-Injection Femoral Nerve Block versus Local Infiltration Analgesia	Interdiscip Perspect Infect Dis	Duplication
Moghtadaei, M	Local infiltration analgesia; an effective method for pain relief and patient's satisfaction after total knee arthroplasty: a randomized clinical trial	Tehran university medical journal	Not English
Mollov, J	The effect of a peri-articular injection mixture of ropivacaine, epinephrine, ketorolac, and morphine in addition to an established multimodal perioperative analgesic	Regional anesthesia and pain medicine	Not found
Montazeri, K	Pre-emptive gabapentin significantly reduces postoperative pain and morphine demand following lower extremity orthopaedic surgery	Singapore medical journal	Arthroscopy
Moodie, JE	A single-center, randomized, double-blind, active, and placebo-controlled study of KAI-1678, a novel PKC-epsilon inhibitor, in the treatment of acute postoperativ	Pain medicine (malden, mass.)	Randomized after 24 h postoperative
Morales-Munoz, C.	Analgesic effect of a single-dose of perineural dexamethasone on ultrasound-guided femoral nerve block after total knee replacement	Rev Bras Anestesiol.	Not English
Motamed, C	Preemptive intravenous morphine-6-glucuronide is ineffective for postoperative pain relief	Anesthesiology	Includes other types surgery
Motifard, M	Pre-emptive injection of peri-articular-multimodal drug for post-operative pain management in total knee arthroplasty: a double-blind randomized clinical trial	International orthopaedics	Quasi-randomized
Mouzopoulos, G.	Analgesic effect of femoral nerve block on postoperative pain and ambulation after total knee arthroplasty	Orthopaedic Journal of Sports Medicine	Abstract
Murphy, Df	Postoperative analgesia in hip surgery. A controlled comparison of epidural buprenorphine with intramuscular morphine	Anaesthesia	Includes fracture
Musclow, Sl	Long-acting morphine following hip or knee replacement: a randomized, double-blind, placebo-controlled trial	Pain research & management	Intervention starts at POD1
Nader, A	A randomized trial of epidural analgesia followed by continuous femoral analgesia compared with oral opioid analgesia on short- and long-term functional recovery	Pain medicine (malden, mass.)	Intervention starts at POD1
Nakagawa, S	Comparative Effects of Periarticular Multimodal Drug Injection and Single-Shot Femoral Nerve Block on Pain Following Total Knee Arthroplasty and Factors Infl	JAMA Surg.	Bilateral surgery
Napier, DE	Postoperative benefits of intrathecal injection for patients undergoing total knee arthroplasty	Orthopaedic nursing/National Association of Orthopaedic Nurses	Retrospective study
NCT/Unknown	Femoral nerve block with liposome bupivacaine for postsurgical analgesia following total knee arthroplasty	Clinicaltrials.gov/show/nct01683071	Clinical trial registration
Nendick, M	Patient satisfaction with post-operative analgesia	Nursing standard (royal college of nursing (great britain)	Quasi-randomized
Neto, J	Methadone patient-controlled analgesia for postoperative pain: a randomized, controlled, double-blind study	Eur J Phys Rehabil Med.	Duplication
Ng, FY	Continuous femoral nerve block versus patient-controlled analgesia following total knee arthroplasty	Journal of orthopaedic surgery (Hong Kong)	Retrospective study
Nielsen, JO	Less pain with epidural morphine after knee arthroplasty	Acta orthopaedica scandinavica	Duplication
Niemi, L	Comparison of intrathecal fentanyl infusion with intrathecal morphine infusion or bolus for postoperative pain relief after hip arthroplasty	Anesthesia and analgesia	Revision surgery
Niemi, L	Technical problems and side effects associated with continuous intrathecal or epidural post-operative analgesia in patients undergoing hip arthroplasty	European journal of anaesthesiology	Revision surgery
Ning, HJ	Low-dose naloxone combined with sufentanil and ropivacaine for postoperative patient-controlled epidural analgesia in elderly patients undergoing total hip replace	Academic Journal of Second Military Medical University	Not English
Nishio, S	Comparison of continuous femoral nerve block, caudal epidural block, and intravenous patient-controlled analgesia in pain control after total hip arthroplasty: a pro	Orthopedic reviews	Duplication
Nishio, S	Comparison of continuous femoral nerve block with and without combined sciatic nerve block after total hip arthroplasty: A prospective randomized study	Orthopedic Reviews	Necrosis
Niskanen, Ro	Bedside femoral block performed on the first postoperative day after unilateral total knee arthroplasty: a randomized study of 49 patients	Journal of knee surgery	Intervention starts at POD1
Niu, XY	Combined spinal-epidural anesthesia with ropicavaine in elderly patients undergoing knee or hip arthroplasty: low incidence of postoperative side effects	Chinese journal of tissue engineering research	Not English
Odoom, Ja	Plasma bupivacaine concentrations following psoas compartment block	S Afr Med J.	Duplication
Odoom, Ja	Plasma bupivacaine concentrations following psoas compartment block	S Afr Med J.	Not primary pain study
Ologoui, D	Levobupivacaine versus ropivacaine for epidural analgesia after total hip replacement	European Journal of Anaesthesiology	Abstract
Omais, M	Epidural morphine and neostigmine for postoperative analgesia after orthopedic surgery	Anesthesia and analgesia	Includes other types surgery
Onal, B	Effects of continuous spinal anesthesia and combined spinal-epidural anesthesia techniques on post-operative pain in total hip replacement patients	Anestezi dergisi	Not found
Panella, L	Control of post-operative pain and rehabilitation compliance of patients undergoing knee replacement	Clinical Practice	Non-randomized
Park, CK	Comparison of Continuous Three-in-One Block and Intravenous Patient-Controlled Analgesia for Postoperative Pain after Total Knee Replacement	Korean journal of anesthesiology	Not English
Park, CK	Optimizing dose infusion of 0.125% bupivacaine for continuous femoral nerve block after total knee replacement	Korean journal of anesthesiology	Only women
Park, Jh	Outcome of Total Knee Arthroplasty Depending on Post-operative Pain Control Methods	Korean journal of anesthesiology	Not English
Paul, JE	Randomized controlled trial of gabapentin as an adjunct to perioperative analgesia in total hip arthroplasty patients	Journal canadien d'anesthesie [Canadian journal of anaesthesia]	Duplication
Pavy, E	Evaluation of postoperative analgesia with continuous iliofascial nerve sheath block after total hip arthroplasty replacement: a pilot study	Anesthesiology	Not English
Paz-Estrada, C	Comparative study of tramadol versus placebo in knee surgery under regional anesthesia	Revista mexicana de anestesiologia	No specification of orthopedic surgery
Peng, WL	Patient-controlled intravenous versus epidural analgesia after major joint replacement	Br J Anaesth.	Not English
Peters, CL	CORR Insights(R): the Chitranjan Ranawat Award: Periarticular injections and femoral & sciatic blocks provide similar pain relief after TKA: a randomized clinical	Clin Orthop Relat Res.	Review
Pettine, Ka	The use of epidural bupivacaine following total knee arthroplasty	Orthopaedic review	Bilateral surgery
Pettine, Ka	The use of epidural bupivacaine following total knee arthroplasty	Orthopaedic review	Duplication
Pinsornsak, P	Comparison of Ramosetron With Ondansetron for Prevention of Intrathecal Morphine-Induced Nausea and Vomiting After Primary Total Knee Arthroplasty: a Ra	Journal of arthroplasty	Anti-emetica
Pitkanen, MT	Comparison of metoclopramide and ondansetron for the prevention of nausea and vomiting after intrathecal morphine	European journal of anaesthesiology	Anti-emetica
Pitkanen, MT	Effect of tropisetron, a 5-HT<inf>3</inf> receptor antagonist, on analgesia and nausea after intrathecal morphine	British Journal of Anaesthesia	Arthroscopy
Poinot, N	Analgesia of Combined Femoral Triangle and Obturator Nerve Blockade Is Superior to Local Infiltration Analgesia After Total Knee Arthroplasty With High-Dose	J Cardiothorac Surg.	One page
Post, ZD	CORR Insights	Clin Orthop Relat Res	Editorial (comment)
Poulsen, Ja	Indomethacin enema in treatment of postoperative pain after knee arthrotomy. A double-blind controlled trial	Ugeskrift for laeger	Not English
Qian, Ww	Application study of periarticular multimodal drug injection in total knee arthroplasty	Zhonghua yi xue za zhi	Not English
Racle, Jp	Bupivacaine spinal anaesthesia in the elderly patient undergoing hip surgery	Annales francaises d'anesthesie et de reanimation	Duplication
Racle, Jp	Prolongation of isobaric bupivacaine spinal anesthesia with epinephrine and clonidine for hip surgery in the elderly	Anesthesia and analgesia	No specification of orthopedic surgery
Racle, JP	Spinal anesthesia with bupivacaine for surgery of the hip in the elderly	Anesth Analg.	Not English
Racle, Jp	Bupivacaine spinal anaesthesia in the elderly patient undergoing hip surgery	Annales francaises d'anesthesie et de reanimation	Not English
Rahimi, M	Effect of additioning of triamcinolon to cocktail of fentanil epinephrinated bupivacaine in periarticular injection for post operative pain and quality improvement afte	Journal of isfahan medical school	Not English
Raimer, C	Continuous psoas and sciatic block after knee arthroplasty: Good effects compared to epidural analgesia or i.v. opioid analgesia: A prospective study of 63 patien	Acta Orthopaedica	Non-randomized
Raj, PP	Comparison of continuous epidural infusion of a local anesthetic and administration of systemic narcotics in the management of pain after total knee replacement s	Anesthesia and analgesia	Bilateral surgery
Rajeev, A	Postoperative pain relief and functional outcome following total knee arthroplasty - a prospective comparative audit of three analgesic regimes	Acta orthopaedica Belgica	Non-randomized
Rakel, BA	Transcutaneous electrical nerve stimulation for the control of pain during rehabilitation after total knee arthroplasty: a randomized, blinded, placebo-controlled trial	Pain	Non-pharmacologic
Raksakietisak, M	Oral rehydration with 10% carbohydrate drink for preventing postoperative nausea and vomiting (PONV) after low dose of spinal morphine	Journal of the Medical Association of Thailand	Anti-emetica
Rasmussen, GL	Intravenous parecoxib sodium for acute pain after orthopedic knee surgery	American journal of orthopedics	Intervention starts at POD1
Rasmussen, GL	Etoricoxib provides analgesic efficacy to patients after knee or hip replacement surgery: a randomized, double-blind, placebo-controlled study	Anesthesia and analgesia	Revision surgery
Remerand, F	The early and delayed analgesic effects of ketamine after total hip arthroplasty: a prospective, randomized, controlled, double-blind study	Anesthesia and analgesia	Hemi-arthroplasty
Ren, L	Effects of two analgesic regimens on the postoperative analgesia and knee functional recovery after unilateral total knee arthroplasty-a randomized controlled trial	Chinese journal of surgery	Not English
Reuben, SS	A prospective randomized trial on the role of perioperative celecoxib administration for total knee arthroplasty: improving clinical outcomes	Anesthesia and analgesia	Retracted article
Reuben, SS	Evaluation of the safety and efficacy of the perioperative administration of rofecoxib for total knee arthroplasty	Journal of arthroplasty	Retracted article
Reynolds, LW	The COX-2 specific inhibitor, valdecoxib, is an effective, opioid-sparing analgesic in patients undergoing total knee arthroplasty	Journal of pain and symptom management	Includes revision
Richards, P	Comparison of the efficacy and safety of dual-opioid treatment with morphine plus oxycodone versus oxycodone/acetaminophen for moderate to severe acute pai	Clinical therapeutics	Randomized after 48 h postoperative
Sakai, N	Patient-controlled bolus femoral nerve block after knee arthroplasty: quadriceps recovery, analgesia, local anesthetic consumption	Acta anaesthesiologica scandinavica	Randomized after 24 h postoperative
Saksena Shrivastava, S.,	To study the effectiveness of continuous femoral block as an alternative for postoperative pain management in patients undergoing total hip arthroplasty	Regional Anesthesia and Pain Medicine	Abstract
Salinas, FV	The effect of single-injection femoral nerve block versus continuous femoral nerve block after total knee arthroplasty on hospital length of stay and long-term func	Anesthesia and analgesia	Intervention at resolution of spinal
Samona, J	Effect of Intraoperative Dexamethasone on Pain Scores and Narcotic Consumption in Patients Undergoing Total Knee Arthroplasty	Orthopaedic Surgery	Retrospective study
Sanchez, Jmm	Analgesic efficacy of 0.1 mg subarachnoid morphine and bupivacaine after total hip arthroplasty	Revista espanola de anestesiologia y reanimacion	Not English
Sanders, JC	Intrathecal baclofen for postoperative analgesia after total knee arthroplasty	Journal of clinical anesthesia	Revision surgery

Author	Title	Journal	Reason for exclusion
Santiveri, Papiol X	Epidural analgesia versus femoral or femoral-sciatic nerve block after total knee replacement: comparison of efficacy and safety	Revista espanola de anestesiologia y reanimacion	Not English
Schultz, P,	Postoperative pain treatment after open knee surgery: continuous lumbar plexus block with bupivacaine versus epidural morphine	Regional anesthesia	Includes other types of surgery
Schwarzkopf, R	Effects of Modification of Pain Protocol on Incidence of Post Operative Nausea and Vomiting	Sao Paulo Med J.	Retrospective study
Sculco, P	The Effect of Perioperative Corticosteroids in Total Hip Arthroplasty: a Prospective Double-Blind Placebo Controlled Pilot Study	Journal of arthroplasty	Pain not primary outcome
Sean, VW	Single-dose periarticular steroid infiltration for pain management in total knee arthroplasty: a prospective, double-blind, randomised controlled trial	Reg Anesth Pain Med.	Duplication
Segado Jimenez, M. I.	Post-operative analgesia in hip surgery: A comparison of 3 techniques. [Spanish]	Revista de la Sociedad Espanola del Dolor	Not English
Segstro, R	The efficacy of indomethacin as a postoperative analgesic following total hip arthroplasty	Can J Anaesth	Duplication
Sell, A	Minimum effective local anaesthetic dose of isobaric levobupivacaine and ropivacaine administered via a spinal catheter for hip replacement surgery	British journal of anaesthesia	No pain outcome
Seo, SS	Comparison of the effect of continuous femoral nerve block and adductor canal block after primary total knee arthroplasty	CiOS Clinics in Orthopedic Surgery	Retrospective study
Serrano, A	Analgesic efficacy of associating a sciatic block to a femoral block in the posotperative period of total knee arthroplasty	European journal of anaesthesiology.	Conference abstract
Shen, B	Effects of perioperative administration of celecoxib on pain management and recovery of function after total knee replacement	Chinese journal of surgery	Not English
Shi, HY	Dexmedetomidine dose for epidural anesthesia in the elderly knee arthroplasty	Chinese journal of tissue engineering research	Not English
Silvanto, M	Comparison of the opioid-sparing efficacy of diclofenac and ketoprofen for 3 days after knee arthroplasty	Acta anaesthesiologica scandinavica	Hemi-arthroplasty
Silvasti, M	Patient-controlled epidural analgesia versus continuous epidural analgesia after total knee arthroplasty	Br-j-anaesth	Duplication
Sinatra, RS	Intravenous acetaminophen for pain after major orthopedic surgery: an expanded analysis	Pain practice	Randomized after 24 h postoperative
Sing, DC	Long-Acting Opioid Use Independently Predicts Perioperative Complication in Total Joint Arthroplasty	J Arthroplasty.	Retrospective study
Singla, NK	iv acetaminophen: efficacy of a single dose forpostoperative pain after hip arthroplasty: subset dataanalysis of 2 unpublished randomized clinical trials	American Journal of Therapeutics	Unpublished trials
Sitsen, E	Postoperative Epidural Analgesia After Total Knee Arthroplasty With Sufentanil 1 mug/mL Combined With Ropivacaine 0.2%, Ropivacaine 0.125%, or Levobupi	Regional Anesthesia and Pain Medicine	Duplication
Sizlan, A	A comparison of the efficacy of bupivacaine and levobupivacaine in patient-controlled epidural analgesia for postoperative pain in patients undergoing knee arthro	Eklem hastaliklari ve cerrahisi = Joint diseases & related surgery	Not English
Sjoegren, P.	[Buprenorphine for postoperative analgesia after total hip alloplasty]	Semin Arthroplasty.	Not English
Skinner, HB	Results of a multimodal analgesic trial involving patients with total hip or total knee arthroplasty	American journal of orthopedics	Retrospective study
Soto, Mesa D	Control of postoperative pain in knee arthroplasty: single dose femoral nerve block versus continuous femoral block	Revista espanola de anestesiologia y reanimacion	Not English
Stober, HD	General anaesthesia or spinal anaesthesia for total hip replacement? Investigations into the patient's acceptance of both methods. [German]	Anaesthesiologie und Reanimation	Not English
Striebel, Hw	Postoperative pain therapy following total endoprosthetic surgery on the hip using a continuous 3-in-1 blockade	Anasthesiologie, Intensivmedizin, Notfallmedizin	Not English
Sun, X	Analgesic effects of continuous fascia iliaca compartment block versus continuous epidural analgesia after total hip arthroplasty. [Chinese]	Chinese Journal of Tissue Engineering Research	Not English
Surange, PN	Comparative evaluation of continuous lumbar paravertebral versus continuous epidural block for post-operative pain relief in hip surgeries	Anesth Essays Res.	Hemi-arthroplasty
Suthersan, M	Local infiltration analgesia versus standard analgesia in total knee arthroplasty	Journal of orthopaedic surgery (Hong Kong)	Audit
Tammachote, N	Periarticular multimodal drug injection is better than single anesthetic drug in controlling pain after total knee arthroplasty	European journal of orthopaedic surgery & traumatology	Duplication
Tang, S	Effect of continuous femoral nerve block on pain score, rehabilitation and stress response after total knee arthroplasty	Regional Anesthesia and Pain Medicine	Abstract
Tang, S	Comparison of the influences of continuous femoral nerve block and patient controlled intravenous analgesia on total knee arthroplasty	Acta academiae medicinae sinicae	Not English
Tanikawa, H	Comparison of local infiltration of analgesia and sciatic nerve block in addition to femoral nerve block for total knee arthroplasty	Journal of arthroplasty	Osteonecrosis
Tanzer, M	Block of the articular branches of the femoral nerve improves early pain control following total hip arthroplasty	Int. J of perioperative ultrasound and applied technologies	Prospective audit
Tarkkila, P	Comparison of intrathecal morphine and continuous femoral 3-in-1 block for pain after major knee surgery under spinal anaesthesia	European journal of anaesthesiology	Ligament surgery
Teuscher, DD	A randomized, controlled trial of total knee replacement	New England journal of medicine	Correspondance
Thobhani, S	Novel regional techniques for total knee arthroplasty promote reduced hospital length of stay: An analysis of 106 patients	Ochsner Journal	Retrospective Study
Thorsell, M	Pain control after total knee arthroplasty: a prospective study comparing local infiltration anesthesia and epidural anesthesia	Orthopedics	Abstract
Thybo, KH	Lateral femoral cutaneous nerve block after total hip arthroplasty: a randomised trial	Acta anaesthesiologica scandinavica	Intervention starts on POD 2
Thybo, Kh	PANSAID - PAracetamol and NSAID in combination: study protocol for a randomised trial	Trials	Protocol
Tian, Y	Pre-emptive parecoxib and post-operative cognitive function in elderly patients	International psychogeriatrics	Includes other types of surgery
Tian, Y	Analgesia after total knee arthroplasty: Comparison of preemptive analgesia and multimodal combined analgesia. [Chinese]	Chinese Journal of Tissue Engineering Research	Not English
Tierney, E	Femoral nerve block with bupivacaine 0.25 per cent for postoperative analgesia after open knee surgery	Canadian Journal of Anaesthesia	Ligament surgery
Tomita, A	Influence of remifentanil on postoperative pain with intravenous patient-controlled analgesia after total knee arthroplasty. [Japanese]	Japanese Journal of Anesthesiology	Not English
Torres Hernandez, R. M.	Epidural vs. Intravenous Nalbuphine for post operative pain control after hip surgery under regional anesthesia. [Spanish]	Revista Mexicana de Anestesiologia	Not English
Tricarico, E	Epidural analgesia compared with peripheral nerve blockade after major knee surgery	Critical care (london, england)	Abstract
Tripuraneni, K,	Local infiltration analgesia in TKA patients reduces length of stay and postoperative pain scores	J Bone Joint Surg Am.	Retrospective study
Tugay, N	Effects on the independence level in functional activities in the early postoperative period in patients with total knee arthroplasty	Neurosciences (Riyadh, Saudi Arabia)	Not found
Tulgar, S	Comparison of Ultrasound-Guided Lumbar Erector Spinae Plane Block and Transmuscular Quadratus Lumborum Block for Postoperative Analgesia in Hip and P	Anesth Essays Res	Femur amputation
Turner, JD	Prevalence and management of intrathecal morphine-induced pruritus in New Zealand Maori healthcare recipients	Br J Anaesth.	Includes other types of surgery
Twiston-Davies, CW	Rectal indomethacin for postoperative pain in orthopaedic surgery. A double-blind study	Journal of bone and joint surgery. British volume	Includes other types of surgery
Utebey, G	[The effects of lumbar plexus block and epidural block on total blood loss and postoperative analgesia in total hip arthroplasty]	Agri	Not English
Vaca, Jm	Block 3 in 1 versus morphine intravenous P.C.A. in postoperative orthopaedic hip surgery pain	Revista de la sociedad espa±ola de dolor	Not English
Van Haelst, I. M.	[Pain treatment following knee and hip replacement surgery]	Mymensingh Med J.	Not English
Villesen, HH	Pharmacokinetics of morphine and oxycodone following intravenous administration in elderly patients	Rev Bras Anestesiol.	No pain measurements
Viscusi, E. R.	Single-dose extended-release epidural morphine for pain following hip arthroplasty	American Journal of Therapeutics	Hemi-arthroplasty
Viscusi, E. R.	A multiple-day regimen of parecoxib sodium 20 mg twice daily provides pain relief after total hip arthroplasty	Anesthesia and analgesia	Revision surgery
Viscusi, E. R.	Forty-eight hours of postoperative pain relief after total hip arthroplasty with a novel, extended-release epidural morphine formulation	Anesthesiology	Revision surgery
von Bormann, B.	[Postoperative pain therapy by transdermal fentanyl]	Int J Radiat Oncol Biol Phys.	Duplication
Wadhwa, A	Large-dose oral dextromethorphan as an adjunct to patient-controlled analgesia with morphine after knee surgery	Anesthesia and analgesia	Includes reconstruction
Wang, H-J	Comparing the analgesic efficacy of continuous femoral nerve blockade and continuous intravenous analgesia after total knee arthroplasty	National medical journal of china	Not English
Wang, H-X	Effect of propacetamol combined with sufentanil for multimodal analgesia in aged patients after total hip replacement operation	Journal of dalian medical university	Not English
Wang, L	Intrathecal ketorolac does not improve acute or chronic pain after hip arthroplasty: a randomized controlled trial	ournal of anesthesia	Pain not primary outcome
Wang, R-G	Analgesic scheme and safety after total knee arthroplasty	Chinese journal of tissue engineering research	Not English
Wang, X	Dexmedetomidine combined with ropivacaine for continuous femoral nerve block improved postoperative sleep quality in elderly patients after total knee arthropla	Zhonghua Yi Xue Za Zhi	Not English
Wang, Y	Analgesic effect of ropivacaine of lumbar epidural anesthesia in elderly patients for hip and knee arthroplasty. [Chinese]	Chinese Journal of Tissue Engineering Research	Not English
Wasinwong, W	Postoperative analgesic effect of intrathecal dexmedetomidine compared to morphine in Bupivacaine spinal block	Journal of the Medical Association of Thailand	Not found
Webb, BT	Periarticular injection of liposomal bupivacaine in total knee arthroplasty	J Neurosurg Spine	Retrospective study
Weber, A	Sciatic nerve block and the improvement of femoral nerve block analgesia after total knee replacement	European journal of anaesthesiology	Observational study
Weng, Wj	Observation of the efficacy of local multimodal analgesic drugs injection in total hip arthroplasty	China journal of orthopaedics and traumatology	Not English
White, Mj	Side effects during continuous epidural infusion of morphine and fentanyl	Canadian journal of anaesthesia	Bilateral surgery
Wik, Ts	Local infiltration anaesthesia in total hip atroplasty. A double-blinded randomised controlled study	HIP international.	Conference abstract
Wilder-Smith, CH	Preoperative adjuvant epidural tramadol: the effect of different doses on postoperative analgesia and pain processing	Pain	Ligament surgery
Wolff, AP	Extradural ropivacaine and bupivacaine in hip surgery	British journal of anaesthesia	Intraoperative
Wong, Cs	Ketamine potentiates analgesic effect of morphine in postoperative epidural pain control	Pharmacopsychiatry.	Different phases
Wu, Zl	Effect of dexmedetomidine on patient-controlled intravenous analgesia with fentanyl in elderly patients after total hip replacement	J South Med Univ	Not English
Xu, M	The manipulation and effects of continuous femoral nerve block after performing a combined spinal and epidural anesthesia	Zhonghua yi xue za zhi	Not English
Xu, X	Efficacy of intra-articular magnesium for postoperative analgesia in total hip arthroplasty	Biomedical reports	Necrosis
Yadeau, JT	Analgesia after total knee replacement: local infiltration versus epidural combined with a femoral nerve blockade: a prospective, randomised pragmatic trial	The bone & joint journal	Duplication
Yeom, JH	Effect of Preemptive Analgesia on Postoperative Pain after Total Knee Arthroplasty	Korean journal of anesthesiology	Not English
Yilmaz, R	Knee arthroplasty and femoral block	Anestezi dergisi	Not English
Yu, Hp	Effect of continuous femoral nerve block in analgesia and the early rehabilitation after total knee replacement	China journal of orthopaedics and traumatology	Not English
Yuksel, B	Continuous epidural analgesia versus combination of single dose sciatic nerve block and continuous femoral analgesia in total knee arthroplasty	Regional Anesthesia and Pain Medicine	Conference abstract
Zhang, B	Short-term effect of multimodal pain relief in total knee arthroplasty	Chinese journal of tissue engineering research	Not English
Zhang, D-S	Effects of different analgesia schemes on joint function and adverse reactions following total knee arthroplasty	Chinese journal of tissue engineering research	Not English
Zhang, J	Effect of periarticular multimodal drug injection on pain after total knee arthroplasty	Journal of clinical rehabilitative tissue engineering research	Not English
Zhao, H	Analgesic outcomes of different methods following total knee arthroplasty	Chinese journal of clinical rehabilitation	Not found
Zhu, X-Y	Analgesic effect of periarticular multimodal drug injection versus nerve block in total knee arthroplasty	Chinese journal of tissue engineering research	Not English
Aasvang, EK	Chronic pre-operative opioid use and acute pain after fast-track total knee arthroplasty	Nutr J.	Observational study
Horasanli, E	A comparison of epidural anesthesia and lumbar plexus-sciatic nerve blocks for knee surgery	Clinics (Sao Paulo)	Fracture surgery
Segstro, R	The efficacy of indomethacin as a postoperative analgesic following total hip arthroplasty	Can J Anaesth	Conference abstract
Kulkarni, M	Effect of Methylprednisolone in Periarticular Infiltration for Primary Total Knee Arthroplasty on Pain and Rehabilitation	J Arthroplasty	Not randomised
Nakagawa, Y	Benefit of subcutaneous patient controlled analgesia after total knee arthroplasty	Asia Pac J Sports Med Arthrosc Rehabil Technol.	Bilateral surgery
Rao, P. B.	Preventive Epidural Analgesia in Bilateral Single-Stage Knee Arthroplasty: A Randomized Controlled Trial	Pain Ther.	Bilateral surgery
Schneider, J.	Narcotic-Free Perioperative Total Knee Arthroplasty: Does the Periarticular Injection Medication Make a Difference?	J Knee Surg.	Not randomised
Saeed, I. H.	Femoral nerve block and adductor canal block for postoperative analgesia of total knee replacement, comparative study	Indian journal of public health research & development.	Could not be found
Srampickal, G. M.	How effective is periarticular drug infiltration in providing pain relief and early functional outcome following total hip arthroplasty?	J Clin Orthop Trauma 10	Fracture surgery
Xiao, K.	Pain Management Using Perioperative Administration of Parecoxib for Total Hip Arthroplasty: A Randomized, Double-Blind, Placebo-Controlled Trial	Pain Physician	Includes caput necrosis
Peng, H. M.	Preemptive Analgesia with Parecoxib in Total Hip Arthroplasty: A Randomized, Double-Blind, Placebo-Controlled Trial	Pain Physician	Fracture surgery
Laoruengthana, A.	Timing of periarticular injection has no effect on postoperative pain and functional recovery in simultaneous bilateral total knee arthroplasty: a prospective randomi	BMC Musculoskelet Disord	Bilateral surgery
