# Supplementary material for: Adherence to participant flow diagrams in trials on postoperative pain management after total hip and knee arthroplasty: a methodological review
Source: Trials. 2021 Apr 14;22:280. doi: 10.1186/s13063-021-05233-5 (PMC8048275; doi:10.1186/s13063-021-05233-5)
Supplement: Supplementary file 4 — Additional file 4. Subgroup analysis of recruitment and retention for trials with adequate participant flow diagrams (continental, interventional and procedural). [file 13063_2021_5233_MOESM4_ESM.pdf]

Appendix 4. Subgroup analysis of recruitment and retention for trials with adequate participant flow diagrams (continental, interventional and procedural)

**Recruitment**	All adequate trials (240)	THA trials (64)	TKA trials (165)
All	73% (44-91%)	68% (44-94%)	74% (43-90%)
Continental (n)			
Europe (86)	65% (43-91%)	61% (44-86%)	64% (40-84%)
Asia (81)	86% (77-93%)	92% (85-96%)	85% (75-92%)
North American (64)	52% (29-71%)	48% (28-83%)	49% (30-71%)
Intervention			
Block (54)	74% (46-91%)	86% (74-94%)	65% (30-89%)
LIA (49)	75% (47-91%)	82% (58-93%)	67% (38-88%)
Neuraxial (8)	78% (48-91%)	87% (85-89%)	70% (44-91%)
Systemic (62)	72% (43-91%)	70% (48-94%)	71% (43-90%)
**Retention**			
All	97% (93-100%)	98% (95-100%)	97% (92-100%)
Continental (n)			
Europe (86)	97% (94-100%)	97% (95-100%)	96% (91-100%)
Asia (81)	99% (95-100%)	100% (98-100%)	98% (94-100%)
North American (64)	96% (90-100%)	98% (90-100%)	97% (90-99%)
Intervention			
Block (54)	97% (93-100%)	98% (94-100)	97% (93-100%)
LIA (49)	97% (93-100%)	98% (94-100%)	97% (91-100%)
Neuraxial (8)	97% (93-100%)	96% (94-98%)	97% (93-100%)
Systemic (62)	97% (93-100%)	98% (93-100%)	97% (93-100%)
**Retention < 95%**			
All	33%	25%	35%
Continental (n)			
Europe (86)	31%	28%	34%
Asia (81)	26%	7%	29%
North American (64)	44%	40%	43%
Intervention			
Block (54)	35%	29%	38%
LIA (49)	35%	19%	46%
Neuraxial (8)	45%	50%	17%
Systemic (62)	38%	43%	33%

Recruitment; randomised:screened ratio. Retention; analysed:randomised ratio.
Trials from Asia reported higher recruitment and retention than trials from other continents. There were no other continental, interventional or procedural differences.
